# New synthetic lipid antigens for rapid serological diagnosis of tuberculosis

**DOI:** 10.1371/journal.pone.0181414

**Published:** 2017-08-14

**Authors:** Alison Jones, Mark Pitts, Juma’a R. Al Dulayymi, James Gibbons, Andrew Ramsay, Delia Goletti, Christopher D. Gwenin, Mark S. Baird

**Affiliations:** 1 School of Chemistry, Bangor University, Bangor, Gwynedd, Wales, United Kingdom; 2 School of Environment, Natural Resources and Geography, Bangor University, Bangor, Gwynedd, Wales, United Kingdom; 3 Special Programme for Research and Training in Tropical Diseases (TDR), World Health Organisation, Geneva, Switzerland; 4 University of St Andrews Medical School, St. Andrews, Scotland, United Kingdom; 5 Translational Research Unit, Department of Epidemiology and Preclinical Research, ‘L. Spallanzani’ National Institute for Infectious Diseases, Rome, Italy; Hospital San Agustin, SPAIN

## Abstract

**Background:**

During pulmonary tuberculosis (PTB) antibodies are generated to trehalose esters of mycolic acids which are cell wall lipids of *Mycobacterium tuberculosis (Mtb)*. Attempts have been made to use these complex natural mixtures in serological tests for PTB diagnosis.

**Aim:**

The aim of this work was to determine whether a serological test based on a panel of defined individual trehalose esters of characteristic synthetic mycolic acids has improved diagnostic accuracy in distinguishing patients with culture positive PTB from individuals who were Mtb culture negative.

**Method:**

One hundred serum samples from well-characterized patients with presumptive tuberculosis, and diagnosed as having pulmonary smear and culture positive TB, or being culture and smear negative were evaluated by ELISA using different combinations of synthetic antigens and secondary antibodies. Using cut-off values determined from these samples, we validated this study blind in samples from a further 249 presumptive TB patients.

**Results:**

With the first 100 samples, detailed responses depended both on the precise structure of the antigen and on the secondary antibody. Using a single antigen, a sensitivity/specificity combination for smear and culture positive PTB detection of 85 and 88% respectively was achieved; this increased to 96% and 95% respectively by a statistical combination of the results with seven antigens. In the blind study a sensitivity/specificity of 87% and 83% was reached with a single antigen. With some synthetic antigens, the responses from all 349 samples were significantly better than those with the natural mixture. Combining the results for seven antigens allowed a distinction between culture positive and negative with a ROC AUC of 0.95.

**Conclusion:**

We have identified promising antigen candidates for serological assays that could be used to diagnose PTB and which could be the basis of a much-needed, simple, rapid diagnostic test that would bring care closer to communities.

## Introduction

Despite advances in the development of diagnostics for pulmonary tuberculosis (PTB), poor case-detection rates remain an obstacle to its control in many low and middle-income countries. Access to any kind of current diagnostic service remains a problem for significant sections of society [1]. Even when accessible, smear microscopy (still the most commonly used PTB diagnostic) has a poor sensitivity and about half the PTB cases that are tested may be a false negative. The newer molecular diagnostic tests for PTB are much more sensitive but, due to the infrastructure required for the equipment, the test facility tends to be centralised and becomes even less accessible to the poorer and more marginalized; their performance remains under review [2]. New tests are needed to allow rapid diagnosis closer to affected communities [3,4], the ideal being a robust, simple serological test that could dramatically improve PTB detection. Such a test has proved elusive. The complexity and variability of the antigenic make-up of *Mycobacterium tuberculosis* (Mtb), of the host immunological response to the organism and the chronic, fluid nature of Mtb, infection and disease all present challenges [5]. An additional challenge has been the lack of well-defined Mtb antigens in sufficient quantities. Either large quantities of poorly defined or undefined mixed antigens, or very small quantities of well-defined extracted antigens, were available. These inadequacies in antigen resources have compromised the development of reproducible assays, and hampered our ability to understand the immunology of infection and disease.

Recently, it has been shown that B cells are functionally altered through the course of tuberculosis (TB), opening new challenges to our understanding on their role in TB pathogenesis [6]. There have been extensive reviews of serodiagnostic assays for TB [7–11]. The World Health Organisation (WHO) has recently summarised a large number of such assays; it indicated that none of these reach the standards of specificity and sensitivity required [12], but identified a clear requirement for a fast and robust serodiagnostic test for point of care use that does reach the required standards. It has defined the target product profile for such an assay [13]. Various studies, e.g. [14–16], have described heterogeneity in responses during infection and disease, and indeed temporal differences in the nature of the responses through the course of infection/disease. A successful serological assay for the diagnosis of TB is likely to be based on a matrix of reactions to a panel of key antigens [17], perhaps differentiated on the basis of the immunoglobulin class involved.

**Fig 1:**
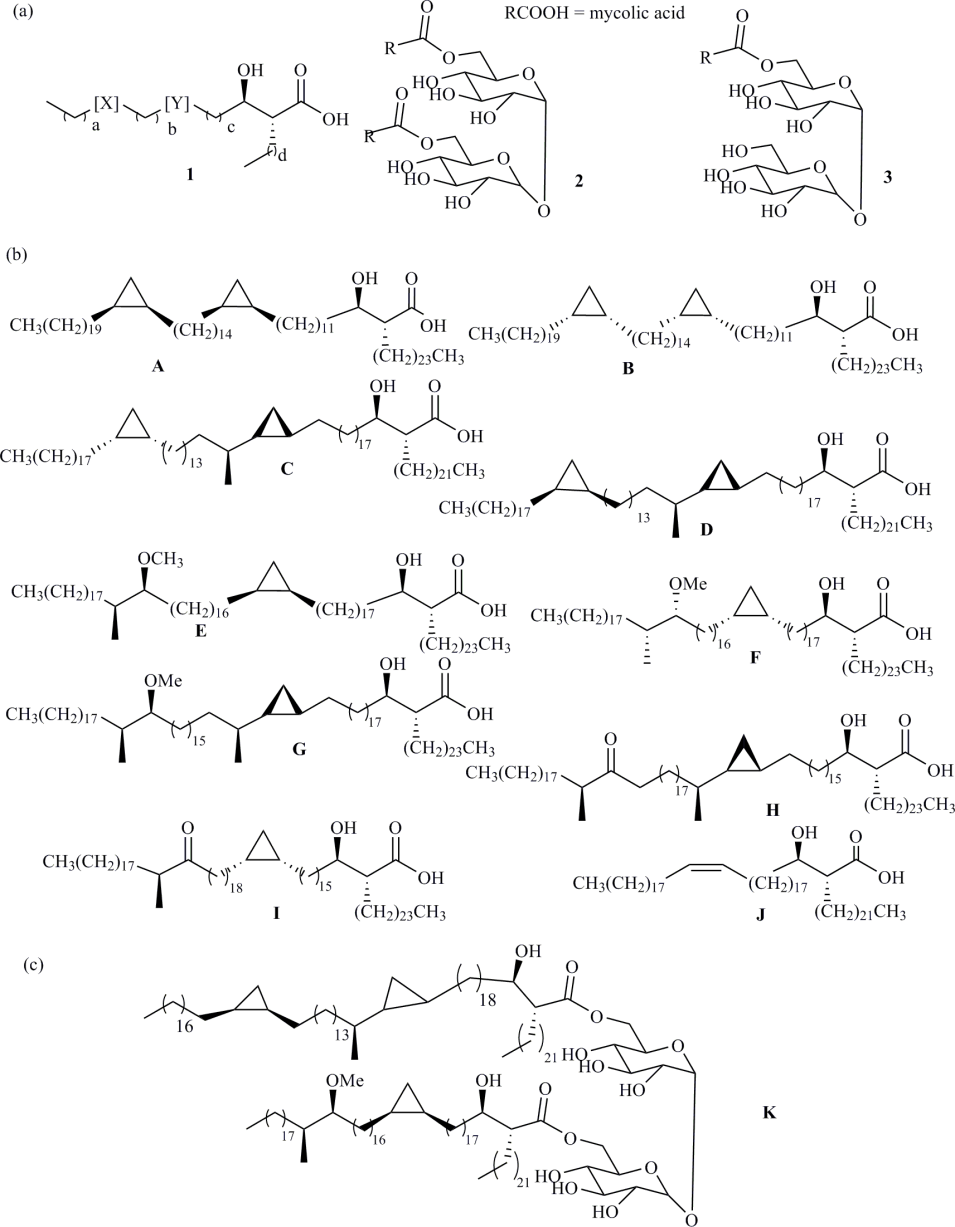
**(a) A general mycolic acid structure (1), together with TDM (2) and TMM (3); (b) Synthetic MA components of TDM and TMM studied in this work; (c) Mixed TDM studied in this work.** Abbreviations: Y, the proximal group, is normally either a *cis*-cyclopropane, a *trans*-cyclopropane with a methyl substituent on the adjacent carbon distal from the hydroxy-acid, a *cis*-alkene, or a *trans*-alkene with a methyl substituent on the adjacent carbon proximal to the hydroxy-acid. X, the distal substituent, is normally a *cis*-cyclopropane (α-MAs), a -CH(CH_3_)CH(OCH_3_)- group (methoxy-MAs) or a–CHCH_3_CO- group (keto-MAs).

Several antigens have been examined and numerous analyses of serodiagnostic assays have been produced [18–26]. The use of natural lipid antigens extracted from *Mtb* cells in serodiagnosis has been reported, primarily by two groups. These antigens, present in the cells of mycobacteria and some related organisms, contain long chain ‘mycolic acids’ (MA) (**1**), which are generally either bound to the cell wall as esters or as, eg., non-wall-bound trehalose dimycolates (**2**) (TDM, ‘cord factor’) and monomycolates (TMM) (**3**) (Fig 1A) [27–29]. They are present as complex mixtures of different carbon chain lengths as well as different structural sub-classes (mainly α-, methoxy and keto- in *Mtb*) that can form a fingerprint of a particular Mycobacterium. The first diagnostic method, using as antigens complex mixtures of TDM (**2**), isolated from Mtb, by ELISA has been reported by Yano [30–41]. The highest average response was for smear-positive PTB patients, followed by atypical mycobacteriosis and then by the smear-negative PTB patients. Serum from healthy controls and cancer patients did not generate responses, leading to an overall sensitivity of 84% and specificity of 100% [30]. The results using IgM as the secondary antibody were less clear. In those treated for PTB, the response declined during treatment, reaching the level of controls after 3 years [32]. Despite excellent reported sensitivity and specificity, obtained primarily using samples from Japan, the method was among those not supported for use by the WHO [12]. The second method, reported by Verschoor uses complex mixtures of free MAs (**1)** derived by hydrolysis of the mycolate esters present in the cells [42–46]. In this case, the accuracy in ELISA is rather lower [47], but significantly improved by using a more complex biosensor system [43–46]. Nonetheless, one advantage of both this and the Yano method is that the antigen-antibody interactions are retained in TB/HIV co-infected patients [31]. The detailed composition of the complex mixtures of individual lipids, MAs (**1**) and their TDMs (**2**) and TMMs (**3**) (Fig 1A), changes during the progression of disease and with the virulence of the mycobacterial strain [48–50]. Moreover, similar, but characteristic, mixtures of MAs are present in other mycobacteria, including common environmental mycobacteria, and in a number of related organisms such as *Rhodococcus* [51,52]. One hypothesis for the failure of these two methods to reach the required standards of accuracy is that serum from patients without TB may be cross-reacting with Mtb antigens as a result of patients’ previous exposure to mycobacteria other than Mtb, or that the antibodies present at the disease stage at which the serum was taken do not match the antigen mixture used for diagnosis. A further complication may be that overall antibody levels change during disease progression or that responses to antibodies to lipids are heterogeneous [53,54]. Many people, though infected with Mtb, do not develop TB disease and are said to have latent TB infection (LTBI). It may be that some antibodies to lipids are generated during LTBI, and this leads to false positives in diagnosing TB disease [55].

Natural TDMs from different classes of MA show differential responses in assays for TB [36]. Given the effect of the balance of these classes, and of stereochemistry within the classes, on disease profile [48–50], one possibility for increasing the accuracy of such assays is to use a diagnostic device based on a set of highly defined antigens, single synthetic sugar esters of MA matching individual components of either *Mtb* or of other mycobacteria [56–59].

This paper describes an evaluation of these synthetic lipid antigens as diagnostic markers for smear and culture positive PTB in comparison with TDM extracted from Mtb cells and, for comparison, bovine TDM (the latter was included to determine whether there were any significant differences in response caused by a different balance in the complex mixtures of MA and TDM between human and bovine samples). It explores the heterogeneity of immunological responses to these antigens among people with presumptive TB [60] in disease-endemic countries; it identifies patterns of Ag/Ig class reactions associated with confirmed pulmonary TB; and it evaluates the use of such patterns to predict confirmed PTB in a set of well-characterized blinded specimens. It seeks to determine whether combining the results of a set of such assays with different antigens can improve diagnostic performance to a level which, when applied on an appropriate platform, might meet the requirements set for a point of care assay [12,13].

## Materials and methods

### Study design

TDR TB Specimen Bank provided serum specimens that had been collected with all the necessary ethical approval and patient consent for distribution to diagnostic test developers [61]. Patients had presented at healthcare facilities in different countries with symptoms suggestive of pulmonary TB (PTB). In this study, those having PTB were positive on culture, and those not having PTB were negative on culture.

**Fig 2:**
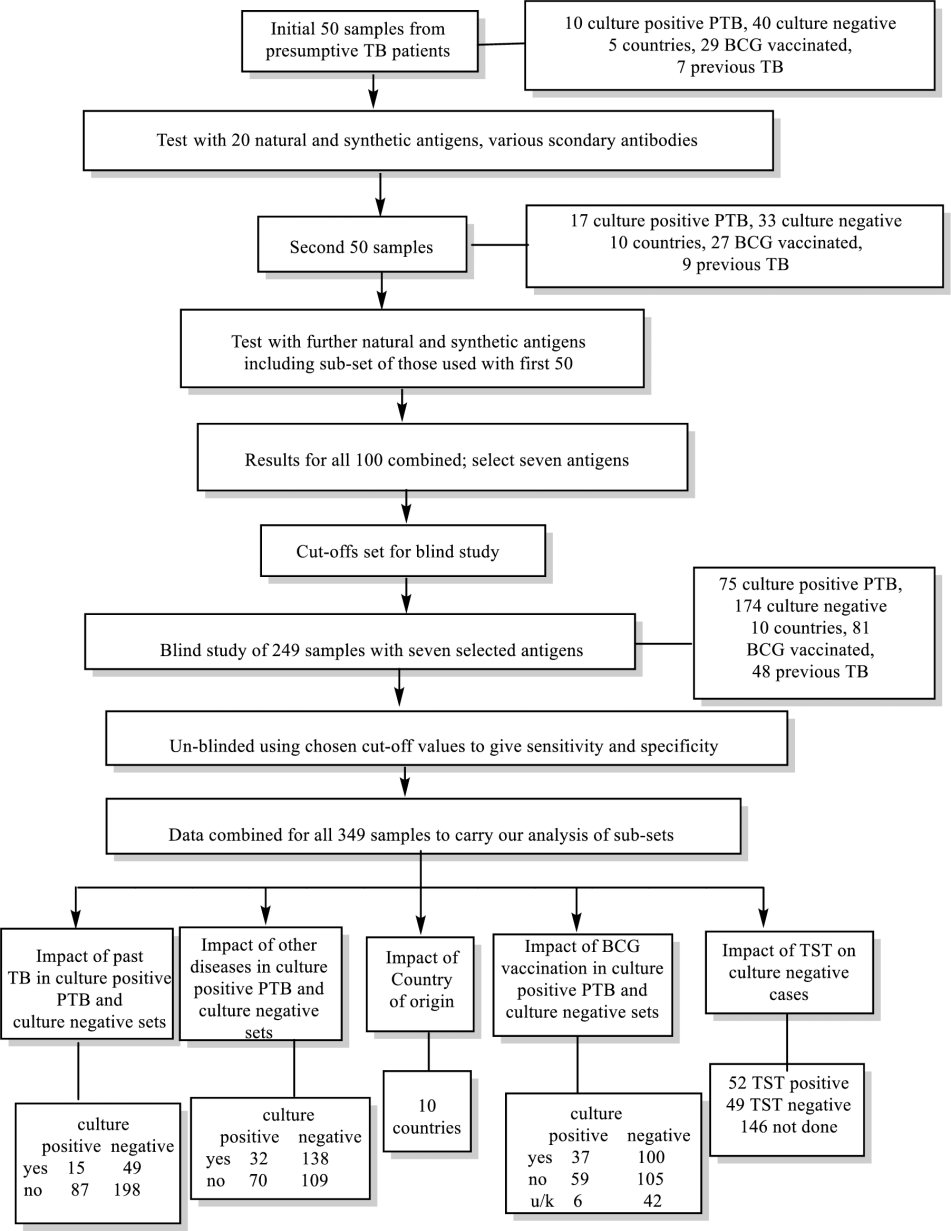
Study format. Abbreviations: TB: tuberculosis; BCG: Bacillus Calmette–Guérin; TST: tuberculin skin test; u/k: unknown.

Within the study format given in Fig 2, these samples were divided into 3 sub-sets. The clinical characteristics of the first two sets of 50 serum samples (n = 100) are given in Tables 1 and 2. These samples were used to develop the assay and were not tested blind. Having developed the assay using these sets, we validated it through blinded testing of a further 249 samples provided from TDR without any accompanying data (Table 3). The results were analysed using cut-off values set for the first 100 samples. WHO/TDR only un-blinded the analysis once results had been reported to them. The results from all 349 samples were considered together in order, where data were available, to analyse the various sub-groups, such as previous TB, positive TST, BCG vaccination, other active diseases, and country effects (S3 File). However it should be noted that data on TST, BCG and other diseases were not complete and not collected in a standardised way across sites due to differences in practices between countries and to limited capacity for the diagnosis of other diseases in many of the countries.

**Table 1:** Demographic and clinical characteristics of the first 50 patients whose sera were evaluated.

	TB	No TB		
	Culture positive	Culture negative	Total	p value
	N. 10	N. 40	N. 50	
Age median (IQR)	28.5 (23–40)	40 (25–54)	38.0 (24–50.5)	.0183
Sex, MALE N (%)	7 (70%)	22 (55)	29 (58)	0.389
AFB smear score/total (%)	Score 0: 0	Score 0: 38 (95)	Score 0/ 38 (76)	<0.001
	Score 1: 4(40)	Score 1: 2 (5)	Score 1/ 6 (12)	
	Score 2: 3(30)		Score 2/ 3 (6)	
	Score 3: 3(30)		Score 3/ 3(6)	
Solid and/or liquid culture positive	10 (100)	0	10 (20)	NA
BCG vaccination recorded N (%)	7 (70)	22 (55)	29 (58)	0.380
Previous TB reported N (%)	1 (10)	6 (15)	7 (14)	0.504
Other diseases N (%)	2 (20)	7 (17.5)	9 (18)	
Site: Pulmonary	8 (80)	40 (100)	48 (96)	0.178
Extra-pulmonary/pulmonary	1 (10)	0	1 (2)	
Unknown	1 (10)	0	1 (2)	
Region of Origin N (%)	Asia 5 (50)	Asia 13 (32.5)	Asia 18 (36)	<0.001
	Africa 5 (50)	Africa 7 (17.5)	Africa 12 (24)	
		S. America 20 (50)	S. America 20 (40)	

**Table 2:** Demographic and clinical characteristics of the second 50 patients whose sera were evaluated.

	TB	No TB		
	Culture positive	Culture negative	Total	p value
	N. 17	N. 33	N. 50	
Age median (IQR)	35 (25.5–43.5)	41 (33–49.5)	38 (28.5–46.5)	0.233
Sex, MALE N (%)	10 (59)	14 (42)	24 (48)	0.189
AFB smear score/total (%)	Score 0: 0	Score 0: 32 (97)	Score 0: 32 (64)	<0.001
	Score 1: 3 (18),	Score 1: 1(3),	Score 1: 4 (8),	
	Score 2: 10 (59)		Score 2: 10 (20),	
	Score 3: 4 (23)		Score 3: 4 (8)	
Solid and/or liquid culture positive	17(100)	0	17 (34)	NA
BCG vaccination recorded N (%)	9 (53)	18 (54.5)	27 (54)	0.953
	2 unknown	4 unknown	6 unknown	
Previous TB reported N (%)	1 (6)	8 (24)	9 (18)	0.171
Other diseases N (%)	5 (17.5)	18 (54.5)	23 (46)	
Site: Pulmonary	16 (94)	33 (100)	49 (98)	
Unknown	1			
TST Positive	3 (17.5)	7 (21)	10 (20)	
Negative	2 (11.8)	7 (21)	9 (18)	
Not done	12	19		
Region of origin	Africa (8)	Africa (9)	Africa (17)	<0.001
	S. America (2)	S. America (13)	S. America (15)	
	Asia (6)	Asia (0)	Asia (6)	
	W.Europe (1)	W. Europe (6)	W. Europe (7)	
		N. America (5)	N. America (5)	

Footnotes: TST: tuberculin skin test

**Table 3:** Demographic and clinical characteristics of the third set of patients: Validation study performed on 249 samples.

	TB	No TB		
	Culture positive	Culture negative	Total	p value
	N. 75	N. 174		
Age median (IQR)	29 (25–43)	47 (29–63)	37.5 (26–57.8)	<0.001
Sex, MALE N (%)	48 (64)	81 (46.5)	129 (52)	<0.001
AFB smear score/total (%)	Score 0: 2 (3)	Score 0: 173 (99)	Score 0: 175 (70)	<0.001
	Score 1: 18 (24)	Score 1: 1(1)	Score 1: 19 (7.5)	
	Score 2: 17 (22.5)		Score 2: 17 (7)	
	Score 3: 38 (50.5)		Score 3: 38 (15)	
Solid and or liquid culture positive	75(100)	0 (0)	75 (30.1)	<0.001
BCG vaccination recorded N (%)	21 (28)	60 (34.5)	81 (32.5)	0.001
Unknown	4	36		
Previous TB reported N (%)	13 (17)	35 (20)	48 (19)	0.552
Other diseases N (%)	26 (35)	113 (65)	139 (56)	
Site (number, percent) Pulmonary	74 (98.5)	173 (99.5)	247 (99)	0.563
Extra-pulmonary + pulmonary	1 (1.5)	1 (0.5)	2 (1)	
TST Positive	6 (8)	45 (26)	51 (20.5)	0.216
Negative	1 (1.5)	42 (24)	43 (17)	
Not done	68	87	155	
Region of origin	Asia (24)	Asia (5)	Asia (29)	<0.001
	S. America (21)	S. America (52)	S. America (73)	
		N. America (29)	N. America (29)	
	Africa (27)	Africa (58)	Africa (85)	
	W. Europe (3)	W. Europe (30)	W. Europe (33)	

Footnotes. TST: tuberculin skin test

### The serum samples

The TDR TB Specimen Bank [61] samples were collected from 349 patients with respiratory symptoms suggestive of PTB in 10 countries. No specific treatment had been started in any of the patients enrolled. Detailed information was available on the patient profile, the country of origin, and the symptoms, as well as the microbiology on solid and/or liquid culture, and, in some cases of follow-up observations. Clinical data also included chest X-ray, where available. Of the 349 patients, 102 had culture positive PTB. Of these 100 were also smear positive (Fig 2; Tables 1–3). The 247 patients who were considered not to have PTB were all culture negative. Four of these patients gave weakly positive smears but nevertheless were considered not to have PTB on the basis of negative cultures. All culture negative patients were followed up for two months and found still to be negative. Eleven of the negatives were given treatment and three improved, five showed no change, one was worse and for two there was no follow up. Investigations included repeat cultures if the respiratory symptoms had persisted. Approximately twice as many negative specimens as positive specimens were provided, because of the development and early validation aims of the study.

### The assay

ELISA were carried out on 96-well flat-bottomed polystyrene micro-plates by slight modification of methods reported earlier [31, 45]. Antigens were dissolved in hexane to give a concentration 15 μg/ml. 50 μl of this solution was added to each well, and the solvent was left to evaporate at room temperature. Control wells were coated with hexane (50 μl/well) only. Blocking was achieved by adding 400 μl of 0.5% casein/PBS buffer (pH 7.4) to each well, and the plates were incubated at 25°C for 30 minutes. The buffer was aspirated and any excess was flicked-out until the plates were dry. Serum (1 in 20 dilution in casein/PBS buffer, unless otherwise stated) (50 μl/well) was added and incubated at 25°C for 1 hour. The plates were washed with 400 μl casein/PBS buffer 3 times using an automatic washer, and any excess buffer was flicked out onto a paper towel until dry. Secondary antibody (all peroxidase conjugated; produced in goat, diluted in casein/PBS buffer, 50 μl/well) [from Sigma Aldrich UK: A0170 (Anti-Human IgG (Fc specific),1:2000); A8667 (Anti-Human IgG (whole molecule), 1:1000); A6029 (Anti-Human IgG (γ-chain specific), 1:2000); A6907 (Anti-Human IgM (μ-chain specific), 1:333); A0295 (Anti-Human IgA (α-chain specific), 1:1667] was added, and incubated at 25°C for 30 minutes. The plates were again washed 3 times with 400 μl casein/PBS buffer using an automatic washer, and any excess buffer was again flicked out. OPD substrate (50 μl / well) (o-phenylenediamine (1 mg/ml) and H_2_O_2_ (0.8 mg/ml) in 100 mM citrate buffer) was then added, and the plates were incubated for a further 30 minutes at 25°C. The colour reaction was terminated by adding 2.5 M H_2_SO_4_ (50 μl/well), and the absorbance was read at 492 nm. Each measurement was carried out in quadruplicate and an average was taken.

The results presented are the un-modified absorbance figures recorded at 492 nm and, do not have the absorbance at 630 nm, or the blank for a plate with no serum subtracted (S1 File).

### The antigens

Natural human and bovine TDM were purchased from Sigma-Aldrich UK. Synthetic antigens were prepared as previously described [56–59]. Their structures are given in Fig 1B/1C and Table 4.

**Table 4:** Synthetic trehalose dimycolates (TDMs), trehalose monomycolates (TMMs) and model TDMs used.

	Derived TDM		Derived TMM	
Mycolic acid (Fig 1B)	Compound number	Reference	Compound number	Reference
**A**	**4**	54	**5**	54
**B**	**6**	54	**7**	54
**C**	**8**	55	**9**	55
**D**	**10**	55	**11**	55
**E**	**12**	54	**13**	54
**F**	**14**	54	**15**	54
**G**	**16**	54	**17**	54
**H**	**18**	54	**19**	54
**I**	**20**	54	**21**	54
**J**	**22**	56		
**CH**_**3**_**(CH**_**2**_**)**_**22**_**COOH**	**23**	55		
**CH**_**3**_**(CH**_**2**_**)**_**20**_**COOH**	**24**	55		
**K (Fig 1C)**	**25**	54		

### Statistical methods

Statistical testing of the sample demographics was carried out using the coin package in R using an exact Wilcoxon-Mann-Whitney test for continuous data and a Chi-Squared test based on a Monte Carlo resampled distribution for the contingency data [62]. For the data analysis based on multiple antigen response and disease status, a training set was formed from the first two data sets (in total 100 samples, with 27 smear and culture positive PTB cases). Validation was done from the third data set in which the disease status was blind at the time of classification. Classification of PTB status based on assay response levels was estimated using tree based Random Forest and Generalised Boosted Model (GBM) classifiers [63,64], trained with the training set in R [65]. An initial screen of the antigens selected those showing a variation of response across the cases and evidence of discrimination between TB status. From the training, a subset of 7 of antigens was selected based on variable importance and a new classifier estimated from the training set using only these antigens. Predictions of disease status were then made for the validation data using this classifier. ROC curves and AUC values were estimated with the pROC package [66] in R [6]. Significance of pairwise differences between AUC values was estimated using the DeLong’s test [67] implemented in the pROC R package. Optimal diagnostic cut-off points were determined using Youden’s J statistic which maximises sensitivity + specificity.

## Results

The experimental ELISA was based on TDMs (structures **2**) or TMMs (structures **3**) (Fig 1, Table 4) prepared from single synthetic MAs containing specific combinations of *cis*- or *trans*-cyclopropane and different oxygenated functionality, patterns known to have different effects on innate immune system responses and to change disease virulence [48–50].

### Results for the initial 50 serum samples

Natural human and bovine TDM and a number of synthetic TDMs and TMMs were examined using a set of 50 serum samples and, in some cases, several different secondary antibodies. Commercial natural human (n15) and bovine TDM (n20) gave significantly different medians with smear and culture positive PTB and culture negative sets using IgG (whole) secondary antibody (p < .001), and AUCs of 0.86 and 0.88 on ROC analysis (Table 5; Fig A in S2 File). The human TDM gave slightly lower AUCs with IgG(Fc) (0.82) and IgG(gamma) (0.80) secondary antibodies, though the medians were significantly different (p = 0.001 and 0.003). The medians for the two sets (Fig B in S2 File) were not significantly different with IgM and IgA secondary antibody (n18 and n19) (p = 0.616 and 0.050) and the AUCs were lower (0.55 and 0.70) (Table 5).

**Table 5:** Results of the ELISA with first set of 50 human sera (10 Mtb culture positive PTB and 40 Mtb culture negative patients) using the different antigen/antibody combinations.

				TB	No TB				
Type of compound^¥¥^	Compound number	Method^#^	2^nd^ Ab used	Smear and culture positive PTB ^##^	Culture negative^##^	p value of the comparisons of medians ELISA results from culture positive PTB and culture negative samples	ROC AUC^¥^ (95% interval)	Optimum Cut-off from ROC	Sensitivity/ Specificity from optimum ROC cut-off
				Median Absorbance (IQR)	Median Absorbance (IQR)		Significance relative to n15		
**Human TDM**	Natural mixture	n15	IgG	3.12 (2.83–3.36)	2.03 (1.3–2.77)	<0.001	0.86 (0.74–0.99)	2.79	80/80
	Natural mixture	n16	IgG(Fc)	2.6 (1.46–2.91)	0.95 (0.63–1.59)	0.001	0.82 (0.68–0.95)	1.41	80/68
	Natural mixture	n17	IgG(g)	2.84 (1.52–3.24)	1.21 (0.64–2.04)	0.003	0.80 (0.64–0.95)	1.96	70/73
	Natural mixture	n18	IgM	2.08 (0.98–2.8)	2.3 (1.15–2.93)	0.616	0.55* (0.34–0.76)	1.79	50/63
	Natural mixture	n19	IgA	0.43 (0.29–1)	0.31 (0.2–0.37)	0.050	0.70 (0.52–0.89)	0.36	70/73
**Bovine TDM**	Natural mixture	n20	IgG	3.02 (2.85–3.24)	1.93 (1.06–2.39)	<0.001	0.88 (0.77–0.99)	2.79	90/85
**α TDM**	4	n21	IgG	2.7 (1.34–3.35)	1.99 (1.45–2.76)	0.233	0.63** (0.39–0.86)	2.98	50/85
**α TMM**	5	n22	IgG	3.11 (2.46–3.59)	1.91 (1.31–2.78)	0.001	0.81 (0.67–0.95)	2.40	80/65
**α TDM**	6	n23	IgG	3.06 (2.88–3.4)	2.19 (1.22–2.89)	0.001	0.82 (0.70–0.94)	2.81	90/68
	6	n3	IgG(Fc)	3.1 (2.29–3.2)	0.96 (0.58–1.67)	<0.001	0.89 (0.80–0.99)	1.70	90/78
**α TMM**	7	n24	IgG	2.94 (2.5–3.29)	1.79 (1.23–3.06)	0.040	0.71* (0.56–0.86)	2.18	90/55
	7	n4	IgG(Fc)	2.31 (1.54–2.84)	1.01 (0.59–2.15)	0.027	0.73* (0.57–0.88)	1.44	80/58
**MeOTDM**	12	n27	IgG	3.17 (2.64–3.35)	1.73 (1.18–2.8)	<0.001	0.85 (0.73–0.97)	2.34	90/68
	12	n28	IgG(Fc)	2.58 (1.97–2.75)	0.73 (0.49–1.32)	<0.001	0.89 (0.76–1.00)	1.78	80/90
	12	n29	IgM	3.07 (2.21–3.14)	2.98 (2.32–3.2)	0.961	0.50*** (0.30–0.69)	3.05	60/63
	12	n30	IgA	1.39 (0.59–1.93)	0.6 (0.34–0.79)	0.017	0.74 (0.57–0.91)	1.19	60/90
**MeOTMM**	13	n31	IgG	1.68 (1.09–3)	0.81 (0.65–1.17)	<0.001	0.84 (0.71–0.97)	1.06	80/70
	13	n32	IgG(Fc)	1.13 (0.65–3.05)	0.68 (0.5–1.15)	0.060	0.69 (0.48–0.90)	1.07	60/73
	13	n33	IgM	1.72 (1.3–1.82)	1.52 (1.1–2.22)	0.982	0.50*** (0.30–0.70)	1.53	70/55
	13	n34	IgA	0.51 (0.27–0.87)	0.27 (0.18–0.39)	0.0724	0.68 (0.49–0.88)	0.36	60/73
**MeOTDM**	14	n35	IgG	3.17 (2.99–3.44)	1.66 (0.85–2.82)	<0.001	0.86 (0.75–0.98)	2.94	80/80
	14	n1	IgG(Fc)	3.48 (2.04–4.08)	0.76 (0.51–1.19)	<0.001	0.94 (0.86–1.00)	1.34	90/83
	14	n1b	IgG(Fc)	0.88 (0.4–2.98)	0.32 (0.27–0.41)	*<0*.*001*	0.86 (0.73–0.98)	0.39	80/67
**MeOTMM**	15	n2	IgG(Fc)	2.96 (1.83–3.74)	0.65 (0.47–1.18)	<0.001	0.92 (0.85–1.00)	1.32	90/85
**t-MeOTDM**	16	n47	IgG(Fc)	1.06 (0.57–3.01)	0.38 (0.29–0.49)	<0.001	0.90 (0.81–1.00)	0.50	90/75
**t-MeOTMM**	17	n36	IgG	2.68 (2.19–3.04)	1.13 (0.77–1.82)	<0.001	0.87 (0.76–0.98)	1.99	80/78
	17	n37	IgG(Fc)	2.08 (1.2–2.91)	0.58 (0.37–1.03)	<0.001	0.88 (0.78–0.99)	1.07	80/78
**t-ketoTDM**	18	n38	IgG	2.07 (0.79–3.34)	0.81 (0.5–1.4)	0.026	0.73* (0.52–0.93)	1.68	60/80
	18	n5	IgG(Fc)	2.79 (1.42–3.08)	0.91 (0.56–1.56)	0.001	0.82 (0.69–0.96)	1.93	70/83
**t-ketoTMM**	19	n6	IgG(Fc)	3.09 (2.16–3.87)	0.97 (0.6–1.76)	<0.001	0.88 (0.78–0.99)	1.79	90/77
**ketoTDM**	20	n39	IgG(Fc)	3.09 (2.16–3.87)	0.97 (0.6–1.76)	<0.001	0.95 (0.89–1.00)	1.88	90/89
	23	nx01	IgG	2.4 (1.35–3.25)	1.12 (0.8–1.69)	0.002	0.82 (0.68–0.96)	1.60	70/73
	24	nx02	IgG	3.09(2.92–3.42)	2.79 (2.45–3.24)	0.057	0.70* (0.54–0.85)	2.82	100/53

Footnotes:

^**#**^ Method: combination of a compound and a secondary antibody

^##^ evaluations were done at a serum dilution of 1:20, except n1b and n47 at 1:80. In each case the secondary antibody was peroxidase conjugated and the binding was measured by addition of o-phenylenediamine and H_2_O_2_ in citrate buffer and the colour reaction was terminated by the addition of acid.

^¥^ ROC analyses are presented in Fig A in S2 File. Standard significance values indicated by stars (* P<0.05, ** P<0.01, *** P<0.001) are for comparisons of ROC results for each method relative to those for method n15. Significance of pairwise differences between AUC values was estimated using the DeLong’s test [67] implemented in the pROC R package. Ab: antibody, TDM: trehalose dimycolate, TMM: trehalose monomycolate ROC: receiver operator characteristics AUC: area under curve PTB: pulmonary tuberculosis IQR: interquartile range.

^¥¥^ MeOTDM: TDM from *cis*-cyclopropane containing methoxy-MA. MeOTMM: TMM from *cis*-cyclopropane methoxy-MA. tMeOTDM: TDM from *trans*-cyclopropane methoxy-MA. tMeOTMM: TMM from t*rans*-cyclopropane methoxy-MA. ketoTDM: TDM from *cis*-cyclopropane ketoMA. t-ketoTDM: TDM from *trans*-cyclopropane ketoMA. t-ketoTMM: TMM from *trans*-cyclopropane ketoMA. α TDM: TDM from α-MA. α TMM: TMM from α-MA.

Most of the single synthetic TDMs and TMMs gave AUC values above 0.8 when IgG or IgG(Fc) secondary antibody was used. Median responses and differences between medians for smear and culture positive PTB and culture negative samples were much lower and not significant for IgM or IgA. This is in line with results when using natural mixtures of TDMs [68, 69].

Individual TDMs and TMMs from each of the common α-, methoxy and keto-classes in *Mtb* gave higher specificities than the natural mixtures. Within the classes, high AUCs were obtained with antigens having a range of different absolute stereochemistries about the different functional groups. Even the model compound **23** with no functionality in the α-chain of the MA (nx01) gave a significant difference between the medians for the two sets. The highest AUC of all (0.95) was obtained with the TDM **17** derived from a *cis*-cyclopropane containing keto-MA (n39).

### Results for the second 50 serum samples

A second set of 50 serum samples was then examined (Table 6; Figs C and D in S2 File). Some 15 of the antigen/antibody combinations giving good culture positive PTB/culture negative distinction with the first 50 samples were included in this study, together with an additional set of antigens covering different structural types of MA. In general, the results with the second set of samples were in agreement with the first set, though in some cases AUC values were rather higher (n4, 0.84 compared to 0.73; n32, 0.86 compared to 0.69) or lower (n2, 0.79 compared to 0.92), probably due to the small sample sets. All of the antigen-secondary antibody combinations again gave a significant difference between smear and culture positive PTB and culture negative sets (p <0.05) (Table 6). With this set of samples, both commercial natural human (n15) and bovine TDM (n20) give slightly higher AUCs using IgG secondary antibody than with the first set, the bovine TDM performing slightly better (AUC 0.90).

**Table 6:** Results of the ELISA with second set of 50 sera [17 culture positive PTB and 33 culture negative patients] using the different combinations of compounds and antibodies.

					TB		No TB						
Type of compound^¥¥^	Compound number	Method^#^	Serum Dilution	2^nd^ Ab used	Smear and culture positive PTB		Culture negative		p value of the comparisons of medians ELISA results from culture positive PTB and culture negative samples	ROC AUC^¥^ (95% interval)	Optimum Cut-off from ROC	Sensitivity/ Specificity from optimum ROC cut-off	Previous AUC as in Table 5
					Median absorbance	IQR	Median absorbance	IQR		Significance relative to n15			
**Human TDM**	Natural mixture	n15	1: 20	IgG	3.11	2.93–3.36	1.51	0.99–2.47	<0.001	0.89 (0.80–0.99)	2.88	82/88	0.86
**Bovine TDM**	Natural mixture	n20	1: 20	IgG	3.2	2.93–3.34	1.66	1.09–2.24	<0.001	0.90 (0.82–0.99)	2.45	88/88	0.88
**α TDM**	6	n3	1: 20	IgG (Fc)	3.14	2.99–3.7	1.1	0.54–2.34	<0.001	0.83 (0.71–0.94)	2.97	76/85	0.89
**α TMM**	7	n4	1: 20	IgG (Fc)	3.21	1.69–3.49	0.89	0.39–1.39	<0.001	0.84 (0.73–0.95)	1.54	82/79	0.73
**tαTDM**	8	n25	1: 20	IgG (Fc)	2.91	2.0–3.37	0.75	0.41–1.61	<0.001	0.87 (0.77–0.97)	1.12	100/67	
**tαTMM**	9	n26	1: 20	IgG (Fc)	3.17	1.73–3.51	0.73	0.39–1.46	<0.001	0.85 (0.74–0.96)	0.99	94/67	
**tαTDM**	10	n45	1: 80	IgG (Fc)	2.04	0.64–2.97	0.46	0.31–0.93	0.010	0.74* (0.58–0.90)	1.11	64/81	
**tαTMM**	11	n46	1: 80	IgG (Fc)	0.74	0.42–2.77	0.29	0.21–0.86	0.020	0.71** (0.55–0.87)	0.28	93/50	
**MeOTDM**	12	n28	1: 20	IgG (Fc)	3.12	1.43–3.51	0.51	0.41–1.52	<0.001	0.86 (0.75–0.97)	2.49	71/94	0.89
**MeOTMM**	13	n32	1: 20	IgG (Fc)	2.91	1.68–3.14	0.74	0.39–1.33	<0.001	0.86 (0.75–0.96)	2.36	65/94	0.69
**MeOTDM**	14	n1	1: 20	IgG (Fc)	3.23	2.24–3.43	0.48	0.29–1.19	<0.001	0.86 (0.75–0.97)	1.25	88/82	0.94
		n1b	1: 80	IgG (Fc)	1.44	0.52–2.34	0.24	0.19–0.42	<0.001	0.85 (0.70–0.99)	0.47	93/81	0.86
**MeOTMM**	15	n2	1: 20	IgG (Fc)	2.84	0.99–3.24	0.6	0.28–1.08	<0.001	0.79 (0.65–0.93)	1.49	65/85	0.92
**tMeOTDM**	16	n47	1: 80	IgG (Fc)	1.95	1.13–3.26	0.37	0.26–0.62	<0.001	0.91 (0.82–0.99)	0.70	93/78	0.90
**tMeOTMM**	17	n37	1: 20	IgG (Fc)	3.15	1.45–3.22	0.9	0.44–2.45	<0.001	0.80 (0.67–0.93)	2.88	71/84	0.88
**tKTDM**	18	n5	1: 20	IgG (Fc)	3.13	2.0–3.52	0.68	0.45–1.28	<0.001	0.89 (0.80–0.98)	1.34	94/79	0.82
**tKTMM**	19	n6	1: 20	IgG (Fc)	3.24	2.42–3.68	0.96	0.52–2.26	<0.001	0.80 (0.67–0.93)	1.18	94/61	0.88
**KTDM**	20	n39	1: 20	IgG (Fc)	3.25	3.0–3.66	0.64	0.29–1.06	<0.001	0.91 (0.83–1.00)	2.59	88/88	0.95
**KTMM**	21	n48	1: 80	IgG (Fc)	2.58	0.65–3.29	0.39	0.22–0.74	<0.001	0.83 (0.69–0.97)	1.50	64/91	
**Alkene TDM**	22	n40	1: 20	IgG (Fc)	3.15	2.41–3.74	0.86	0.51–2.3	<0.001	0.82 (0.71–0.94)	1.40	94/73	
**α MeOTDM**	25	n49	1: 80	IgG (Fc)	2.58	0.97–3.06	0.37	0.25–0.69	<0.001	0.81 (0.67–0.96)	0.60	93/69	

Footnotes:

^**#**^ Method: as for Table 5

^¥ ^ROC analyses are presented in Fig C in S2 File. Standard significance values indicated by stars (* P<0.05, ** P<0.01, *** P<0.001) are for comparisons of ROC results for each method relative to those for method n15. Significance of pairwise differences between AUC values estimated using the DeLong’s test [67] implemented in the pROC R package. Abbreviations: Ab: antibody. ROC: receiver operator characteristics AUC: area under curve PTB: pulmonary tuberculosis IQR: interquartile range.

^¥¥^ Compound description as per Table 5.

A number of additional antigens were also tested with this set (Table 6). Again, all the antigen-secondary antibody combinations responses associated with smear and culture positive PTB rather than culture negative sets. The ROC AUCs ranged from 0.71 to 0.87 (Fig C in S2 File). Within this set, no method was significantly better than using natural human TDM and IgG secondary antibody.

### Combined results for the first 100 serum samples

**Fig 3:**
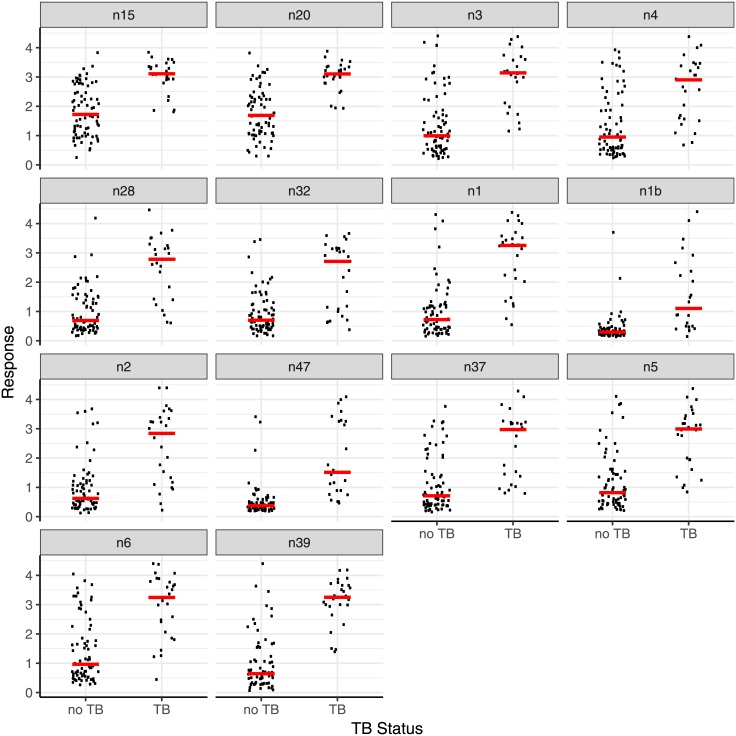
The distribution of responses (absorbances) from the first 100 serum samples from patients with culture positive PTB or culture negative (no TB). Each method n15—n39 is described in Table 7. The bars indicate the medians. In each case the secondary antibody was peroxidase conjugated and the binding was measured by addition of o-phenylenediamine and H_2_O_2_ in citrate buffer and the colour reaction was terminated by the addition of acid. Each individual measurement was carried out in quadruplicate. The bars indicate the medians.

The results for a sub-set of antigens for the whole set of the first 100 samples were combined (Table 7; Fig 3). All antigen-secondary antibody combinations give a significantly higher response with smear and culture positive PTB than culture negative sets. In agreement with what was reported above, one of the antigens, **20** (method n39), gives a rather better discrimination between culture positive PTB and culture negative, with an AUC of 0.94, than the natural human or bovine mixtures (0.88 and 0.89), and antigen **16** (n47), AUC 0.91, performs almost as well (Fig E in S2 File).

**Table 7:** Combined results of the ELISA with the two sets of 50 sera (27 culture positive PTB and 73 culture negative patients) using selected antigen/antibody combinations.

			TB		No TB							
Antigen or compound number	Method#	Secondary antibody	Smear and culture positive PTB		Culture negative		p value^##^	ROC AUC (95% interval)	Optimum Cut-off from ROC	Optimum Sensitivity/ Specificity combination from ROC	Cut-off for 100% sensitivity	SpecificityBased on this cut-off
			Median absorbance	IQR	Median absorbance	IQR		Significance relative to n15				
Human TDM	n15	IgG (whole)	3.11	2.92–3.37	1.72	1.09–2.7	<0.001	0.88 (0.80–0.95)	2.88	78/86	>1.81	51
Bovine TDM	n20	IgG (whole)	3.1	2.86–3.34	1.69	1.08–2.31	<0.001	0.89 (0.83–0.96)	2.45	89/81	>1.93	58
6	n1	IgG (Fc)	3.14	2.47–3.58	0.99	0.54–1.79	<0.001	0.87 (0.80–0.94)	1.26	89/81	>0.54	38
7	n3	IgG (Fc)	2.9	1.59–3.41	0.95	0.53–2.04	<0.001	0.80 (0.71–0.89)	1.72	89/74	>1.15	59
12	n4	IgG (Fc)	2.78	1.63–3.21	0.69	0.43–1.43	<0.001	0.88 (0.80–0.95)	1.38	85/64	>0.62	38
13	n32	IgG (Fc)	2.71	1.04–3.14	0.7	0.47–1.25	<0.001	0.81 (0.71–0.91)	2.14	59/93	>0.37	12
14	n28	IgG (Fc)	3.25	2.07–3.55	0.72	0.4–1.19	<0.001	0.90 (0.83–0.96)	1.25	89/81	>0.61	44
14	n1b	IgG (Fc)	1.1	0.49–2.45	0.3	0.22–0.42	<0.001	0.86 (0.76–0.96)	0.47	79/81	>0.14	1
15	n2	IgG (Fc)	2.84	1.25–3.37	0.62	0.41–1.17	<0.001	0.83 (0.73–0.93)	1.52	70/86	>0.22	3
16	n47	IgG (Fc)	1.51	0.86–3.27	0.37	0.27–0.57	<0.001	0.91 (0.86–0.97)	0.49	96/72	>0.46	62
17	n37	IgG (Fc)	2.97	1.41–3.21	0.71	0.4–1.44	<0.001	0.84 (0.77–0.92)	0.77	100/56	>0.22	49
18	n5	IgG (Fc)	2.99	1.95–3.4	0.82	0.46–1.47	<0.001	0.87 (0.79–0.94)	1.92	78/82	>0.84	53
19	n6	IgG (Fc)	3.24	2.24–3.78	0.97	0.56–1.93	<0.001	0.84 (0.75–0.93)	1.79	85/74	>0.44	14
20	n39	IgG (Fc)	3.25	2.97–3.66	0.64	0.34–1.24	<0.001	0.94 (0.89–0.98)	1.32	100/75	>1.39	77

Footnotes:

^#^ Method: as for Table 5.

^##^ Comparison of medians ELISA results from culture positive PTB and culture negative samples. Abbreviations: PTB: pulmonary tuberculosis; IQR: interquartile range. Ab: antibody, TDM: trehalose dimycolate, TMM: trehalose monomycolate ROC: receiver operator characteristics AUC: area under curve TB: tuberculosis

**Fig 4:**
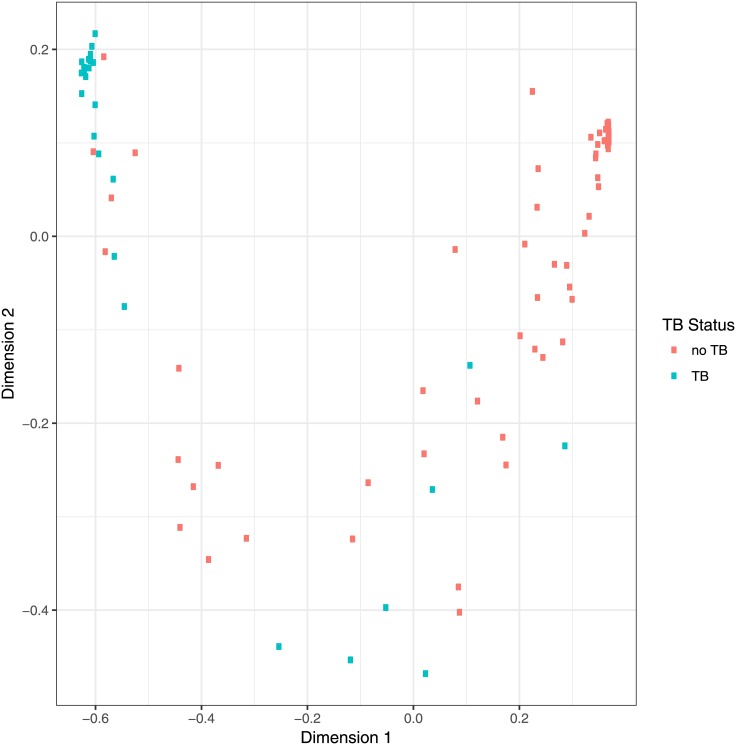
Proximity of individual cases using a Random Forest classifier [63] trained with the absobance for the antigens n15, n20, n3, n28, n32, n1, n39 for the 100 sample data set in R [64]. **Proximities were reduced to 2-dimensions using multidimensional scaling.** Dimensions 1 and 2 can be interpreted as weighted statistical combinations of the absorbances for each serum sample with the antigens. Abbreviations: as for Fig 3.

Analysis of the responses for each individual antigen to each of the sera showed subtle differences in pattern. The results from seven antigen/secondary antibody combinations (n15, n20, n3, n28, n32, n1, n39), were therefore combined using a Random Forest classifier and principal co-ordinate analysis of case proximity, leading to the distribution shown in Fig 4. The two axes represent different combinations of the results with each of the antigens. Two clear groups can be identified, one in the top left hand corner for the bulk of smear and culture positive PTB samples, the other in the top right for culture negative, no TB, samples; in between was a region containing both sets of samples. Using Generalized Boosted Regression Models (GBM) statistics to combine the results from the 7 antigen/antibody combinations provided a prediction of culture positive PTB/culture negative to best match the WHO/TDR diagnosis as a rank order from 0–1, predicting the likelihood that the sample was culture positive PTB (0 very unlikely, 1 very likely). By setting the value of the cut-off for a positive assignment at 0.24, a sensitivity of 100% and a specificity of 89% was achieved. Sensitivity+specificity was maximised with a cut-off of 0.35; the sensitivity was 96% and the specificity 95% (Table 8). A ROC analysis of these combined data gave an AUC of 0.98 (Fig F in S2 File).

**Table 8:** Predictive value of a statistical combination of the responses for methods n15, n20, n3, n28, n32, n1, n39 using the initial 100 samples with GBM statistics.

Cut off used for culture positive PTB using GBM output	>0.24	>0.35
**Sensitivity (%)**	100	96
**Specificity (%)**	89	95
**True positive**	27	26
**False positive**	8	4
**True negative**	65	69
**False negative**	0	1
**Total**	100	100
**PPV**	77	87
**NPV**	100	99

Footnotes: A ROC analysis of the numerical predictions (in range 0 (culture negative) to 1 (smear and culture positive PTB)) for each serum sample combined using GBM statistics gave an AUC of 0.98 (see Fig F in S2 File). NPV: negative predictive value. PPV: positive predictive value.

To set-up a cut-off for the “blind-study” we used the optimal combination of sensitivity and specificity provided by a ROC analysis for the first 100. In addition we evaluated the sensitivity and specificity produced by setting the minimum cut-off for each antigen to provide 100% sensitivity for these samples (Table 7).

### A blind study of 249 serum samples

**Fig 5:**
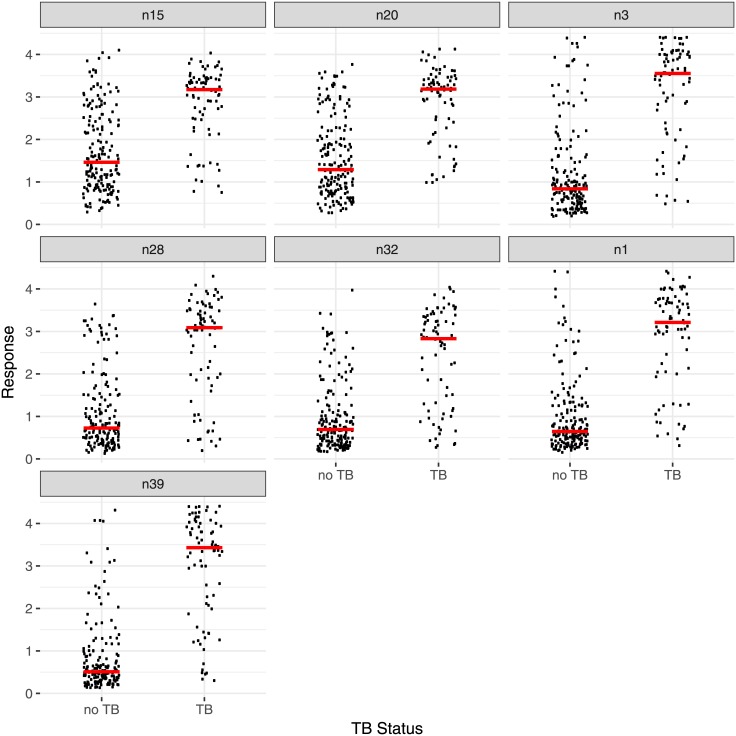
The distribution of responses (absorbances), with medians, in the ELISA assay with the third set of 249 samples using 7 different antigens, natural TDM from MTB (n15), natural bovine TDM (n20) and five synthetic antigens the medians for which are presented in Table 9. The assay was run blind and then un-blinded for analysis compared to cut-off values set for the first 100 samples as in Table 7. Method: as for Fig 3. Abbreviations: TDM: trehalose dimycolate.

Having established that the synthetic antigens do provide responses in ELISA that match well with the diagnosis of smear and culture positive PTB, and that the results from more than one antigen could be combined to enhance the sensitivity and specificity, the method was applied in a blind study of an additional 249 serum samples from presumptive TB patients from the TDR TB Specimen Bank (Table 9; Fig 5, Fig G in S2 File). Patients were enrolled as specified in the Material and Methods section. Note that in a number of cases, the patients are recorded as having either a history of previous TB, and/or of Bacillus Calmette–Guérin (BCG) vaccination, and/or having been affected by a recent or current co-infection (e.g. malaria).

**Table 9:** Results of the blind analysis of the ELISA for 249 sera (75 culture positive PTB and 174 culture negative patients) using selected combinations of compounds and antibodies.

			TB		No TB						
Antigen or compound number	Method^#^	Secondary antibody	Culture positive PTB^$^		Culture negative		P^##^	ROC AUC^¥^ (95% interval)	Sensitivity/ Specificity from optimum cut-off from ROC on first 100 samples	Cut-off based on first 100 samples to give 100% sensitivity	Sensitivity/Specificity with that cut-off
			Median	IQR	Median	IQR					
**Human TDM**	n15	IgG	3.17	2.48–3.43	1.46	0.91–2.46	<0.001	0.80 (0.75–0.86)	64/84	>1.81	83/61
**Bovine TDM**	n20	IgG	3.18	2.41–3.43	1.29	0.79–2.17	<0.001	0.84 (0.79–0.89)	73/79	>1.93	80/71
**6**	n3	IgG (Fc)	3.55	2.25–4.0	0.84	0.51–1.47	<0.001	0.87*** (0.83–0.92)	81/79	>1.15	92/70
**12**	n28	IgG (Fc)	3.09	1.86–3.51	0.73	0.42–1.45	<0.001	0.83 (0.77–0.89)	80/72	>0.61	91/40
**13**	n32	IgG (Fc)	2.83	1.3–3.35	0.69	0.41–1.22	<0.001	0.83 (0.77–0.89)	65/87	>0.37	95/22
**14**	n1	IgG (Fc)	3.21	2.14–3.72	0.65	0.43–1.25	<0.001	0.88*** (0.84–0.93)	84/75	>0.54	96/37
**20**	n39	IgG (Fc)	3.43	2.07–4.0	0.51	0.32–0.92	<0.001	0.90*** (0.86–0.95)	83/84	>1.39	83/85

Footnotes:

^#^ Method: as for Table 5.

^$^ 73 of 75 were also smear positive

^##^ Comparison of medians from culture positive PTB and culture negative samples. The serum dilution used was 1:20.

^¥ ^ROC analyses for these samples after unblinding are presented in Fig G in S2 File. Standard significance values indicated by stars (* P<0.05, ** P<0.01, *** P<0.001) are for comparisons of ROC results for each method relative to those for method n15. Significance of pairwise differences between AUC values was estimated using the DeLong’s test [67] implemented in the pROC R package. Abbreviations: PTB: pulmonary tuberculosis. IQR: interquartile range. Ab: antibody, TDM: trehalose dimycolate, TMM: trehalose monomycolate ROC: receiver operator characteristics AUC: area under curve.

Using cut-off values set prior to the blind study, the sensitivity, specificity and ROC AUC for the natural mixtures of human TDM were 64%, 84% and 0.80 and for the bovine TDM were 73%, 79%, and 0.84 respectively. Three of the synthetic antigens performed better, antigen **20** (n39) giving a sensitivity of 83%, a specificity of 84%, and an AUC of 0.90. For comparison, with the GBM classifier set at the cut-off to achieve 100% sensitivity based on the 100 sample training, a sensitivity of 81%, specificity of 86% and an AUC of 0.83 resulted, while setting a cut-off to achieve maximum sensitivity+specificity gave values of 74%, 87% and 0.81 repectively.

### The results for the complete set of 349 sera

**Fig 6:**
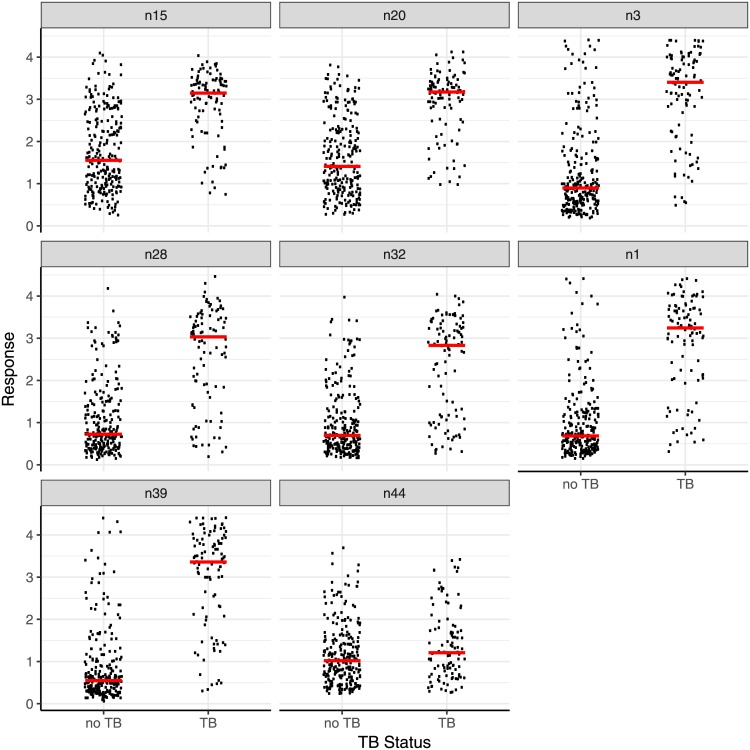
ELISA responses (absorbances) and medians of smear and culture positive PTB and culture negative sets for all 349 samples using 8 different antigens, natural TDM from MTB (n15), natural bovine TDM (n20) and six synthetic antigens with secondary antibodies as described in Table 9. The ROC analysis is given in Fig H in S2 File. Method: as for Fig 3. Abbreviations: TDM: trehalose dimycolate. Mtb: *Mycobacterium tuberculosis*.

Having determined the results for the 249 samples in the above blind study, the data for all 349 samples were then combined (Fig 6, Fig H in S2 File). All the synthetic MA sugar ester-secondary antibody combinations showed a significant difference between smear and culture positive PTB and culture negative sets (p <0.001), with AUCs of 0.84–0.91 (Table 10); in comparison, the free MA **H** (Fig 1B) gave much lower median absorbances and an AUC of just 0.57.

**Table 10:** Median values for absorbances of all 349 serum samples.

		TB		No TB				
Antigen or compound number	Method^#^	Culture positive PTB^$^ Median	IQR	Culture negative Median	IQR	p value	ROC AUC ^¥^ (95% interval)	Sensitivity/ specificity from ROC analysis
**Human TDM**	n15	3.15	2.5–3.4	1.55	0.9–2.5	<0.001	0.82 (0.77–0.87)	68/85
**Bovine TDM**	n20	3.17	2.5–3.4	1.41	0.9–2.2	<0.001	0.85* (0.81–0.89)	78/79
**6**	n3	3.40	2.3–4.0	0.90	0.5–1.6	<0.001	0.87** (0.83–0.91)	84/77
**12**	n28	3.03	1.8–3.5	0.72	0.4–1.4	<0.001	0.84 (0.79–0.89)	75/84
**13**	n32	2.83	1.1–3.3	0.70	0.4–1.2	<0.001	0.83 (0.77–0.88)	63/90
**14**	n1	3.24	2.1–3.7	0.68	0.4–1.2	<0.001	0.89*** (0.85–0.93)	79/89
**20**	n39	3.36	2.3–3.9	0.55	0.3–1.0	<0.001	0.91*** (0.87–0.94)	91/79
**H Fig. b)**	n44	1.21	0.7–1.8	1.0	0.6–1.5	0.031	0.57*** (0.51–0.64)	38/77

**Footnotes**: ^#^ Method: as for Table 5.

^$^ 100 of 102 were also smear positive. For free MA, **H**, n44, IgG(whole).

^¥^ ROC analyses are presented in Fig H in S2 File. Standard significance values indicated by stars (* P<0.05, ** P<0.01, *** P<0.001) are for comparisons of ROC results for each method relative to those for method n15. Significance of pairwise differences between AUC values was estimated using the DeLong’s test [67] implemented in the pROC R package. Abbreviation: IQR, inter-quartile range.

**Fig 7:**
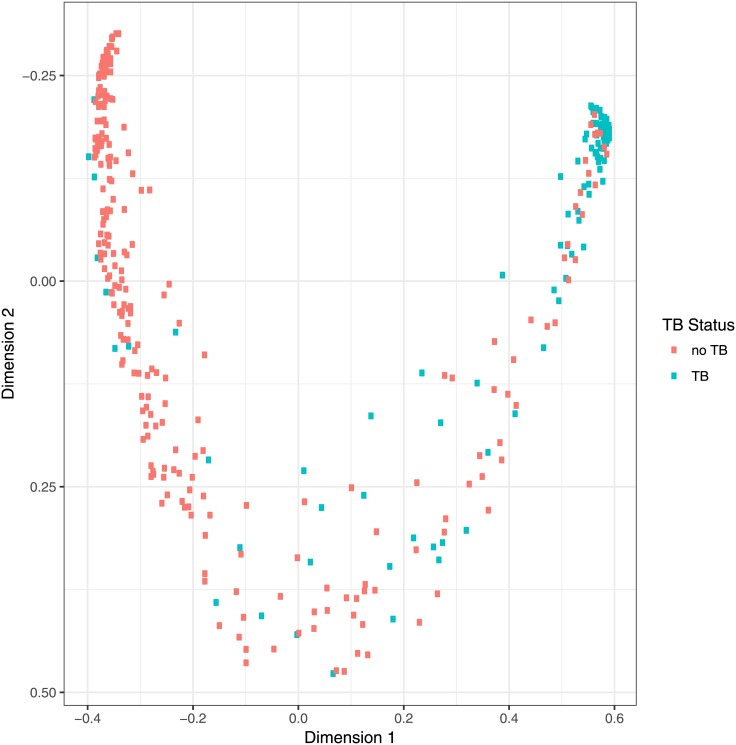
Proximity of individual cases using a Random Forest classifier [59] trained with the absorbances with the 349 serum samples for the antigens n15, n20, n3, n28, n32, n1, n39. The ROC AUC for this analysis based on the principal co-ordinate (x-axis) compared to PTB or no PTB diagnosis was 0.95. AUC: area under curve.

A statistical combination of the results from all the antigen-antibody combinations (except for n44), using a Random Forest classifier, led to a principal co-ordinate analysis that allowed a distinction between culture positive PTB and culture negative (Fig 7) with a ROC AUC of 0.95. For this set of samples 100 of the 102 culture positive PTB samples were both smear and culture positive. Two were smear negative, culture positive; both identified as positives in the above assays.

A more detailed analysis of the individual results showed that, with the cut-off values set by ROC analysis of the initial 100 samples (Table 7), 73 of the 102 samples diagnosed as samples from culture positive PTB patients, were above the cut-off for all antigens used (except method n44) and a further 12 were above the cut-off for six of the seven antigens.

An analysis of the effect of country of origin and other sub-groups is presented in S3 File, but must be considered with caution due to lack of standardisation and quality assurance in data collection.

## Discussion

We set-up a serological test for the diagnosis of TB. Using ELISA with two initial sets of 50 serum samples from patients with presumptive TB [60], we showed that a number of combinations of a defined single synthetic trehalose di- and mono-mycolate antigen and a secondary antibody gave a better distinction than complex natural mixtures of TDM between patients who were diagnosed with smear and culture positive PTB, and those who were negative in culture. The sensitivity and specificity, and the pattern of responses, in each case depended on the detailed structure of the antigen and on the secondary antibody used. By combining the results from a set of individual antigens using GBM statistics, the distinction could be increased to provide a sensitivity and specificity of 100% and 89% respectively and a ROC AUC of 0.98, meeting the criteria of the WHO target product profile [13].

In these initial studies with some 38 combinations of synthetic trehalose ester and secondary antibody, 14 with the complete set of 100 samples, TDMs and TMMs derived from the common classes of MA in *Mtb* (α-, keto- and methoxy-MA), all gave ROC AUCs above 0.85 using IgG or IgG(Fc) secondary antibodies. Among the antigens evaluated, two gave a better discrimination between smear and culture positive PTB and culture negative sets, with AUCs of 0.94 and 0.91, than natural human mixture (0.88).

To validate these results, an additional 249 samples were run blind, using cut-off values set studying the initial 100 samples. An optimal sensitivity and specificity combination of 88% and 83% for a single synthetic antigen/secondary antibody combination **(n5)** was obtained; a number of the synthetic antigen/secondary antibody combinations gave a better distinction than natural TDM. In contrast, the free MA **H** (Fig 1B) (method n44) showed a ROC value of just 0.57.

All of the patients whose sera were included in this study presented with presumptive TB [60] and the majority presented at health facilities in countries with high burdens of TB. They were thus representative of populations in which a rapid TB diagnostic would be deployed. Combining the results for all 349 samples, we show that an ELISA based on the responses of serum antibodies to trehalose esters of single synthetic MA, using IgG(Fc) secondary antibody, was more accurate for TB diagnosis than similar assays using natural mixtures of TDMs.

Moreover, the combination of the results obtained using more than one antigen with the full set of 349 samples again further improved the accuracy of the assay. By using principal co-ordinate analysis, the combination of the results from 7 antigens led to a 2D-plot in which the results for serum samples from smear and culture positive PTB/culture negative (no TB) patients clearly appeared in different areas, with a number in the region between these extremes. This effect of using a set of antigens is in contrast to recent reports of the use of a multiple set of protein antigens, where little improvement was observed [70]. The reason for this is not yet clear, but may reflect increased structural diversity of binding sites between the different lipid antigens compared to protein antigens.

Among the samples tested, no serological response to any antigen was found in 7 samples that were culture positive, bringing the performance of the assay below the optimum WHO target product profile [13]. A number of factors, separately or collectively, could account for these very low responses relative to the culture negative set. Thus, the samples came from a banked set and some may have degraded in storage and use, the disease at the time the serum sample was taken may not have progressed to antibody production, levels of antibodies to lipids may fluctuate during active disease as antibodies to protein antigens are known to do [14–16,53], or there may be differences in response from one country or region or from one population to another. Differences may also be affected by Mtb lineage and strain. Moreover, it is known that antibody responses in patients with TB can be heterogeneous [53]. A very low serological response in some TB patients has been shown in studies using natural TDM [28], and the responses have been associated with the time of clinical onset of symptoms [29].

Co-infection with HIV is reported not to interfere with serodiagnostic TB assays using either natural TDM [30–41] or natural MA mixtures [42–45]. Although the TDR TB Specimen Bank contains sera from HIV-positive patients with symptoms suggestive of PTB, no such sera were included in this initial study; a further study to evaluate the assay with defined synthetic antigens in TB/HIV co-infected serum is on-going. Further work is also required to optimise the combination of synthetic MA based antigens, for example to include the more challenging smear negative samples and to remove the chance of false positives from other mycobacterial infections [71], as well as applying it in a rapid, reliable and robust testing format.

In conclusion, within the set of 349 serum samples selected from different countries, an assay based on the antibody detection against selected individual sugar esters of synthetic MAs had a higher accuracy to distinguish smear and culture positive PTB from smear and culture negative patients, than did the complex natural mixture of natural TDMs, and gave responses that depended on the specific structure of the antigen. By combining the results from more than one synthetic antigen, e.g, including different structural classes of MA, the performance of the assay could be further improved. This is in contrast to some multiplex assays using protein antigens [70,72], perhaps reflecting differences in antibody-lipid interactions from antibody-protein interactions. This study has identified new antigens based on trehalose esters of synthetic MA that are promising candidates for diagnostic tests. They can be produced consistently to a high purity in large quantities and thus have advantages over many other types of antigen with diagnostic potential. We have shown that using them in combination (in a panel) provides better diagnostic performance than using individual antigens, and that the results in ELISA are dependent on the secondary antibody.

Numerous studies have described delay in the diagnosis and treatment of tuberculosis and the key contribution of poor diagnostic capacity at the lower levels of the health services of disease-endemic areas where the majority of TB patients present [73]. The economic burden that this delay places on people seeking diagnosis is enormous. A recent study reported that the total costs of TB to patients in low and middle-income countries was equivalent, on average, to 39% of annual household income [74]. Half the total cost was incurred before treatment. New, more accessible TB diagnostics are required that can bring diagnosis and prompt treatment to the health centres where most patients initially present with their symptoms. Even if a simple, rapid TB test could only diagnose patients with smear positive disease it could be a significant step forward in TB control. It would take TB diagnosis to settings where no sputum smear microscopy currently exists or where smear microscopy labs are overburdened, under-resourced or of poor quality.

## Supporting information

S1 File Average responses for each serum in quadruplicate for each assay, together with standard deviations. (DOCX)

S2 File Analysis of results by sample group. (DOCX)

S3 File Analysis of results by sub-groups (where available). (DOCX)
