# Supplementary material for: New synthetic lipid antigens for rapid serological diagnosis of tuberculosis
Source: PLoS One. 2017 Aug 14;12(8):e0181414. doi: 10.1371/journal.pone.0181414 (PMC5555574; doi:10.1371/journal.pone.0181414)
Supplement: S1 File — (DOCX) [file pone.0181414.s001.docx]

**S1 File**

**Average responses for each serum in quadruplicate for each assay, together with standard deviations**

|  |  |  |  | **n15** |  | **n20** |  | **N3** |  | **N28** |  | **N32** | | |  | **N1** | |  | **N39** |  | | | |  |
| --- | --- | --- | --- | --- | --- | --- | --- | --- | --- | --- | --- | --- | --- | --- | --- | --- | --- | --- | --- | --- | --- | --- | --- | --- |
|  | **Number** | **Set** | **Country** | IgG (whole) |  | IgG (whole) |  | IgG (Fc) |  | IgG (Fc) |  | IgG (Fc) | | |  | IgG (Fc) | |  | IgG (Fc) |  | | | |  |
|  |  |  |  | Ave. | SD | Ave. | SD | Ave. | SD | Ave. | SD | Ave. | SD | | | Ave. | SD | | Ave. | SD | | |  |  |
|  | 1 | 1 | 8 | 2.80 | 0.21 | 2.99 | 0.33 | 1.23 | 0.05 | 0.61 | 0.11 | 0.37 | 0.03 | | | 1.35 | 0.52 | | 1.50 | 0.12 | | |  |  |
|  | 2 | 1 | 8 | 3.14 | 0.71 | 3.23 | 0.39 | 3.11 | 0.31 | 3.14 | 0.33 | 2.71 | 0.11 | | | 4.00 | * | | 4.18 | 0.33 | | |  |  |
|  | 3 | 1 | 8 | 3.28 | 0.13 | 3.88 | 0.28 | 3.08 | 0.37 | 2.79 | 0.10 | 3.16 | 0.25 | | | 4.27 | 0.26 | | 3.45 | 0.24 | | |  |  |
|  | 4 | 1 | 8 | 1.87 | 0.12 | 1.94 | 0.18 | 2.11 | 0.15 | 1.40 | 0.30 | 0.67 | 0.08 | | | 2.02 | 0.44 | | 2.32 | 0.27 | | |  |  |
|  | 5 | 1 | 8 | 3.09 | 0.19 | 3.01 | 0.18 | 3.35 | 0.27 | 2.65 | 0.17 | 0.65 | 0.11 | | | 2.13 | 0.77 | | 3.30 | 0.42 | | |  |  |
|  | 6 | 1 | 11 | 3.38 | 0.46 | 3.24 | 0.15 | 3.21 | 0.25 | 2.61 | 0.24 | 0.63 | 0.05 | | | 3.25 | 0.21 | | 3.17 | 0.21 | | |  |  |
|  | 7 | 1 | 11 | 2.33 | 0.33 | 2.79 | 0.16 | 1.73 | 0.03 | 2.35 | 0.19 | 1.09 | 0.08 | | | 1.19 | 0.06 | | 2.06 | 0.07 | | |  |  |
|  | 8 | 1 | 11 | 3.48 | 0.24 | 2.80 | 0.33 | 3.19 | 0.22 | 2.55 | 0.43 | 3.29 | 0.22 | | | 4.10 | 0.53 | | 2.65 | 0.36 | | |  |  |
|  | 9 | 1 | 11 | 2.93 | 0.30 | 3.04 | 0.52 | 2.83 | 0.10 | 1.84 | 0.30 | 1.18 | 0.06 | | | 3.70 | 0.36 | | 3.51 | 0.10 | | |  |  |
|  | 10 | 1 | 11 | 3.69 | 0.28 | 3.57 | 0.28 | 4.12 | * | 2.78 | 0.10 | 3.47 | | 0.32 | | 4.39 | * | | 3.86 | 0.55 | | |  |  |
|  | 11 | 2 | 8 | 3.50 | 0.64 | 3.28 | 0.44 | 3.57 | 0.67 | 3.16 | 0.28 | 3.14 | | 0.24 | | 3.52 | 0.51 | | 3.57 | 0.33 | | |  |  |
|  | 12 | 2 | 8 | 3.01 | 0.16 | 3.03 | 0.16 | 3.14 | 0.44 | 2.78 | 0.06 | 2.91 | | 0.10 | | 3.23 | 0.68 | | 3.25 | 0.12 | | |  |  |
|  | 13 | 2 | 8 | 3.85 | 0.46 | 3.41 | 0.38 | 2.99 | 0.24 | 3.77 | 0.32 | 3.66 | | 0.61 | | 3.36 | 0.76 | | 3.19 | 0.45 | | |  |  |
|  | 14 | 2 | 12 | 3.36 | 0.23 | 3.37 | 0.17 | 3.00 | 0.17 | 1.43 | 0.27 | 2.17 | | 0.33 | | 2.42 | 0.32 | | 3.09 | 0.22 | | |  |  |
|  | 15 | 2 | 12 | 2.93 | 0.23 | 3.21 | 0.38 | 3.60 | 0.18 | 3.12 | 0.26 | 3.10 | | 0.12 | | 3.49 | 0.22 | | 4.17 | 0.41 | | |  |  |
|  | 16 | 2 | 3 | 3.60 | 0.27 | 3.53 | 0.09 | 3.14 | 0.06 | 4.46 | 0.03 | 3.55 | | 0.22 | | 3.14 | 0.04 | | 3.90 | 0.49 | | |  |  |
|  | 17 | 2 | 11 | 3.33 | 0.34 | 3.69 | 0.14 | 3.70 | 0.49 | 3.67 | 0.19 | 2.39 | | 0.33 | | 3.43 | 0.74 | | 3.66 | 0.76 | | |  |  |
|  | 18 | 2 | 11 | 1.81 | 0.20 | 1.94 | 0.16 | 1.15 | 0.07 | 1.23 | 0.25 | 1.15 | | 0.32 | | 0.76 | 0.04 | | 3.00 | 0.75 | | |  |  |
|  | 19 | 2 | 1 | 3.09 | 0.16 | 3.03 | 0.16 | 4.28 | 0.18 | 2.98 | 0.11 | 3.03 | | 0.13 | | 3.27 | 0.36 | | 2.99 | 0.25 | | |  |  |
|  | 20 | 2 | 1 | 3.61 | 0.74 | 3.10 | 0.34 | 3.13 | 0.39 | 3.51 | 0.67 | 2.88 | | 0.31 | | 3.28 | 0.67 | | 3.30 | 0.34 | | |  |  |
|  | 21 | 2 | 6 | 3.11 | 0.13 | 3.20 | 0.22 | 3.15 | 0.22 | 3.30 | 0.17 | 0.71 | | 0.09 | | 2.24 | 0.16 | | 3.75 | 0.23 | | |  |  |
|  | 22 | 2 | 9 | 3.01 | 0.11 | 2.93 | 0.13 | 4.03 | 0.50 | 2.94 | 0.13 | 3.06 | | 0.23 | | 4.11 | 0.42 | | 3.72 | 0.76 | | |  |  |
|  | 23 | 2 | 9 | 1.86 | 0.10 | 2.52 | 0.08 | 1.76 | 0.15 | 0.88 | 0.10 | 1.00 | | 0.10 | | 1.47 | 0.13 | | 1.47 | 0.30 | | |  |  |
|  | 24 | 2 | 9 | 3.29 | 0.19 | 3.34 | 0.17 | 3.75 | 0.06 | 3.25 | 0.10 | 3.58 | | 0.47 | | 2.91 | 0.23 | | 3.00 | 0.14 | | |  |  |
|  | 25 | 2 | 1 | 2.91 | 0.10 | 2.46 | 0.14 | 1.98 | 0.06 | 1.03 | 0.23 | 1.68 | | 0.19 | | 1.26 | 0.26 | | 2.95 | 0.18 | | |  |  |
|  | 26 | 2 | 1 | 3.20 | 0.31 | 3.34 | 0.33 | 4.38 | 0.01 | 3.51 | 0.52 | 3.15 | | 0.44 | | 3.58 | 0.60 | | 3.66 | 0.70 | | |  |  |
|  | 27 | 2 | 3 | 2.20 | 0.12 | 2.01 | 0.03 | 1.43 | 0.08 | 0.64 | 0.05 | 0.85 | | 0.12 | | 0.55 | 0.02 | | 1.40 | 0.30 | |  |  |  |
|  | 28 | 3 | 8 | 2.12 | 0.19 | 2.94 | 0.30 | 3.29 | 0.35 | 1.60 | 0.23 | 2.91 | | 0.20 | | 3.31 | 0.39 | | 3.47 | 0.62 | |  |  |  |
|  | 29 | 3 | 8 | 0.75 | 0.10 | 1.37 | 0.29 | 1.18 | 0.11 | 0.41 | 0.11 | 0.43 | | 0.05 | | 0.82 | 0.04 | | 1.24 | 0.08 | |  |  |  |
|  | 30 | 3 | 8 | 3.06 | 0.19 | 2.83 | 0.12 | 2.89 | 0.26 | 2.67 | 0.18 | 2.59 | | 0.12 | | 3.05 | 0.57 | | 3.00 | 0.21 | |  |  |  |
|  | 31 | 3 | 8 | 1.01 | 0.24 | 1.91 | 0.36 | 2.23 | 0.09 | 0.31 | 0.05 | 0.48 | | 0.04 | | 2.04 | 0.18 | | 2.58 | 0.18 | |  |  |  |
|  | 32 | 3 | 8 | 3.35 | 0.09 | 3.38 | 0.05 | 3.67 | 0.63 | 3.53 | 0.15 | 3.23 | | 0.09 | | 3.78 | 0.59 | | 3.77 | 0.47 | |  |  |  |
|  | 33 | 3 | 8 | 3.24 | 0.22 | 3.02 | 0.18 | 3.96 | 0.38 | 3.03 | 0.04 | 2.83 | | 0.17 | | 4.03 | 0.32 | | 4.05 | 0.51 | |  |  |  |
|  | 34 | 3 | 8 | 3.45 | 0.08 | 3.71 | 0.51 | 3.61 | 0.62 | 4.30 | 0.32 | 3.24 | | 0.14 | | 3.12 | 0.13 | | 3.60 | 0.70 | |  |  |  |
|  | 35 | 3 | 8 | 3.72 | 0.22 | 3.61 | 0.21 | 3.36 | 0.50 | 3.95 | 0.41 | 3.03 | | 0.19 | | 3.11 | 0.14 | | 3.34 | 0.31 | |  |  |  |
|  | 36 | 3 | 8 | 3.41 | 0.17 | 3.28 | 0.15 | 4.06 | 0.36 | 3.41 | 0.12 | 3.01 | | 0.07 | | 3.99 | 0.37 | | 3.93 | 0.42 | |  |  |  |
|  | 37 | 3 | 8 | 3.43 | 0.33 | 3.29 | 0.15 | 4.09 | 0.01 | 3.48 | 0.23 | 2.66 | | 0.18 | | 3.78 | 0.08 | | 4.25 | 0.22 | |  |  |  |
|  | 38 | 3 | 8 | 1.41 | 0.08 | 1.17 | 0.06 | 1.83 | 0.08 | 0.47 | 0.06 | 0.74 | | 0.07 | | 1.05 | 0.14 | | 1.21 | 0.15 | |  |  |  |
|  | 39 | 3 | 8 | 2.58 | 0.18 | 2.53 | 0.33 | 2.85 | 0.14 | 0.85 | 0.13 | 1.32 | | 0.17 | | 2.00 | 0.52 | | 1.99 | 0.15 | |  |  |  |
|  | 40 | 3 | 8 | 3.74 | 0.30 | 3.43 | 0.19 | 4.09 | 0.44 | 3.87 | 0.45 | 3.35 | | 0.29 | | 3.82 | 0.56 | | 4.39 | * | |  |  |  |
|  | 41 | 3 | 12 | 2.50 | 0.25 | 2.17 | 0.59 | 2.25 | 0.25 | 0.88 | 0.06 | 0.94 | | 0.14 | | 1.30 | 0.22 | | 3.36 | 0.76 | |  |  |  |
|  | 42 | 3 | 12 | 3.50 | 0.23 | 3.55 | 0.10 | 3.89 | 0.41 | 3.89 | 0.40 | 3.22 | | 0.16 | | 3.56 | 0.28 | | 4.15 | 0.47 | |  |  |  |
|  | 43 | 3 | 12 | 3.42 | 0.27 | 3.47 | 0.17 | 3.44 | 0.02 | 3.44 | 0.21 | 3.23 | | 0.10 | | 3.50 | 0.32 | | 4.31 | 0.17 | |  |  |  |
|  | 44 | 3 | 12 | 1.37 | 0.18 | 1.94 | 0.20 | 1.52 | 0.45 | 0.91 | 0.17 | 2.22 | | 0.19 | | 3.20 | 0.63 | | 1.26 | 0.15 | |  |  |  |
|  | 45 | 3 | 12 | 3.50 | 0.11 | 3.99 | 0.54 | 4.39 | 0.01 | 3.70 | 0.32 | 3.53 | | 0.26 | | 3.43 | 0.14 | | 4.40 | 0.01 | |  |  |  |
|  | 46 | 3 | 12 | 0.78 | 0.03 | 1.16 | 0.18 | 0.49 | 0.02 | 0.19 | 0.04 | 0.88 | | 0.12 | | 0.31 | 0.04 | | 0.30 | 0.09 | |  |  |  |
|  | 47 | 3 | 12 | 3.15 | 0.19 | 3.23 | 0.27 | 3.57 | 0.19 | 3.34 | 0.44 | 3.42 | | 0.29 | | 3.68 | 0.52 | | 4.15 | 0.33 | |  |  |  |
|  | 48 | 3 | 12 | 2.20 | 0.26 | 2.49 | 0.29 | 0.55 | 0.03 | 0.47 | 0.06 | 0.68 | | 0.06 | | 0.77 | 0.05 | | 0.48 | 0.09 | |  |  |  |
|  | 49 | 3 | 12 | 2.75 | 0.11 | 2.87 | 0.08 | 4.38 | * | 3.07 | 0.34 | 2.86 | | 0.11 | | 3.65 | 0.42 | | 4.40 | 0.01 | |  |  |  |
|  | 50 | 3 | 12 | 3.04 | 0.11 | 3.14 | 0.19 | 1.54 | 0.09 | 1.04 | 0.06 | 1.06 | | 0.17 | | 0.85 | 0.12 | | 1.16 | 0.13 | |  |  |  |
|  | 51 | 3 | 12 | 3.18 | 0.16 | 3.29 | 0.10 | 3.84 | 0.67 | 3.61 | 0.15 | 3.47 | | 0.13 | | 3.68 | 0.55 | | 3.56 | 0.57 | |  |  |  |
|  | 52 | 3 | 12 | 2.90 | 0.13 | 2.95 | 0.15 | 3.55 | 0.71 | 3.30 | 0.18 | 2.99 | | 0.11 | | 3.10 | 0.02 | | 3.80 | 0.68 | |  |  |  |
|  | 53 | 3 | 3 | 4.04 | 0.39 | 3.86 | 0.59 | 3.73 | 0.50 | 3.80 | 0.31 | 3.53 | | 0.51 | | 3.64 | 0.43 | | 3.45 | 0.35 | |  |  |  |
|  | 54 | 3 | 3 | 3.31 | 0.23 | 3.04 | 0.16 | 2.83 | 0.08 | 2.92 | 0.10 | 0.96 | | 0.13 | | 2.85 | 0.10 | | 2.27 | 0.16 | |  |  |  |
|  | 55 | 3 | 3 | 3.04 | 0.08 | 3.40 | 0.02 | 4.38 | 0.01 | 3.48 | 0.16 | 1.85 | | 0.50 | | 4.37 | * | | 3.92 | 0.21 | |  |  |  |
|  | 56 | 3 | 3 | 2.65 | 0.19 | 1.83 | 0.05 | 1.61 | 0.23 | 2.15 | 0.27 | 3.56 | | 0.40 | | 4.00 | * | | 2.30 | 0.38 | |  |  |  |
|  | 57 | 3 | 3 | 3.65 | 0.23 | 3.41 | 0.25 | 4.40 | 0.05 | 3.93 | 0.48 | 3.65 | | 0.56 | | 4.22 | 0.21 | | 3.80 | 0.38 | |  |  |  |
|  | 58 | 3 | 3 | 1.39 | 0.05 | 1.36 | 0.16 | 0.57 | 0.06 | 0.66 | 0.04 | 0.27 | | 0.01 | | 0.48 | 0.07 | | 0.34 | 0.02 | |  |  |  |
|  | 59 | 3 | 3 | 2.28 | 0.38 | 2.12 | 0.66 | 4.06 | 0.56 | 1.90 | 0.09 | 0.31 | | 0.06 | | 4.04 | 0.36 | | 1.42 | 0.38 | |  |  |  |
|  | 60 | 3 | 3 | 3.56 | 0.15 | 3.47 | 0.17 | 2.90 | 0.09 | 1.92 | 0.29 | 1.00 | | 0.22 | | 2.24 | 0.14 | | 2.12 | 0.08 | |  |  |  |
|  | 61 | 3 | 3 | 3.35 | 0.14 | 3.51 | 0.30 | 2.15 | 0.12 | 1.72 | 0.19 | 3.56 | | 0.22 | | 2.14 | 0.07 | | 1.44 | 0.09 | |  |  |  |
|  | 62 | 3 | 3 | 1.64 | 0.12 | 1.05 | 0.05 | 1.70 | 0.17 | 1.86 | 0.13 | 2.38 | | 0.48 | | 3.65 | 0.36 | | 2.07 | 0.10 | |  |  |  |
|  | 63 | 3 | 3 | 1.38 | 0.21 | 2.27 | 0.09 | 1.36 | 0.27 | 1.03 | 0.11 | 1.49 | | 0.17 | | 1.26 | 0.14 | | 1.04 | 0.16 | |  |  |  |
|  | 64 | 3 | 3 | 3.45 | 0.30 | 3.15 | 0.20 | 4.25 | 0.16 | 3.57 | 0.23 | 3.34 | | 0.18 | | 3.77 | * | | 4.08 | 0.56 | |  |  |  |
|  | 65 | 3 | 10 | 3.33 | 0.27 | 3.23 | 0.26 | 3.49 | 0.79 | 3.32 | 0.10 | 3.57 | | 0.53 | | 3.05 | 0.25 | | 3.25 | 0.41 | |  |  |  |
|  | 66 | 3 | 10 | 2.46 | 0.28 | 3.16 | 0.44 | 3.28 | 0.18 | 1.98 | 0.37 | 1.51 | | 0.17 | | 3.26 | 0.23 | | 3.37 | 0.23 | |  |  |  |
|  | 67 | 3 | 10 | 3.34 | 0.35 | 3.18 | 0.29 | 3.53 | 0.58 | 3.17 | 0.47 | 3.18 | | 0.32 | | 3.21 | 0.30 | | 3.35 | 0.29 | |  |  |  |
|  | 68 | 3 | 10 | 3.03 | 0.17 | 3.10 | 0.30 | 2.13 | 0.13 | 2.00 | 0.19 | 0.81 | | 0.07 | | 2.05 | 0.22 | | 1.87 | 0.12 | |  |  |  |
|  | 69 | 3 | 10 | 3.21 | 0.16 | 3.20 | 0.29 | 3.82 | 0.54 | 3.13 | 0.07 | 3.09 | | 0.23 | | 3.47 | 0.24 | | 3.74 | 0.59 | |  |  |  |
|  | 70 | 3 | 10 | 3.83 | 0.71 | 3.93 | 0.49 | 4.12 | * | 3.85 | 0.54 | 3.94 | | 0.50 | | 4.07 | 0.44 | | 4.27 | 0.20 | |  |  |  |
|  | 71 | 3 | 10 | 1.45 | 0.08 | 1.84 | 0.20 | 1.05 | 0.12 | 0.43 | 0.08 | 0.66 | | 0.03 | | 0.86 | 0.08 | | 0.49 | 0.06 | |  |  |  |
|  | 72 | 3 | 10 | 3.41 | 0.28 | 3.29 | 0.19 | 4.01 | 0.36 | 3.38 | 0.40 | 3.37 | | 0.30 | | 3.67 | 0.66 | | 4.03 | 0.56 | |  |  |  |
|  | 73 | 3 | 10 | 2.73 | 0.12 | 2.91 | 0.31 | 4.10 | * | 2.89 | 0.23 | 2.76 | | 0.24 | | 3.06 | 0.32 | | 4.08 | * | |  |  |  |
|  | 74 | 3 | 11 | 2.77 | 0.14 | 3.33 | 0.72 | 4.24 | 0.37 | 3.10 | 0.07 | 2.69 | | 0.13 | | 3.13 | 0.70 | | 3.34 | | 0.18 | | | |
|  | 75 | 3 | 11 | 3.38 | 0.12 | 3.65 | 0.21 | 3.90 | 0.41 | 3.66 | 0.40 | 3.18 | | 0.22 | | 2.94 | 0.26 | | 3.22 | | 0.09 | | | |
|  | 76 | 3 | 11 | 2.97 | 0.19 | 3.47 | 0.19 | 3.66 | 0.59 | 3.14 | 0.18 | 2.86 | | 0.16 | | 3.88 | 0.64 | | 3.78 | | 0.72 | | | |
|  | 77 | 3 | 11 | 2.99 | 0.17 | 3.11 | 0.15 | 3.04 | 0.08 | 3.03 | 0.16 | 2.92 | | 0.16 | | 3.60 | 0.71 | | 3.49 | | 0.78 | | | |
|  | 78 | 3 | 11 | 3.52 | 0.25 | 3.62 | 0.20 | 3.39 | 0.77 | 3.76 | 0.33 | 2.26 | | 0.18 | | 2.99 | 0.07 | | 3.12 | | 0.29 | | | |
|  | 79 | 3 | 11 | 3.46 | 0.18 | 4.12 | 0.36 | 3.09 | 0.24 | 4.09 | 0.39 | 2.73 | | 0.19 | | 3.30 | 0.35 | | 3.02 | | 0.15 | | | |
|  | 80 | 3 | 11 | 3.13 | 0.28 | 3.53 | 0.12 | 3.93 | 0.58 | 3.27 | 0.10 | 2.44 | | 0.15 | | 3.99 | 0.38 | | 2.55 | | 0.24 | | | |
|  | 81 | 3 | 11 | 2.13 | 0.10 | 2.41 | 0.24 | 2.69 | 0.29 | 2.30 | 0.67 | 1.14 | | 0.03 | | 2.57 | 0.06 | | 1.41 | | 0.06 | | | |
|  | 82 | 3 | 1 | 3.38 | 0.12 | 4.13 | 0.52 | 4.00 | * | 3.99 | 0.22 | 3.41 | | 0.27 | | 4.42 | * | | 4.41 | | 0.00 | | | |
|  | 83 | 3 | 1 | 3.73 | 0.38 | 3.23 | 0.41 | 4.40 | * | 2.98 | 0.27 | 2.83 | | 0.20 | | 3.71 | 0.09 | | 3.92 | | 0.42 | | | |
|  | 84 | 3 | 1 | 2.90 | 0.14 | 2.80 | 0.15 | 4.00 | * | 3.04 | 0.24 | 2.82 | | 0.16 | | 2.86 | 0.61 | | 3.31 | | 0.35 | | | |
|  | 85 | 3 | 1 | 3.34 | 0.24 | 3.35 | 0.38 | 3.50 | * | 3.58 | 0.50 | 3.60 | | 0.35 | | 4.03 | 0.73 | | 4.22 | | 0.37 | | | |
|  | 86 | 3 | 1 | 3.45 | 0.18 | 3.43 | 0.19 | 4.04 | 0.16 | 2.51 | 0.54 | 2.10 | | 0.09 | | 3.00 | 0.24 | | 3.55 | | 0.26 | | | |
|  | 87 | 3 | 1 | 2.24 | 0.15 | 0.98 | 0.05 | 3.86 | 0.50 | 2.65 | 0.53 | 1.19 | | 0.09 | | 2.47 | 0.12 | | 3.88 | | 0.66 | | | |
|  | 88 | 3 | 1 | 2.48 | 0.17 | 1.26 | 0.17 | 1.45 | 0.06 | 1.36 | 0.31 | 1.30 | | 0.17 | | 1.15 | 0.08 | | 1.31 | | 0.13 | | | |
|  | 89 | 3 | 6 | 3.79 | 0.33 | 3.68 | 0.30 | 2.98 | 0.32 | 3.68 | 0.48 | 4.04 | | 0.65 | | 3.01 | 0.25 | | 3.00 | | 0.35 | | | |
|  | 90 | 3 | 6 | 3.22 | 0.16 | 3.30 | 0.13 | 3.41 | 0.14 | 3.73 | 0.15 | 3.86 | | 0.25 | | 4.27 | 0.35 | | 3.80 | | 0.30 | | | |
|  | 91 | 3 | 6 | 1.27 | 0.17 | 1.58 | 0.11 | 0.69 | 0.04 | 0.66 | 0.11 | 0.34 | | 0.04 | | 0.59 | 0.04 | | 0.55 | | 0.03 | | | |
|  | 92 | 3 | 9 | 3.32 | 0.32 | 3.29 | 0.11 | 3.98 | 0.41 | 3.24 | 0.06 | 3.38 | | 0.20 | | 3.72 | 0.06 | | 4.10 | | 0.34 | | | |
|  | 93 | 3 | 9 | 3.27 | 0.11 | 3.20 | 0.23 | 3.63 | 0.24 | 3.25 | 0.22 | 3.45 | | 0.36 | | 3.35 | 0.10 | | 4.25 | | 0.29 | | | |
|  | 94 | 3 | 9 | 3.17 | 0.12 | 2.95 | 0.05 | 4.40 | 0.00 | 3.04 | 0.17 | 2.94 | | 0.04 | | 4.04 | 0.51 | | 4.38 | | 0.00 | | | |
|  | 95 | 3 | 9 | 3.82 | 0.06 | 4.06 | 0.50 | 4.00 | * | 3.47 | 0.30 | 3.79 | | 0.43 | | 4.00 | * | | 4.00 | | * | | | |
|  | 96 | 3 | 9 | 2.73 | 0.25 | 1.54 | 0.17 | 3.93 | 0.66 | 3.20 | 0.17 | 3.56 | | 0.27 | | 3.40 | 0.28 | | 4.27 | | 0.26 | | | |
|  | 97 | 3 | 9 | 1.37 | 0.17 | 0.98 | 0.08 | 2.39 | 0.08 | 2.10 | 0.34 | 1.13 | | 0.21 | | 1.29 | 0.14 | | 1.56 | | 0.22 | | | |
|  | 98 | 3 | 9 | 1.04 | 0.07 | 1.13 | 0.10 | 1.20 | 0.06 | 0.63 | 0.09 | 0.59 | | 0.07 | | 0.71 | 0.03 | | 0.69 | | 0.07 | | | |
|  | 99 | 3 | 9 | 3.71 | 0.25 | 3.64 | 0.27 | 4.26 | 0.20 | 3.20 | 0.29 | 1.86 | | 0.14 | | 1.94 | 0.14 | | 3.50 | | 0.85 | | | |
|  | 100 | 3 | 9 | 3.66 | 0.17 | 3.15 | 0.15 | 4.09 | 0.32 | 3.09 | 0.15 | 3.26 | | 0.14 | | 3.76 | 0.30 | | 4.00 | | * | | | |
|  | 101 | 3 | 9 | 0.90 | 0.06 | 1.43 | 0.30 | 0.67 | 0.10 | 0.45 | 0.07 | 0.36 | | 0.06 | | 0.54 | 0.05 | | 0.45 | | 0.09 | | | |
|  | 102 | 3 | 9 | 3.89 | 0.54 | 3.85 | 0.31 | 3.57 | 0.76 | 3.64 | 0.32 | 4.00 | | 0.36 | | 2.94 | 0.18 | | 3.43 | | 0.17 | | | |
|  | 103 | 1 | 8 | 3.37 | 0.26 | 3.26 | 0.13 | 2.79 | 0.18 | 1.13 | 0.12 | 0.61 | | 0.12 | | 1.25 | 0.02 | | 1.66 | | 0.15 | | | |
|  | 104 | 1 | 8 | 2.03 | 0.12 | 1.37 | 0.11 | 2.99 | 0.08 | 1.28 | 0.22 | 0.99 | | 0.05 | | 2.28 | 0.45 | | 0.77 | | 0.13 | | | |
|  | 105 | 1 | 8 | 3.28 | 0.40 | 3.16 | 0.13 | 3.38 | 0.24 | 2.14 | 0.36 | 0.89 | | 0.09 | | 3.20 | 0.46 | | 2.50 | | 0.24 | | | |
|  | 106 | 1 | 3 | 1.97 | 0.08 | 2.00 | 0.14 | 1.20 | 0.03 | 1.04 | 0.06 | 0.93 | | 0.07 | | 0.71 | 0.18 | | 1.11 | | 0.03 | | | |
|  | 107 | 1 | 3 | 2.23 | 0.10 | 2.23 | 0.08 | 1.31 | 0.08 | 1.15 | 0.12 | 1.22 | | 0.07 | | 1.19 | 0.09 | | 0.77 | | 0.03 | | | |
|  | 108 | 1 | 3 | 1.31 | 0.17 | 1.31 | 0.11 | 1.05 | 0.16 | 0.67 | 0.06 | 0.53 | | 0.04 | | 0.43 | 0.05 | | 0.64 | | 0.07 | | | |
|  | 109 | 1 | 3 | 0.92 | 0.15 | 1.08 | 0.03 | 0.30 | 0.03 | 0.59 | 0.08 | 0.48 | | 0.04 | | 0.67 | 0.03 | | 0.32 | | 0.03 | | | |
|  | 110 | 1 | 3 | 1.72 | 0.06 | 1.34 | 0.05 | 0.82 | 0.02 | 0.70 | 0.03 | 1.66 | | 0.10 | | 2.45 | 0.05 | | 0.34 | | 0.02 | | | |
|  | 111 | 1 | 8 | 2.15 | 0.19 | 2.02 | 0.07 | 1.67 | 0.06 | 0.69 | 0.07 | 0.38 | | 0.03 | | 0.69 | 0.08 | | 0.83 | | 0.11 | | | |
|  | 112 | 1 | 8 | 2.15 | 0.02 | 1.97 | 0.15 | 1.09 | 0.14 | 0.69 | 0.08 | 0.56 | | 0.09 | | 0.47 | 0.02 | | 0.70 | | 0.10 | | | |
|  | 113 | 1 | 8 | 2.25 | 0.13 | 2.36 | 0.14 | 0.96 | 0.22 | 0.49 | 0.07 | 1.06 | | 0.08 | | 0.87 | 0.12 | | 1.54 | | 0.14 | | | |
|  | 114 | 1 | 8 | 2.48 | 0.22 | 2.23 | 0.21 | 1.60 | 0.05 | 0.81 | 0.06 | 0.53 | | 0.04 | | 0.87 | 0.17 | |  | |  | | | |
|  | 115 | 1 | 8 | 2.09 | 0.08 | 2.08 | 0.07 | 0.91 | 0.05 | 0.36 | 0.14 | 0.53 | | 0.05 | | 0.98 | 0.12 | | 1.04 | | 0.03 | | | |
|  | 116 | 1 | 8 | 3.04 | 0.15 | 3.15 | 0.24 | 1.87 | 0.14 | 1.71 | 0.16 | 1.12 | | 0.06 | | 1.60 | 0.10 | | 1.69 | | 0.07 | | | |
|  | 117 | 1 | 8 | 1.12 | 0.03 | 1.02 | 0.08 | 0.82 | 0.05 | 0.80 | 0.06 | 1.60 | | 0.02 | | 1.19 | 0.24 | | 0.63 | | 0.06 | | | |
|  | 118 | 1 | 8 | 1.60 | 0.24 | 1.41 | 0.06 | 0.65 | 0.07 | 1.30 | 0.21 | 0.63 | | 0.10 | | 0.44 | 0.06 | | 0.50 | | 0.07 | | | |
|  | 119 | 1 | 8 | 1.64 | 0.22 | 1.02 | 0.20 | 0.59 | 0.03 | 0.63 | 0.03 | 0.86 | | 0.10 | | 0.26 | 0.03 | |  | |  | | | |
|  | 120 | 1 | 8 | 1.34 | 0.07 | 1.07 | 0.22 | 0.28 | 0.04 | 0.26 | 0.02 | 0.56 | | 0.03 | | 0.73 | 0.12 | | 0.30 | | 0.02 | | | |
|  | 121 | 1 | 10 | 2.86 | 0.14 | 3.02 | 0.26 | 0.54 | 0.05 | 0.47 | 0.04 | 0.44 | | 0.11 | | 0.46 | 0.07 | | 0.48 | | 0.02 | | | |
|  | 122 | 1 | 10 | 2.79 | 0.22 | 2.05 | 0.09 | 0.83 | 0.08 | 1.52 | 0.08 | 1.97 | | 0.26 | | 1.32 | 0.45 | | 0.57 | | 0.04 | | | |
|  | 123 | 1 | 10 | 2.85 | 0.19 | 2.78 | 0.13 | 2.73 | 0.07 | 2.14 | 0.17 | 1.65 | | 0.44 | | 1.80 | 0.20 | | 2.35 | | 0.15 | | | |
|  | 124 | 1 | 10 | 2.35 | 0.06 | 2.31 | 0.12 | 0.96 | 0.06 | 0.88 | 0.03 | 0.88 | | 0.14 | | 0.66 | 0.10 | | 0.70 | | 0.03 | | | |
|  | 125 | 1 | 10 | 0.56 | 0.05 | 0.63 | 0.09 | 0.25 | 0.02 | 0.18 | 0.02 | 0.17 | | 0.05 | |  |  | | 0.14 | | 0.00 | | | |
|  | 126 | 1 | 10 | 0.88 | 0.03 | 0.89 | 0.05 | 0.40 | 0.02 | 0.35 | 0.04 | 0.48 | | 0.02 | | 0.26 | 0.03 | | 0.26 | | 0.04 | | | |
|  | 127 | 1 | 10 | 2.76 | 0.12 | 2.49 | 0.07 | 2.21 | 0.08 | 1.43 | 0.05 | 2.01 | | 0.35 | | 2.07 | 0.26 | | 2.87 | | 0.12 | | | |
|  | 128 | 1 | 10 | 2.95 | 0.12 | 2.68 | 0.11 | 1.68 | 0.05 | 1.57 | 0.09 | 0.70 | | 0.11 | | 1.00 | 0.12 | | 1.06 | | 0.15 | | | |
|  | 129 | 1 | 10 | 1.33 | 0.07 | 1.17 | 0.03 | 0.65 | 0.03 | 0.56 | 0.05 | 0.65 | | 0.06 | | 0.63 | 0.06 | | 0.61 | | 0.08 | | | |
|  | 130 | 1 | 10 | 0.76 | 0.06 | 0.78 | 0.07 | 0.71 | 0.10 | 0.36 | 0.07 | 0.38 | | 0.08 | | 0.43 | 0.05 | | 0.48 | | 0.03 | | | |
|  | 131 | 1 | 10 | 1.16 | 0.06 | 0.99 | 0.01 | 0.50 | 0.02 | 0.54 | 0.05 | 0.81 | | 0.08 | | 0.40 | 0.03 | | 0.50 | | 0.03 | | | |
|  | 132 | 1 | 10 | 0.26 | 0.02 | 0.30 | 0.01 | 0.21 | 0.01 | 0.26 | 0.02 | 0.26 | | 0.03 | | 0.22 | 0.06 | | 0.21 | | 0.06 | | | |
|  | 133 | 1 | 10 | 3.06 | 0.06 | 3.05 | 0.21 | 1.51 | 0.12 | 1.59 | 0.06 | 1.57 | | 0.16 | | 1.12 | 0.11 | | 1.24 | | 0.16 | | | |
|  | 134 | 1 | 10 | 2.95 | 0.12 | 2.94 | 0.21 | 2.93 | 0.34 | 2.04 | 0.05 | 1.66 | | 0.09 | | 1.14 | 0.16 | | 2.61 | | 0.22 | | | |
|  | 135 | 1 | 10 | 2.79 | 0.13 | 2.74 | 0.16 | 3.14 | 0.25 | 2.87 | 0.12 | 2.86 | | 0.35 | | 1.92 | 0.30 | |  | |  | | | |
|  | 136 | 1 | 10 | 1.23 | 0.06 | 0.99 | 0.08 | 0.37 | 0.03 | 0.41 | 0.02 | 0.48 | | 0.01 | | 0.79 | 0.04 | | 0.35 | | 0.03 | | | |
|  | 137 | 1 | 10 | 2.23 | 0.11 | 1.88 | 0.14 | 1.14 | 0.10 | 0.76 | 0.04 | 0.77 | | 0.07 | | 0.76 | 0.15 | | 1.54 | | 0.15 | | | |
|  | 138 | 1 | 10 | 1.49 | 0.16 | 1.45 | 0.08 | 0.47 | 0.01 | 0.50 | 0.03 | 0.63 | | 0.08 | | 0.58 | 0.06 | | 0.51 | | 0.03 | | | |
|  | 139 | 1 | 11 | 1.27 | 0.10 | 0.99 | 0.24 | 0.95 | 0.06 | 1.39 | 0.33 | 1.30 | | 0.08 | | 0.55 | 0.11 | | 0.63 | | 0.09 | | | |
|  | 140 | 1 | 11 | 0.66 | 0.04 | 0.56 | 0.06 | 0.29 | 0.03 | 0.29 | 0.02 | 0.51 | | 0.08 | | 0.45 | 0.08 | |  | |  | | | |
|  | 141 | 1 | 12 | 2.03 | 0.14 | 1.78 | 0.11 | 2.24 | 0.19 | 1.19 | 0.12 | 0.47 | | 0.09 | | 1.18 | 0.12 | | 1.70 | | 0.08 | | | |
|  | 142 | 1 | 12 | 1.64 | 0.18 | 2.15 | 0.08 | 1.45 | 0.08 | 0.43 | 0.05 | 0.43 | | 0.14 | | 0.72 | 0.03 | | 0.47 | | 0.09 | | | |
|  | 143 | 2 | 12 | 1.56 | 0.21 | 1.68 | 0.26 | 1.14 | 0.06 | 0.38 | 0.02 | 0.47 | | 0.08 | | 0.30 | 0.01 | | 0.53 | | 0.05 | | | |
|  | 144 | 2 | 3 | 0.74 | 0.09 | 1.15 | 0.13 | 0.49 | 0.01 | 0.24 | 0.00 | 0.40 | | 0.05 | | 0.27 | 0.06 | | 0.31 | | 0.04 | | | |
|  | 145 | 2 | 10 | 1.51 | 0.09 | 1.60 | 0.12 | 0.93 | 0.11 | 0.40 | 0.05 | 0.53 | | 0.10 | | 0.54 | 0.12 | | 1.06 | | 0.06 | | | |
|  | 146 | 2 | 10 | 0.81 | 0.04 | 1.06 | 0.02 | 0.79 | 0.08 | 0.28 | 0.05 | 0.40 | | 0.02 | | 0.40 | 0.06 | | 0.71 | | 0.07 | | | |
|  | 147 | 2 | 10 | 1.40 | 0.06 | 1.78 | 0.09 | 1.79 | 0.11 | 0.77 | 0.06 | 3.45 | | 0.32 | | 0.91 | 0.11 | | 2.24 | | 0.19 | | | |
|  | 148 | 2 | 10 | 2.91 | 0.18 | 2.92 | 0.21 | 3.16 | 0.11 | 1.27 | 0.23 | 2.17 | | 0.17 | | 1.09 | 0.13 | | 4.40 | | 0.01 | | | |
|  | 149 | 2 | 10 | 1.03 | 0.04 | 1.09 | 0.04 | 0.99 | 0.19 | 0.36 | 0.04 | 0.32 | | 0.02 | | 0.38 | 0.05 | | 0.64 | | 0.05 | | | |
|  | 150 | 2 | 10 | 1.68 | 0.09 | 2.36 | 0.26 | 1.72 | 0.18 | 0.90 | 0.08 | 0.74 | | 0.05 | | 1.12 | 0.14 | | 1.81 | | 0.27 | | | |
|  | 151 | 2 | 10 | 2.45 | 0.24 | 2.23 | 0.46 | 0.42 | 0.08 | 0.17 | 0.03 | 0.24 | | 0.04 | | 0.25 | 0.05 | | 0.30 | | 0.08 | | | |
|  | 152 | 2 | 10 | 2.96 | 0.21 | 2.99 | 0.10 | 2.34 | 0.19 | 1.48 | 0.45 | 1.37 | | 0.14 | | 0.99 | 0.13 | | 0.10 | | 0.05 | | | |
|  | 153 | 2 | 10 | 0.81 | 0.13 | 1.43 | 0.02 | 0.55 | 0.04 | 0.51 | 0.04 | 1.05 | | 0.15 | | 0.35 | 0.05 | | 0.65 | | 0.04 | | | |
|  | 154 | 2 | 10 | 0.96 | 0.09 | 1.42 | 0.11 | 0.52 | 0.03 | 0.30 | 0.04 | 0.87 | | 0.27 | | 0.37 | 0.09 | | 0.69 | | 0.05 | | | |
|  | 155 | 2 | 10 | 0.92 | 0.15 | 1.08 | 0.16 | 1.10 | 0.14 | 0.46 | 0.18 | 1.05 | | 0.07 | | 0.48 | 0.08 | | 1.05 | | 0.15 | | | |
|  | 156 | 2 | 1 | 1.96 | 0.13 | 1.07 | 0.14 | 1.15 | 0.06 | 2.20 | 0.70 | 2.11 | | 0.50 | | 1.23 | 0.07 | | 0.67 | | 0.12 | | | |
|  | 157 | 2 | 1 | 1.93 | 0.10 | 0.80 | 0.06 | 1.44 | 0.09 | 1.42 | 0.38 | 0.94 | | 0.09 | | 1.34 | 0.28 | | 0.59 | | 0.09 | | | |
|  | 158 | 2 | 1 | 1.85 | 0.04 | 1.70 | 0.03 | 1.38 | 0.09 | 1.52 | 0.20 | 2.32 | | 0.58 | | 1.15 | 0.05 | | 0.29 | | 0.07 | | | |
|  | 159 | 2 | 1 | 3.83 | 0.23 | 3.81 | 0.47 | 4.08 | 0.43 | 1.79 | 0.14 | 1.88 | | 0.12 | | 3.82 | 0.60 | | 2.97 | | 0.27 | | | |
|  | 160 | 2 | 1 | 2.20 | 0.21 | 1.66 | 0.12 | 2.43 | 0.07 | 1.29 | 0.17 | 1.05 | | 0.27 | | 1.20 | 0.19 | | 0.89 | | 0.10 | | | |
|  | 161 | 2 | 1 | 2.76 | 0.13 | 1.69 | 0.10 | 2.78 | 0.20 | 2.05 | 0.14 | 0.66 | | 0.05 | | 1.19 | 0.15 | | 1.02 | | 0.25 | | | |
|  | 162 | 2 | 1 | 2.81 | 0.13 | 1.28 | 0.08 | 2.96 | 0.12 | 1.92 | 0.45 | 0.39 | | 0.02 | | 1.73 | 0.12 | | 1.57 | | 0.05 | | | |
|  | 163 | 2 | 1 | 3.12 | 0.16 | 2.30 | 0.12 | 4.19 | 0.18 | 1.82 | 0.22 | 0.81 | | 0.10 | | 1.99 | 0.28 | | 2.13 | | 0.22 | | | |
|  | 164 | 2 | 4 | 2.47 | 0.24 | 1.19 | 0.09 | 0.98 | 0.05 | 0.66 | 0.06 | 1.25 | | 0.11 | | 0.41 | 0.08 | | 0.06 | | 0.02 | | | |
|  | 165 | 2 | 5 | 2.84 | 0.17 | 2.31 | 0.38 | 4.07 | 0.43 | 2.94 | 0.17 | 1.65 | | 0.25 | | 4.09 | 0.60 | | 3.63 | | 0.21 | | | |
|  | 166 | 2 | 5 | 0.92 | 0.03 | 0.30 | 0.01 | 0.39 | 0.04 | 0.42 | 0.06 | 0.18 | | 0.01 | | 0.15 | 0.03 | | 0.17 | | 0.05 | | | |
|  | 167 | 2 | 5 | 0.99 | 0.06 | 0.50 | 0.01 | 0.32 | 0.02 | 0.36 | 0.03 | 0.26 | | 0.01 | | 0.18 | 0.06 | | 0.16 | | 0.02 | | | |
|  | 168 | 2 | 5 | 1.09 | 0.16 | 0.43 | 0.05 | 0.54 | 0.04 | 0.41 | 0.04 | 0.21 | | 0.01 | | 0.28 | 0.02 | | 0.33 | | 0.07 | | | |
|  | 169 | 2 | 5 | 1.35 | 0.24 | 2.15 | 0.29 | 1.27 | 0.09 | 1.58 | 0.28 | 1.33 | | 0.14 | | 0.74 | 0.09 | | 0.58 | | 0.12 | | | |
|  | 170 | 2 | 6 | 0.51 | 0.11 | 0.60 | 0.03 | 0.32 | 0.01 | 0.44 | 0.10 | 0.35 | | 0.03 | | 0.23 | 0.03 | | 0.15 | | 0.03 | | | |
|  | 171 | 2 | 6 | 2.70 | 0.58 | 3.38 | 0.46 | 4.40 | * | 4.18 | 0.37 | 3.39 | | 0.23 | | 4.31 | 0.21 | | 3.46 | | 0.25 | | | |
|  | 172 | 2 | 6 | 1.25 | 0.20 | 2.44 | 0.13 | 0.64 | 0.09 | 0.47 | 0.04 | 0.32 | | 0.02 | | 0.23 | 0.04 | | 0.28 | | 0.06 | | | |
|  | 173 | 2 | 6 | 0.98 | 0.10 | 1.36 | 0.11 | 0.58 | 0.02 | 0.47 | 0.04 | 0.39 | | 0.02 | | 0.38 | 0.02 | | 0.28 | | 0.10 | | | |
|  | 174 | 2 | 6 | 0.99 | 0.05 | 2.24 | 0.09 | 0.45 | 0.05 | 0.51 | 0.03 | 0.41 | | 0.03 | | 0.25 | 0.03 | | 0.28 | | 0.07 | | | |
|  | 175 | 2 | 6 | 1.05 | 0.12 | 1.71 | 0.11 | 0.54 | 0.03 | 0.44 | 0.05 | 0.60 | | 0.11 | | 0.29 | 0.07 | | 0.64 | | 0.16 | | | |
|  | 176 | 3 | 12 | 3.32 | 0.11 | 2.94 | 0.13 | 2.21 | 0.10 | 2.01 | 0.14 | 0.35 | | 0.03 | | 1.82 | 0.12 | | 1.86 | | 0.29 | | | |
|  | 177 | 3 | 3 | 1.22 | 0.07 | 1.53 | 0.22 | 0.75 | 0.04 | 0.67 | 0.06 | 0.66 | | 0.13 | | 0.64 | 0.07 | | 0.38 | | 0.08 | | | |
|  | 178 | 3 | 3 | 1.51 | 0.18 | 1.30 | 0.08 | 0.66 | 0.01 | 0.64 | 0.10 | 1.57 | | 0.35 | | 0.79 | 0.07 | | 0.63 | | 0.16 | | | |
|  | 179 | 3 | 3 | 2.78 | 0.08 | 2.74 | 0.10 | 0.72 | 0.05 | 0.57 | 0.06 | 0.90 | | 0.09 | | 0.55 | 0.06 | | 0.43 | | 0.06 | | | |
|  | 180 | 3 | 3 | 1.34 | 0.05 | 1.70 | 0.16 | 0.93 | 0.11 | 1.13 | 0.09 | 1.36 | | 0.14 | | 1.14 | 0.11 | | 0.60 | | 0.04 | | | |
|  | 181 | 3 | 10 | 1.94 | 0.20 | 2.93 | 0.08 | 0.87 | 0.03 | 0.73 | 0.04 | 0.56 | | 0.03 | | 0.83 | 0.12 | | 1.01 | | 0.16 | | | |
|  | 182 | 3 | 10 | 1.17 | 0.08 | 1.77 | 0.15 | 0.76 | 0.12 | 0.48 | 0.09 | 0.61 | | 0.08 | | 0.81 | 0.05 | | 0.67 | | 0.15 | | | |
|  | 183 | 3 | 10 | 3.12 | 0.11 | 2.95 | 0.16 | 4.38 | * | 3.05 | 0.26 | 2.90 | | 0.16 | | 4.00 | * | | 4.07 | | * | | | |
|  | 184 | 3 | 10 | 1.17 | 0.15 | 1.60 | 0.08 | 0.67 | 0.01 | 0.31 | 0.02 | 0.69 | | 0.19 | | 0.53 | 0.06 | | 0.49 | | 0.03 | | | |
|  | 185 | 3 | 10 | 0.89 | 0.07 | 1.26 | 0.07 | 0.36 | 0.04 | 0.24 | 0.05 | 0.29 | | 0.03 | | 0.35 | 0.02 | | 0.21 | | 0.09 | | | |
|  | 186 | 3 | 10 | 2.23 | 0.33 | 2.55 | 0.24 | 1.99 | 0.35 | 0.75 | 0.11 | 0.83 | | 0.06 | | 1.36 | 0.08 | | 1.30 | | 0.49 | | | |
|  | 187 | 3 | 10 | 0.37 | 0.04 | 0.50 | 0.05 | 0.34 | 0.02 | 0.15 | 0.01 | 0.74 | | 0.04 | | 0.31 | 0.01 | | 0.20 | | 0.08 | | | |
|  | 188 | 3 | 10 | 0.83 | 0.05 | 1.16 | 0.09 | 0.49 | 0.04 | 0.21 | 0.03 | 0.33 | | 0.10 | | 0.37 | 0.02 | | 0.28 | | 0.13 | | | |
|  | 189 | 3 | 10 | 1.74 | 0.12 | 2.68 | 0.09 | 1.30 | 0.06 | 0.39 | 0.04 | 0.54 | | 0.04 | | 0.98 | 0.07 | | 0.77 | | 0.06 | | | |
|  | 190 | 3 | 10 | 0.74 | 0.06 | 1.00 | 0.12 | 0.35 | 0.07 | 0.23 | 0.03 | 0.47 | | 0.03 | | 0.50 | 0.03 | | 0.27 | | 0.08 | | | |
|  | 191 | 3 | 10 | 1.58 | 0.15 | 1.65 | 0.38 | 1.30 | 0.09 | 0.42 | 0.08 | 0.71 | | 0.10 | | 1.27 | 0.09 | | 0.51 | | 0.02 | | | |
|  | 192 | 3 | 10 | 0.81 | 0.06 | 0.85 | 0.02 | 0.83 | 0.03 | 0.31 | 0.04 | 0.54 | | 0.05 | | 0.57 | 0.06 | | 0.42 | | 0.03 | | | |
|  | 193 | 3 | 10 | 2.50 | 0.10 | 3.20 | 0.13 | 3.74 | 0.52 | 1.75 | 0.28 | 0.88 | | 0.06 | | 3.20 | 0.64 | | 3.31 | | 0.62 | | | |
|  | 194 | 3 | 10 | 1.13 | 0.08 | 1.40 | 0.08 | 0.78 | 0.06 | 0.53 | 0.02 | 0.91 | | 0.16 | | 0.60 | 0.06 | | 0.46 | | 0.04 | | | |
|  | 195 | 3 | 10 | 2.17 | 0.27 | 2.28 | 0.15 | 1.48 | 0.06 | 1.11 | 0.09 | 0.75 | | 0.06 | | 1.50 | 0.20 | | 1.01 | | 0.29 | | | |
|  | 196 | 3 | 10 | 0.72 | 0.02 | 0.80 | 0.10 | 0.29 | 0.01 | 0.27 | 0.02 | 0.42 | | 0.05 | | 0.38 | 0.03 | | 0.19 | | 0.02 | | | |
|  | 197 | 3 | 10 | 0.44 | 0.06 | 0.52 | 0.03 | 0.37 | 0.03 | 0.30 | 0.03 | 0.55 | | 0.07 | | 0.57 | 0.04 | | 0.20 | | 0.01 | | | |
|  | 198 | 3 | 10 | 0.63 | 0.04 | 0.79 | 0.06 | 0.27 | 0.02 | 0.21 | 0.03 | 0.57 | | 0.03 | | 0.28 | 0.02 | | 0.14 | | 0.01 | | | |
|  | 199 | 3 | 10 | 0.94 | 0.41 | 0.88 | 0.09 | 0.28 | 0.01 | 0.36 | 0.06 | 0.24 | | 0.05 | | 0.57 | 0.07 | | 0.30 | | 0.04 | | | |
|  | 200 | 3 | 10 | 0.93 | 0.13 | 1.09 | 0.09 | 0.33 | 0.03 | 0.36 | 0.08 | 0.33 | | 0.06 | | 0.42 | 0.06 | | 0.21 | | 0.02 | | | |
|  | 201 | 3 | 10 | 1.40 | 0.10 | 1.13 | 0.03 | 0.76 | 0.05 | 0.73 | 0.01 | 0.87 | | 0.11 | | 1.43 | 0.10 | | 0.45 | | 0.02 | | | |
|  | 202 | 3 | 10 | 0.49 | 0.06 | 0.63 | 0.18 | 0.26 | 0.02 | 0.21 | 0.02 | 0.25 | | 0.03 | | 0.21 | 0.03 | | 0.15 | | 0.02 | | | |
|  | 203 | 3 | 10 | 1.27 | 0.09 | 0.92 | 0.04 | 0.53 | 0.04 | 0.44 | 0.02 | 0.35 | | 0.02 | | 0.40 | 0.05 | | 0.39 | | 0.16 | | | |
|  | 204 | 3 | 10 | 0.41 | 0.03 | 0.53 | 0.02 | 0.31 | 0.05 | 0.28 | 0.03 | 0.26 | | 0.04 | | 0.24 | 0.01 | | 0.23 | | 0.01 | | | |
|  | 205 | 3 | 10 | 0.81 | 0.02 | 0.86 | 0.06 | 0.47 | 0.04 | 0.25 | 0.03 | 0.34 | | 0.02 | | 0.48 | 0.03 | | 0.28 | | 0.02 | | | |
|  | 206 | 3 | 10 | 1.18 | 0.00 | 1.45 | 0.18 | 0.68 | 0.03 | 0.34 | 0.04 | 0.30 | | 0.04 | | 0.52 | 0.04 | | 0.49 | | 0.01 | | | |
|  | 207 | 3 | 10 | 0.78 | 0.16 | 1.48 | 0.13 | 0.62 | 0.06 | 0.34 | 0.08 | 0.94 | | 0.37 | | 0.46 | 0.07 | | 0.38 | | 0.09 | | | |
|  | 208 | 3 | 10 | 0.39 | 0.09 | 0.91 | 0.08 | 0.23 | 0.02 | 0.18 | 0.02 | 0.48 | | 0.15 | | 0.20 | 0.02 | | 0.21 | | 0.01 | | | |
|  | 209 | 3 | 10 | 1.55 | 0.29 | 1.88 | 0.08 | 0.19 | 0.03 | 0.75 | 0.20 | 2.71 | | 0.19 | | 0.88 | 0.15 | | 0.52 | | 0.02 | | | |
|  | 210 | 3 | 10 | 0.54 | 0.07 | 1.39 | 0.21 | 0.95 | 0.08 | 0.44 | 0.05 | 0.80 | | 0.20 | | 0.35 | 0.02 | | 0.27 | | 0.03 | | | |
|  | 211 | 3 | 10 | 1.00 | 0.16 | 1.20 | 0.13 | 0.80 | 0.16 | 0.47 | 0.02 | 1.26 | | 0.17 | | 0.59 | 0.03 | | 0.48 | | 0.08 | | | |
|  | 212 | 3 | 10 | 3.09 | 0.11 | 2.97 | 0.12 | 4.18 | 0.18 | 3.01 | 0.16 | 2.99 | | 0.10 | | 4.40 | * | | 4.07 | | 0.35 | | | |
|  | 213 | 3 | 10 | 1.04 | 0.07 | 0.95 | 0.12 | 0.78 | 0.04 | 0.62 | 0.15 | 1.14 | | 0.06 | | 0.56 | 0.05 | | 0.62 | | 0.10 | | | |
|  | 214 | 3 | 10 | 0.45 | 0.04 | 0.56 | 0.06 | 0.40 | 0.03 | 0.21 | 0.06 | 0.38 | | 0.09 | | 0.27 | 0.01 | | 0.27 | | 0.02 | | | |
|  | 215 | 3 | 10 | 0.92 | 0.07 | 1.12 | 0.18 | 1.00 | 0.11 | 0.31 | 0.09 | 0.42 | | 0.11 | | 0.87 | 0.10 | | 0.58 | | 0.04 | | | |
|  | 216 | 3 | 10 | 2.91 | 0.08 | 2.29 | 0.24 | 1.77 | 0.19 | 1.66 | 0.26 | 3.07 | | 0.43 | | 1.77 | 0.08 | | 0.93 | | 0.02 | | | |
|  | 217 | 3 | 10 | 0.97 | 0.05 | 0.99 | 0.10 | 0.63 | 0.03 | 0.35 | 0.04 | 1.51 | | 0.32 | | 1.23 | 0.13 | | 0.37 | | 0.02 | | | |
|  | 218 | 3 | 10 | 0.52 | 0.03 | 1.07 | 0.13 | 0.49 | 0.05 | 0.20 | 0.02 | 1.06 | | 0.21 | | 0.35 | 0.05 | | 0.37 | | 0.05 | | | |
|  | 219 | 3 | 10 | 2.15 | 0.39 | 2.02 | 0.15 | 1.46 | 0.18 | 1.52 | 0.28 | 1.66 | | 0.28 | | 1.25 | 0.09 | | 0.89 | | 0.06 | | | |
|  | 220 | 3 | 10 | 0.46 | 0.06 | 0.57 | 0.05 | 0.27 | 0.03 | 0.26 | 0.06 | 0.43 | | 0.16 | | 0.23 | 0.01 | | 0.22 | | 0.05 | | | |
|  | 221 | 3 | 10 | 1.81 | 0.29 | 1.41 | 0.11 | 1.09 | 0.32 | 0.95 | 0.24 | 1.01 | | 0.20 | | 0.92 | 0.09 | | 0.78 | | 0.10 | | | |
|  | 222 | 3 | 10 | 3.13 | 0.36 | 2.82 | 0.20 | 1.78 | 0.19 | 1.50 | 0.42 | 1.61 | | 0.10 | | 1.95 | 0.10 | | 1.01 | | 0.05 | | | |
|  | 223 | 3 | 10 | 1.17 | 0.10 | 0.99 | 0.10 | 1.04 | 0.11 | 0.52 | 0.08 | 2.39 | | 0.19 | | 0.70 | 0.05 | | 0.61 | | 0.15 | | | |
|  | 224 | 3 | 10 | 1.62 | 0.21 | 1.80 | 0.10 | 0.88 | 0.03 | 0.83 | 0.29 | 1.60 | | 0.03 | | 0.92 | 0.11 | | 0.53 | | 0.02 | | | |
|  | 225 | 3 | 10 | 0.29 | 0.05 | 0.38 | 0.03 | 0.20 | 0.03 | 0.13 | 0.03 | 0.20 | | 0.03 | | 0.21 | 0.02 | | 0.17 | | 0.02 | | | |
|  | 226 | 3 | 10 | 0.64 | 0.04 | 0.95 | 0.06 | 0.31 | 0.04 | 0.32 | 0.06 | 0.74 | | 0.14 | | 0.30 | 0.03 | | 0.20 | | 0.03 | | | |
|  | 227 | 3 | 10 | 2.16 | 0.05 | 1.81 | 0.12 | 2.92 | 0.64 | 2.04 | 0.29 | 2.56 | | 0.32 | | 3.05 | 0.22 | | 2.34 | | 0.21 | | | |
|  | 228 | 3 | 1 | 1.11 | 0.04 | 1.26 | 0.07 | 0.71 | 0.03 | 0.61 | 0.06 | 1.10 | | 0.13 | | 0.49 | 0.06 | | 0.39 | | 0.09 | | | |
|  | 229 | 3 | 1 | 1.30 | 0.12 | 1.37 | 0.12 | 1.01 | 0.07 | 1.51 | 0.07 | 2.04 | | 0.08 | | 0.80 | 0.11 | | 0.71 | | 0.05 | | | |
|  | 230 | 3 | 1 | 3.06 | 0.19 | 3.25 | 0.21 | 4.41 | * | 3.37 | 0.20 | 3.41 | | 0.67 | | 4.42 | * | | 4.06 | | 0.41 | | | |
|  | 231 | 3 | 1 | 1.40 | 0.02 | 1.76 | 0.13 | 0.92 | 0.05 | 0.79 | 0.28 | 1.92 | | 0.22 | | 0.90 | 0.03 | | 0.64 | | 0.16 | | | |
|  | 232 | 3 | 1 | 2.32 | 0.15 | 3.05 | 0.08 | 1.61 | 0.11 | 1.88 | 0.08 | 2.61 | | 0.09 | | 1.06 | 0.14 | | 1.25 | | 0.11 | | | |
|  | 233 | 3 | 1 | 2.59 | 0.14 | 2.20 | 0.22 | 0.97 | 0.09 | 1.07 | 0.23 | 0.88 | | 0.17 | | 0.67 | 0.09 | | 0.60 | | 0.13 | | | |
|  | 234 | 3 | 1 | 0.78 | 0.12 | 0.88 | 0.03 | 0.37 | 0.01 | 0.43 | 0.05 | 1.02 | | 0.20 | | 0.34 | 0.08 | | 0.21 | | 0.05 | | | |
|  | 235 | 3 | 1 | 3.23 | 0.38 | 3.30 | 0.06 | 1.56 | 0.14 | 2.94 | 0.18 | 2.22 | | 0.24 | | 1.66 | 0.12 | | 1.54 | | 0.08 | | | |
|  | 236 | 3 | 1 | 1.24 | 0.07 | 1.74 | 0.05 | 0.59 | 0.12 | 0.84 | 0.06 | 0.50 | | 0.05 | | 0.46 | 0.08 | | 0.36 | | 0.04 | | | |
|  | 237 | 3 | 1 | 3.24 | 0.18 | 1.90 | 0.12 | 2.07 | 0.12 | 1.10 | 0.15 | 2.49 | | 0.37 | | 1.33 | 0.18 | | 1.17 | | 0.22 | | | |
|  | 238 | 3 | 1 | 1.84 | 0.10 | 1.87 | 0.11 | 0.87 | 0.03 | 1.50 | 0.13 | 2.11 | | 0.07 | | 0.97 | 0.05 | | 0.51 | | 0.07 | | | |
|  | 239 | 3 | 1 | 1.15 | 0.07 | 0.77 | 0.05 | 0.90 | 0.10 | 1.54 | 0.11 | 2.06 | | 0.32 | | 0.80 | 0.11 | | 0.55 | | 0.07 | | | |
|  | 240 | 3 | 1 | 3.93 | 0.42 | 3.56 | 0.21 | 2.79 | 0.10 | 2.82 | 0.20 | 2.98 | | 0.03 | | 2.66 | 0.05 | | 2.53 | | 0.24 | | | |
|  | 241 | 3 | 1 | 0.85 | 0.08 | 0.53 | 0.02 | 0.59 | 0.08 | 0.55 | 0.14 | 1.11 | | 0.16 | | 0.52 | 0.09 | | 0.33 | | 0.07 | | | |
|  | 242 | 3 | 1 | 3.30 | 0.21 | 3.19 | 0.17 | 3.40 | 0.28 | 2.82 | 0.41 | 2.59 | | 0.75 | | 3.23 | 0.43 | | 1.66 | | 0.18 | | | |
|  | 243 | 3 | 1 | 0.78 | 0.03 | 0.37 | 0.01 | 0.58 | 0.04 | 0.68 | 0.14 | 2.80 | | 0.53 | | 0.53 | 0.03 | | 0.19 | | 0.00 | | | |
|  | 244 | 3 | 1 | 2.42 | 0.06 | 1.76 | 0.06 | 1.60 | 0.19 | 0.80 | 0.02 | 1.87 | | 0.76 | | 1.67 | 0.15 | | 0.62 | | 0.04 | | | |
|  | 245 | 3 | 1 | 0.80 | 0.07 | 0.51 | 0.06 | 1.00 | 0.11 | 0.83 | 0.23 | 2.96 | | 0.30 | | 1.32 | 0.19 | | 0.55 | | 0.04 | | | |
|  | 246 | 3 | 1 | 0.94 | 0.08 | 0.63 | 0.03 | 0.61 | 0.04 | 0.50 | 0.17 | 0.95 | | 0.10 | | 0.54 | 0.09 | | 0.30 | | 0.02 | | | |
|  | 247 | 3 | 1 | 1.59 | 0.16 | 0.63 | 0.05 | 0.63 | 0.08 | 0.38 | 0.05 | 0.40 | | 0.06 | | 0.48 | 0.14 | | 0.31 | | 0.13 | | | |
|  | 248 | 3 | 1 | 2.82 | 0.12 | 1.86 | 0.10 | 0.62 | 0.04 | 0.50 | 0.05 | 0.70 | | 0.06 | | 0.58 | 0.04 | | 0.36 | | 0.06 | | | |
|  | 249 | 3 | 1 | 1.62 | 0.01 | 0.97 | 0.07 | 0.98 | 0.14 | 0.44 | 0.06 | 0.75 | | 0.12 | | 0.74 | 0.11 | | 0.45 | | 0.12 | | | |
|  | 250 | 3 | 1 | 3.18 | 0.08 | 2.85 | 0.18 | 2.55 | 0.08 | 2.49 | 0.38 | 2.26 | | 0.23 | | 2.49 | 0.12 | | 1.29 | | 0.08 | | | |
|  | 251 | 3 | 1 | 1.47 | 0.11 | 1.07 | 0.08 | 0.72 | 0.14 | 0.62 | 0.07 | 0.61 | | 0.06 | | 0.62 | 0.05 | | 0.52 | | 0.12 | | | |
|  | 252 | 3 | 1 | 1.65 | 0.06 | 1.65 | 0.20 | 1.15 | 0.30 | 0.72 | 0.13 | 2.96 | | 0.14 | | 1.25 | 0.08 | | 0.46 | | 0.06 | | | |
|  | 253 | 3 | 1 | 0.93 | 0.15 | 1.07 | 0.06 | 0.63 | 0.09 | 0.47 | 0.01 | 0.79 | | 0.11 | | 0.54 | 0.12 | | 0.39 | | 0.05 | | | |
|  | 254 | 3 | 1 | 0.86 | 0.05 | 0.74 | 0.07 | 0.37 | 0.02 | 0.37 | 0.05 | 0.51 | | 0.09 | | 0.30 | 0.02 | | 0.25 | | 0.06 | | | |
|  | 255 | 3 | 1 | 2.85 | 0.06 | 3.02 | 0.25 | 1.58 | 0.13 | 1.19 | 0.08 | 1.24 | | 0.23 | | 1.24 | 0.32 | | 1.32 | | 0.83 | | | |
|  | 256 | 3 | 1 | 1.66 | 0.16 | 1.41 | 0.08 | 0.93 | 0.09 | 0.64 | 0.10 | 1.98 | | 0.41 | | 0.73 | 0.09 | | 0.81 | | 0.12 | | | |
|  | 257 | 3 | 1 | 0.57 | 0.04 | 0.72 | 0.06 | 0.35 | 0.04 | 0.30 | 0.04 | 0.38 | | 0.04 | | 0.32 | 0.09 | | 0.27 | | 0.01 | | | |
|  | 258 | 3 | 1 | 1.02 | 0.05 | 0.90 | 0.07 | 0.59 | 0.05 | 0.51 | 0.07 | 1.03 | | 0.22 | | 0.50 | 0.04 | | 0.41 | | 0.11 | | | |
|  | 259 | 3 | 1 | 0.81 | 0.09 | 0.77 | 0.14 | 0.49 | 0.08 | 0.41 | 0.09 | 0.97 | | 0.07 | | 0.33 | 0.08 | | 0.23 | | 0.05 | | | |
|  | 260 | 3 | 1 | 2.30 | 0.24 | 1.98 | 0.17 | 0.73 | 0.09 | 0.58 | 0.05 | 1.27 | | 0.23 | | 0.54 | 0.07 | | 0.50 | | 0.05 | | | |
|  | 261 | 3 | 1 | 3.64 | 0.16 | 3.28 | 0.12 | 3.03 | 0.17 | 3.25 | 0.17 | 3.43 | | 0.23 | | 3.32 | 0.39 | | 2.34 | | 0.19 | | | |
|  | 262 | 3 | 1 | 1.55 | 0.13 | 1.51 | 0.15 | 1.09 | 0.08 | 0.81 | 0.19 | 1.09 | | 0.38 | | 1.26 | 0.09 | | 0.74 | | 0.07 | | | |
|  | 263 | 3 | 1 | 0.86 | 0.09 | 0.74 | 0.03 | 0.62 | 0.03 | 0.42 | 0.04 | 0.82 | | 0.33 | | 0.65 | 0.02 | | 0.32 | | 0.02 | | | |
|  | 264 | 3 | 1 | 0.70 | 0.04 | 0.81 | 0.03 | 0.86 | 0.04 | 0.39 | 0.03 | 0.52 | | 0.10 | | 0.70 | 0.05 | | 0.55 | | 0.08 | | | |
|  | 265 | 3 | 1 | 2.44 | 0.19 | 2.20 | 0.40 | 1.65 | 0.11 | 1.17 | 0.24 | 0.85 | | 0.30 | | 1.34 | 0.12 | | 0.92 | | 0.08 | | | |
|  | 266 | 3 | 1 | 1.15 | 0.09 | 1.18 | 0.08 | 0.99 | 0.22 | 0.78 | 0.13 | 0.65 | | 0.18 | | 0.55 | 0.03 | | 0.49 | | 0.12 | | | |
|  | 267 | 3 | 1 | 3.22 | 0.39 | 2.02 | 0.17 | 2.17 | 0.29 | 2.35 | 0.18 | 0.82 | | 0.18 | | 1.46 | 0.06 | | 2.37 | | 0.11 | | | |
|  | 268 | 3 | 1 | 2.59 | 0.09 | 1.07 | 0.03 | 2.86 | 0.25 | 2.23 | 0.16 | 1.01 | | 0.12 | | 2.00 | 0.11 | | 1.66 | | 0.25 | | | |
|  | 269 | 3 | 1 | 2.72 | 0.18 | 0.84 | 0.03 | 1.24 | 0.19 | 1.14 | 0.23 | 0.55 | | 0.07 | | 0.88 | 0.06 | | 0.54 | | 0.06 | | | |
|  | 270 | 3 | 1 | 2.14 | 0.16 | 0.95 | 0.14 | 1.52 | 0.07 | 1.28 | 0.20 | 0.76 | | 0.19 | | 1.15 | 0.11 | | 0.87 | | 0.10 | | | |
|  | 271 | 3 | 1 | 2.81 | 0.05 | 0.92 | 0.03 | 3.09 | 0.30 | 1.98 | 0.34 | 0.61 | | 0.02 | | 2.79 | 0.18 | | 2.11 | | 0.66 | | | |
|  | 272 | 3 | 1 | 1.24 | 0.08 | 0.54 | 0.06 | 1.23 | 0.05 | 0.79 | 0.09 | 0.68 | | 0.05 | | 0.63 | 0.05 | | 0.39 | | 0.08 | | | |
|  | 273 | 3 | 1 | 2.47 | 0.07 | 1.12 | 0.12 | 1.22 | 0.08 | 1.23 | 0.05 | 0.75 | | 0.03 | | 0.90 | 0.16 | | 0.65 | | 0.05 | | | |
|  | 274 | 3 | 1 | 2.81 | 0.11 | 1.62 | 0.11 | 1.84 | 0.24 | 1.70 | 0.07 | 1.44 | | 0.15 | | 1.27 | 0.15 | | 1.39 | | 0.43 | | | |
|  | 275 | 3 | 1 | 2.31 | 0.10 | 0.72 | 0.02 | 0.64 | 0.05 | 1.12 | 0.10 | 1.87 | | 0.20 | | 0.42 | 0.02 | | 0.41 | | 0.08 | | | |
|  | 276 | 3 | 1 | 3.05 | 0.33 | 2.24 | 0.07 | 2.29 | 0.13 | 3.14 | 0.20 | 0.88 | | 0.06 | | 1.43 | 0.17 | | 1.11 | | 0.05 | | | |
|  | 277 | 3 | 1 | 2.90 | 0.15 | 1.89 | 0.15 | 2.20 | 0.17 | 2.92 | 0.04 | 0.62 | | 0.08 | | 1.34 | 0.20 | | 1.16 | | 0.07 | | | |
|  | 278 | 3 | 1 | 2.22 | 0.09 | 0.71 | 0.05 | 1.18 | 0.15 | 1.39 | 0.33 | 0.58 | | 0.05 | | 0.77 | 0.03 | | 0.63 | | 0.12 | | | |
|  | 279 | 3 | 1 | 3.59 | 0.16 | 2.65 | 0.05 | 3.14 | 0.17 | 2.99 | 0.30 | 1.15 | | 0.12 | | 2.13 | 0.05 | | 1.64 | | 0.14 | | | |
|  | 280 | 3 | 1 | 1.58 | 0.13 | 0.63 | 0.02 | 0.74 | 0.11 | 1.45 | 0.24 | 1.52 | | 0.08 | | 0.61 | 0.09 | | 0.48 | | 0.07 | | | |
|  | 281 | 3 | 1 | 3.85 | 0.28 | 3.45 | 0.20 | 3.94 | 0.59 | 3.65 | 0.41 | 1.10 | | 0.16 | | 3.81 | * | | 4.32 | | 0.18 | | | |
|  | 282 | 3 | 4 | 4.10 | 0.14 | 3.50 | 0.18 | 3.88 | 0.48 | 2.90 | 0.36 | 2.18 | | 0.29 | | 2.76 | 0.05 | | 3.09 | | 0.26 | | | |
|  | 283 | 3 | 4 | 1.97 | 0.22 | 1.40 | 0.08 | 3.29 | 0.39 | 0.85 | 0.16 | 0.54 | | 0.07 | | 2.48 | 0.39 | | 2.87 | | 0.33 | | | |
|  | 284 | 3 | 4 | 3.04 | 0.18 | 1.27 | 0.04 | 1.27 | 0.07 | 1.18 | 0.08 | 0.62 | | 0.07 | | 1.03 | 0.05 | | 0.90 | | 0.11 | | | |
|  | 284 | 3 | 4 | 2.46 | 0.27 | 1.89 | 0.12 | 1.13 | 0.09 | 0.84 | 0.12 | 0.49 | | 0.02 | | 0.71 | 0.11 | | 0.63 | | 0.17 | | | |
|  | 286 | 3 | 5 | 3.58 | 0.14 | 3.01 | 0.20 | 3.27 | 0.45 | 3.08 | 0.18 | 0.71 | | 0.05 | | 1.72 | 0.09 | | 2.03 | | 0.24 | | | |
|  | 287 | 3 | 5 | 1.70 | 0.08 | 0.61 | 0.23 | 0.26 | 0.01 | 0.48 | 0.02 | 0.33 | | 0.03 | | 0.32 | 0.06 | | 0.23 | | 0.07 | | | |
|  | 288 | 3 | 5 | 2.34 | 0.07 | 0.74 | 0.18 | 0.70 | 0.05 | 0.77 | 0.05 | 0.30 | | 0.03 | | 0.63 | 0.08 | | 0.53 | | 0.08 | | | |
|  | 289 | 3 | 5 | 0.84 | 0.04 | 0.28 | 0.01 | 0.35 | 0.03 | 0.43 | 0.08 | 0.23 | | 0.02 | | 0.35 | 0.02 | | 0.24 | | 0.03 | | | |
|  | 290 | 3 | 5 | 3.49 | 0.28 | 1.28 | 0.19 | 2.85 | 0.24 | 3.25 | 0.33 | 0.67 | | 0.04 | | 2.30 | 0.30 | | 2.48 | | 0.19 | | | |
|  | 291 | 3 | 5 | 0.58 | 0.02 | 0.28 | 0.04 | 0.29 | 0.01 | 0.27 | 0.06 | 0.19 | | 0.01 | | 0.32 | 0.02 | | 0.14 | | 0.04 | | | |
|  | 292 | 3 | 5 | 2.51 | 0.22 | 0.78 | 0.19 | 1.00 | 0.10 | 1.52 | 0.23 | 0.26 | | 0.02 | | 1.09 | 0.14 | | 0.87 | | 0.01 | | | |
|  | 293 | 3 | 5 | 1.19 | 0.13 | 0.46 | 0.10 | 0.34 | 0.03 | 0.30 | 0.03 | 0.32 | | 0.03 | | 0.29 | 0.03 | | 0.24 | | 0.03 | | | |
|  | 294 | 3 | 5 | 1.54 | 0.13 | 0.45 | 0.05 | 0.36 | 0.03 | 0.69 | 0.04 | 0.18 | | 0.01 | | 0.34 | 0.03 | | 0.25 | | 0.06 | | | |
|  | 295 | 3 | 5 | 1.90 | 0.08 | 0.48 | 0.05 | 0.45 | 0.05 | 0.69 | 0.10 | 0.22 | | 0.01 | | 0.44 | 0.05 | | 0.37 | | 0.09 | | | |
|  | 296 | 3 | 5 | 1.39 | 0.07 | 0.48 | 0.04 | 0.37 | 0.02 | 0.60 | 0.09 | 0.33 | | 0.01 | | 0.26 | 0.01 | | 0.27 | | 0.01 | | | |
|  | 297 | 3 | 5 | 1.61 | 0.08 | 0.50 | 0.04 | 0.50 | 0.04 | 0.55 | 0.08 | 0.21 | | 0.02 | | 0.33 | 0.04 | | 0.32 | | 0.06 | | | |
|  | 298 | 3 | 5 | 0.87 | 0.03 | 0.38 | 0.03 | 0.30 | 0.03 | 0.25 | 0.03 | 0.18 | | 0.01 | | 0.25 | 0.02 | | 0.23 | | 0.05 | | | |
|  | 299 | 3 | 5 | 1.64 | 0.12 | 0.55 | 0.05 | 0.65 | 0.05 | 0.76 | 0.03 | 0.31 | | 0.01 | | 0.38 | 0.08 | | 0.40 | | 0.03 | | | |
|  | 300 | 3 | 5 | 3.91 | 0.54 | 3.24 | 0.17 | 4.26 | 0.22 | 3.37 | 0.31 | 1.03 | | 0.15 | | 3.01 | 1.10 | | 3.13 | | 0.25 | | | |
|  | 301 | 3 | 5 | 4.05 | 0.34 | 2.85 | 0.12 | 3.75 | 0.49 | 3.14 | 0.11 | 0.86 | | 0.08 | | 2.28 | 0.17 | | 2.26 | | 0.20 | | | |
|  | 302 | 3 | 5 | 2.85 | 0.13 | 0.91 | 0.06 | 1.34 | 0.14 | 1.00 | 0.18 | 0.48 | | 0.03 | | 0.58 | 0.06 | | 0.51 | | 0.08 | | | |
|  | 303 | 3 | 5 | 2.12 | 0.14 | 0.61 | 0.03 | 0.99 | 0.14 | 0.67 | 0.05 | 0.39 | | 0.05 | | 0.61 | 0.05 | | 0.71 | | 0.06 | | | |
|  | 304 | 3 | 5 | 1.12 | 0.24 | 0.46 | 0.04 | 0.27 | 0.03 | 0.23 | 0.01 | 0.17 | | 0.03 | | 0.25 | 0.06 | | 0.22 | | 0.06 | | | |
|  | 305 | 3 | 5 | 0.89 | 0.09 | 0.41 | 0.04 | 0.42 | 0.02 | 0.38 | 0.06 | 0.20 | | 0.01 | | 0.37 | 0.08 | | 0.23 | | 0.05 | | | |
|  | 306 | 3 | 5 | 0.59 | 0.13 | 0.32 | 0.03 | 0.37 | 0.03 | 1.06 | 0.13 | 0.27 | | 0.06 | | 0.28 | 0.03 | | 0.32 | | 0.07 | | | |
|  | 307 | 3 | 5 | 0.75 | 0.16 | 1.04 | 0.10 | 0.51 | 0.03 | 2.74 | 0.07 | 0.66 | | 0.05 | | 0.53 | 0.12 | | 0.64 | | 0.09 | | | |
|  | 308 | 3 | 5 | 0.52 | 0.08 | 0.32 | 0.06 | 0.35 | 0.03 | 0.38 | 0.03 | 0.39 | | 0.05 | | 0.28 | 0.04 | | 0.15 | | 0.03 | | | |
|  | 309 | 3 | 5 | 2.34 | 0.20 | 0.67 | 0.08 | 0.96 | 0.07 | 1.27 | 0.20 | 0.30 | | 0.03 | | 0.43 | 0.02 | | 1.30 | | 0.12 | | | |
|  | 310 | 3 | 5 | 0.42 | 0.10 | 0.82 | 0.11 | 0.33 | 0.01 | 0.49 | 0.07 | 0.33 | | 0.05 | | 0.23 | 0.03 | | 0.14 | | 0.02 | | | |
|  | 311 | 3 | 5 | 2.94 | 0.19 | 2.95 | 0.13 | 3.85 | 0.48 | 3.07 | 0.14 | 2.94 | | 0.10 | | 3.59 | 0.61 | | 3.41 | | 0.70 | | | |
|  | 312 | 3 | 5 | 1.28 | 0.19 | 1.05 | 0.13 | 0.79 | 0.10 | 1.01 | 0.18 | 0.42 | | 0.11 | | 0.73 | 0.08 | | 0.57 | | 0.10 | | | |
|  | 313 | 3 | 5 | 0.72 | 0.14 | 1.02 | 0.03 | 0.73 | 0.05 | 0.78 | 0.07 | 0.44 | | 0.10 | | 0.77 | 0.04 | | 0.66 | | 0.08 | | | |
|  | 314 | 3 | 5 | 0.32 | 0.08 | 0.53 | 0.04 | 0.53 | 0.05 | 0.50 | 0.13 | 0.27 | | 0.03 | | 0.45 | 0.06 | | 0.65 | | 0.09 | | | |
|  | 315 | 3 | 6 | 1.54 | 0.37 | 2.21 | 0.16 | 0.70 | 0.06 | 0.83 | 0.10 | 0.49 | | 0.05 | | 0.56 | 0.02 | | 0.42 | | 0.05 | | | |
|  | 316 | 3 | 6 | 1.45 | 0.23 | 1.63 | 0.08 | 0.83 | 0.04 | 1.00 | 0.30 | 0.88 | | 0.10 | | 0.70 | 0.08 | | 0.43 | | 0.03 | | | |
|  | 317 | 3 | 6 | 1.31 | 0.33 | 1.83 | 0.24 | 0.75 | 0.05 | 0.90 | 0.19 | 0.88 | | 0.14 | | 0.59 | 0.02 | | 0.53 | | 0.03 | | | |
|  | 318 | 3 | 6 | 0.87 | 0.12 | 0.89 | 0.11 | 0.21 | 0.03 | 0.50 | 0.05 | 0.31 | | 0.02 | | 0.15 | 0.02 | | 0.13 | | 0.02 | | | |
|  | 319 | 3 | 6 | 2.25 | 0.52 | 2.53 | 0.22 | 0.84 | 0.03 | 1.07 | 0.19 | 0.45 | | 0.04 | | 0.68 | 0.06 | | 0.44 | | 0.06 | | | |
|  | 320 | 3 | 6 | 1.77 | 0.17 | 2.27 | 0.11 | 1.06 | 0.17 | 0.84 | 0.11 | 0.66 | | 0.09 | | 0.95 | 0.07 | | 0.85 | | 0.22 | | | |
|  | 321 | 3 | 6 | 1.94 | 0.33 | 3.32 | 0.32 | 0.97 | 0.04 | 1.98 | 0.40 | 0.52 | | 0.04 | | 0.81 | 0.10 | | 0.43 | | 0.07 | | | |
|  | 322 | 3 | 6 | 0.91 | 0.29 | 1.82 | 0.43 | 0.92 | 0.06 | 1.63 | 0.42 | 0.54 | | 0.09 | | 0.61 | 0.01 | | 0.47 | | 0.04 | | | |
|  | 323 | 3 | 6 | 1.33 | 0.10 | 2.02 | 0.20 | 0.94 | 0.05 | 0.67 | 0.11 | 0.48 | | 0.05 | | 0.70 | 0.05 | | 0.48 | | 0.07 | | | |
|  | 324 | 3 | 6 | 0.95 | 0.08 | 0.72 | 0.06 | 0.42 | 0.02 | 0.40 | 0.02 | 0.32 | | 0.04 | | 0.42 | 0.06 | | 0.41 | | 0.07 | | | |
|  | 325 | 3 | 6 | 1.10 | 0.02 | 0.76 | 0.02 | 0.38 | 0.03 | 0.42 | 0.08 | 0.66 | | 0.09 | | 0.34 | 0.04 | | 0.34 | | 0.01 | | | |
|  | 326 | 3 | 6 | 1.21 | 0.10 | 1.87 | 0.14 | 0.83 | 0.06 | 0.62 | 0.11 | 0.63 | | 0.10 | | 0.61 | 0.06 | | 0.51 | | 0.06 | | | |
|  | 327 | 3 | 6 | 2.77 | 0.18 | 3.59 | 0.45 | 1.62 | 0.02 | 1.73 | 0.11 | 1.84 | | 0.35 | | 1.30 | 0.13 | | 0.99 | | 0.24 | | | |
|  | 328 | 3 | 6 | 1.29 | 0.24 | 1.94 | 0.13 | 0.36 | 0.02 | 0.38 | 0.04 | 0.26 | | 0.05 | | 0.33 | 0.08 | | 0.30 | | 0.08 | | | |
|  | 329 | 3 | 6 | 0.93 | 0.14 | 1.42 | 0.07 | 0.38 | 0.06 | 0.33 | 0.02 | 0.43 | | 0.07 | | 0.30 | 0.04 | | 0.25 | | 0.04 | | | |
|  | 330 | 3 | 6 | 0.70 | 0.08 | 0.97 | 0.07 | 0.56 | 0.02 | 0.66 | 0.04 | 0.49 | | 0.11 | | 0.37 | 0.03 | | 0.38 | | 0.08 | | | |
|  | 331 | 3 | 6 | 3.28 | 0.08 | 3.77 | 0.08 | 2.82 | 0.23 | 3.15 | 0.14 | 2.29 | | 0.18 | | 2.45 | 0.37 | | 1.72 | | 0.86 | | | |
|  | 332 | 3 | 6 | 2.82 | 0.07 | 3.49 | 0.28 | 1.78 | 0.04 | 2.03 | 0.22 | 1.85 | | 0.16 | | 1.57 | 0.08 | | 1.06 | | 0.08 | | | |
|  | 333 | 3 | 6 | 0.81 | 0.23 | 1.12 | 0.06 | 0.37 | 0.04 | 0.33 | 0.03 | 0.25 | | 0.03 | | 0.29 | 0.04 | | 0.21 | | 0.03 | | | |
|  | 334 | 3 | 6 | 1.30 | 0.18 | 2.07 | 0.18 | 0.80 | 0.11 | 0.89 | 0.17 | 1.95 | | 0.18 | | 0.51 | 0.02 | | 0.51 | | 0.09 | | | |
|  | 335 | 3 | 6 | 1.14 | 0.24 | 2.32 | 0.09 | 0.65 | 0.08 | 0.66 | 0.14 | 0.34 | | 0.03 | | 0.50 | 0.08 | | 0.61 | | 0.06 | | | |
|  | 336 | 3 | 6 | 2.54 | 0.14 | 2.93 | 0.11 | 2.08 | 0.12 | 1.41 | 0.17 | 0.77 | | 0.17 | | 1.58 | 0.12 | | 1.18 | | 0.03 | | | |
|  | 337 | 3 | 6 | 1.05 | 0.18 | 1.10 | 0.21 | 0.59 | 0.04 | 0.50 | 0.03 | 0.27 | | 0.05 | | 0.46 | 0.04 | | 0.39 | | 0.03 | | | |
|  | 338 | 3 | 6 | 1.18 | 0.06 | 1.39 | 0.07 | 0.93 | 0.04 | 0.61 | 0.11 | 0.34 | | 0.05 | | 0.74 | 0.05 | | 0.59 | | 0.03 | | | |
|  | 339 | 3 | 6 | 1.73 | 0.15 | 2.08 | 0.13 | 0.86 | 0.05 | 1.00 | 0.16 | 0.31 | | 0.06 | | 0.71 | 0.04 | | 0.68 | | 0.04 | | | |
|  | 340 | 3 | 6 | 2.42 | 0.42 | 3.59 | 0.45 | 1.14 | 0.10 | 0.95 | 0.10 | 0.72 | | 0.09 | | 0.73 | 0.04 | | 0.56 | | 0.04 | | | |
|  | 341 | 3 | 6 | 0.88 | 0.03 | 1.60 | 0.21 | 1.04 | 0.14 | 0.69 | 0.05 | 1.28 | | 0.20 | | 0.57 | 0.04 | | 0.47 | | 0.03 | | | |
|  | 342 | 3 | 6 | 0.54 | 0.07 | 0.67 | 0.07 | 0.52 | 0.05 | 0.37 | 0.05 | 0.37 | | 0.10 | | 0.40 | 0.01 | | 0.31 | | 0.03 | | | |
|  | 343 | 3 | 6 | 1.80 | 0.13 | 2.61 | 0.36 | 1.02 | 0.04 | 1.81 | 0.10 | 0.48 | | 0.01 | | 0.75 | 0.07 | | 0.77 | | 0.08 | | | |
|  | 344 | 3 | 6 | 1.25 | 0.07 | 1.41 | 0.03 | 0.52 | 0.03 | 0.52 | 0.11 | 0.32 | | 0.04 | | 0.47 | 0.02 | | 0.35 | | 0.02 | | | |
|  | 345 | 3 | 9 | 2.95 | 0.18 | 2.89 | 0.15 | 4.26 | 0.22 | 2.91 | 0.23 | 2.93 | | 0.13 | | 3.16 | 0.16 | | 2.64 | | 0.83 | | | |
|  | 346 | 3 | 9 | 2.65 | 0.15 | 3.23 | 0.15 | 1.99 | 0.14 | 1.42 | 0.31 | 0.88 | | 0.12 | | 1.15 | 0.02 | | 1.00 | | 0.18 | | | |
|  | 347 | 3 | 9 | 1.10 | 0.06 | 1.22 | 0.15 | 0.90 | 0.02 | 0.67 | 0.25 | 0.49 | | 0.05 | | 0.62 | 0.08 | | 0.63 | | 0.08 | | | |
|  | 348 | 3 | 9 | 2.77 | 0.17 | 2.78 | 0.18 | 2.13 | 0.09 | 2.34 | 0.06 | 0.94 | | 0.13 | | 1.50 | 0.32 | | 1.52 | | 0.17 | | | |
|  | 349 | 3 | 9 | 3.62 | 0.05 | 3.48 | 0.21 | 3.73 | 0.59 | 3.30 | 0.13 | 3.97 | | 0.51 | | 2.77 | 0.21 | | 3.09 | | 0.21 | | | |

Footnotes: Set: 1, First 50; 2, second fifty; 3, blind 249. Red in columns 1-3 are culture positive pulmonary TB; blue are culture negative not pulmonary TB. Country codes: 1, Gambia; 3, South Africa; 4, Brazil; 5, Canada; 6, Spain; 8, Bangladesh; 9, Vietnam; 10, Colombia; 11, Kenya; 12, Peru.

*In these cases, the result of the assay for three of four measurements was above the cut-off value for the instrument, and all were visually very strong responses. The figure given is that which the instrument did record.
