# Supplementary material for: New synthetic lipid antigens for rapid serological diagnosis of tuberculosis
Source: PLoS One. 2017 Aug 14;12(8):e0181414. doi: 10.1371/journal.pone.0181414 (PMC5555574; doi:10.1371/journal.pone.0181414)
Supplement: S2 File — (DOCX) [file pone.0181414.s002.docx]

**S2 File Analysis of results by sample group**

**S2 Fig A: ROC analysis of the results from ELISA of the initial 50 serum samples.**

**S2 Fig A. ROC analysis of the results from ELISA of the initial 50 serum samples with 33 antigen/secondary antibody combinations, by the methods n15 – nx02, as shown in Table 5 and corresponding to values shown in Figure 3.** Standard significance values indicated by stars are for comparisons of ROC results for each method relative to those for method n15. Significance of pairwise differences between AUC values was estimated using the Delong’s test [67] implemented in the pROC R package. Each measurement included in the ROC analysis was carried out in quadruplicate and an average was taken. Numbers on the graphs represent the area under the curve (AUC) of the results with each individual antigen.

**S2 Fig B. The distribution of ELISA absorbances of individual serum samples of first set of 50 patients with smear and culture positive PTB (TB) or culture negative (no TB) as shown in Table 5 (methods n15 – nx02).**

**S2 Fig B. The distribution of ELISA absorbances of individual serum samples of first set of 50 patients with smear and culture positive PTB (TB) or culture negative (no TB).** The bars indicate the medians. In each case the secondary antibody was peroxidase conjugated and the binding was measured by addition of o-phenylenediamine and H_2_O_2_ in citrate buffer and the colour reaction was terminated by the addition of acid. Each individual measurement was carried out in quadruplicate.

**S2 Fig C: ROC analysis of results from ELISA assay of second 50 serum samples with 21 antigen/secondary antibody combinations as presented in Table 6.**

**S2 Fig C. ROC analysis of the results from ELISA of the second set of 50 serum samples with 21 antigen/ secondary antibody combinations, by the methods n15 – n49, as shown in Table 6 and corresponding to values shown in Figure 4.** Standard significance values indicated by stars are for comparisons of ROC results for each method relative to those for method n15. Significance of pairwise differences between AUC values was estimated using the Delong’s test [67]) implemented in the pROC R package. Each measurement included in the ROC analysis was carried out in quadruplicate and an average was taken. Numbers on the graphs represent the area under the curve (AUC) of the results with each individual antigen.

**S2 Fig D: The distribution of responses (absorbances) of individual serum samples of second set of 50 patients with smear and culture positive PTB (TB) or culture negative (no TB) by ELISA using 21 antigen/secondary antibody combinations as shown in Table 6 (methods n15 – n49).**

**S2Fig D. The distribution of responses (absorbances) of individual serum samples of second set of 50 patients with smear and culture positive PTB (TB) or culture negative (no TB).** The bars indicate the medians. In each case the secondary antibody was peroxidase conjugated and the binding was measured by addition of o-phenylenediamine and H_2_O_2_ in citrate buffer and the colour reaction was terminated by the addition of acid. Each individual measurement was carried out in quadruplicate.

**S2 Fig E: ROC analysis of the results from ELISA of combined set of 100 serum samples with 14 antigen/ secondary antibody combinations n15 – n39 as in Table 7**

**S2 Fig E. ROC analysis of the results from ELISA of combined set of 100 serum samples with 14 antigen/ secondary antibody combinations n15 – n39, the results for which are summarised in Table 7 and values are plotted in Figure 3.** In each case the secondary antibody was peroxidase conjugated and the binding was measured by addition of o-phenylenediamine and H_2_O_2_ in citrate buffer and the colour reaction was terminated by the addition of acid. Each measurement was carried out in quadruplicate and an average was taken. Numbers on the graphs represent the area under the curve (AUC) of the results with each individual antigen Significance of pairwise differences between AUC values was estimated using the Delong’s test [67] implemented in the pROC R package. In this case, there was no significance difference between the ROC results for any of the other methods compared to method those for method n15.

**S2 Fig F:** **ROC analysis of results of GBM statistics to combine the results from seven antigen/secondary antibody combinations (n15, n20, n3, n28, n32, n1, n39)** **with the first 100 samples**


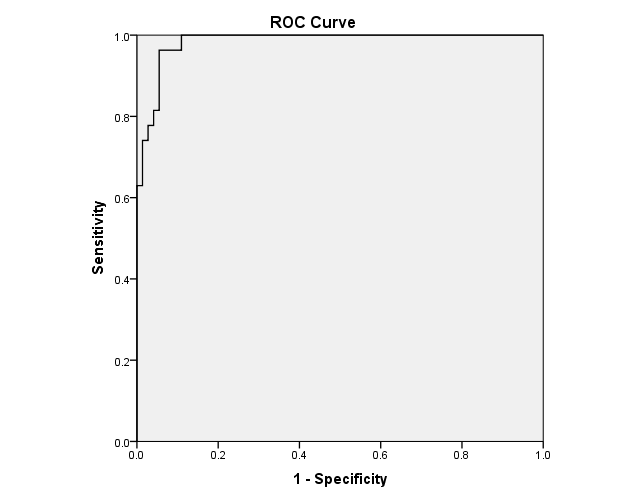


Footnote: The optimum combination of sensitivity and specificity from this analysis was 96/95. Abbreviations: GBM: Generalized Boosted Regression Models

**S2 Fig G: ROC analysis for 249 samples run blind with 8 antigen/secondary antibody combinations n15 – n44, natural TDM from MTB (n15), natural bovine TDM (n20) and six synthetic antigens.**

.

**S2 Fig G. ROC analysis for 249 samples run blind with 7 antigen/secondary antibody combinations n15 – n39, natural TDM from Mtb (n15), natural bovine TDM (n20) and five synthetic antigens.** The medians are in Table 9 and the results are plotted in Figure 5. The assay was run blind and then un-blinded for analysis compared to cut-off values set for the first 100 samples as in Table 7. In each case the secondary antibody was peroxidase conjugated and the binding was measured by addition of o-phenylenediamine and H_2_O_2_ in citrate buffer and the colour reaction was terminated by the addition of acid. Each measurement was carried out quadruplicate and an average was taken. Standard significance values indicated by stars are for comparisons of ROC results for each method relative to those for method n15. Significance of pairwise differences between AUC values was estimated using the Delong’s test [67] implemented in the pROC R package. Also included for comparison, method n44 using free mycolic acid **26** (Table 4) and IgG (whole) at 1:20 serum dilution. Abbreviations: TDM: trehalose dimycolate. Mtb: *Mycobacterium tuberculosis*. Numbers in the graph represent area under curve of the results with each individual antigen.

**S2 Fig H: ROC analysis of results with individual antigen/secondary antibody combinations for smear and culture positive PTB or without TB, culture negative sets for all 349 samples based on clinical diagnosis using TDR protocols, using 8 different antigens, natural TDM from MTB (n15), natural bovine TDM (n20) and six synthetic antigens.**

**S2 Fig H. ROC analysis of results with individual antigen/secondary antibody combinations for smear and culture positive PTB and culture negative sets for all 349 samples. Based on clinical diagnosis using WHO/TDR protocols, using 8 different antigens, natural TDM from Mtb (n15), natural bovine TDM (n20) and six synthetic antigens (combinations summarised in Figure 6 and Table 10).** Also included for comparison, method n44 using free mycolic acid **26** (Table 4) and IgG (whole) at 1:20 serum dilution. In each case the secondary antibody was peroxidase conjugated and the binding was measured by addition of o-phenylenediamine and H_2_O_2_ in citrate buffer and the colour reaction was terminated by the addition of acid. Each measurement was carried out in quadruplicate and an average was taken. Standard significance values indicated by stars are for comparisons of ROC results for each method relative to those for method n15. Significance of pairwise differences between AUC values was estimated using the Delong’s test [67] implemented in the pROC R package
