# Supplementary material for: New synthetic lipid antigens for rapid serological diagnosis of tuberculosis
Source: PLoS One. 2017 Aug 14;12(8):e0181414. doi: 10.1371/journal.pone.0181414 (PMC5555574; doi:10.1371/journal.pone.0181414)
Supplement: S3 File — (DOCX) [file pone.0181414.s003.docx]

**S3 File Analysis of results by sub-groups (where available)**

**Impact of previous BCG vaccination on the serological results.**

In most cases, the BCG vaccination status was recorded (96 of the culture positive PTB samples and 207 of the culture negative samples), though this information was not mandatory for the data collected with the serum set and must be treated with caution. No significant effect of vaccination on the accuracy of the serological results was found with any of the antigens (S11 Fig; S10 Table) except in the case of the culture negative set with the methods n28 and n1 and using the free mycolic acid (n44).

**S3 Table A: Impact of BCG vaccination (where known) on the median values of absorbances in ELISA from 349 sera from smear and culture positive PTB or culture negative (no TB) patients.**

|  |  | |  | **TB** | **No TB** |  |
| --- | --- | --- | --- | --- | --- | --- |
| **Method*** | **Antigen** | | **Secondary Ab** | **Smear and culture positive PTB set^$^** | **Culture negative set** |  |
|  |  |  | | **BCG vs no BCG vaccination**  **p value** | **BCG vs no BCG vaccination**  **p value** |  |
| n15 | **Human TDM** | IgG | | 0.718 | 0.050 |  |
| n20 | **Bovine TDM** | IgG | | 0.285 | 0.899 |  |
| n3 | **6** | IgG (Fc) | | 0.431 | 0.097 |  |
| n28 | **12** | IgG (Fc) | | 0.824 | **0.027** |  |
| n32 | **13** | IgG (Fc) | | 0.747 | 0.840 |  |
| n1 | **14** | IgG (Fc) | | 0.994 | **0.011** |  |
| n39 | **20** | IgG (Fc) | | 0.140 | 0.157 |  |
| n44 | **26** | IgG | | 0.502 | **0.021** |  |

**Footnotes:** Numbers in bold are those that show significant differences (p <.05). Abbreviations: TB: tuberculosis; BCG: bacillus Calmette–Guérin. Ab: antibody. $ 2 of 102 were smear negative.

.

**S3 Fig A: Impact of BCG vaccination (where known) on the serology results (absorbance values) of 349 smear and culture positive PTB (TB) or culture negative (no TB) patients with methods n15 – n44.**

**S3 Fig A. Impact of BCG vaccination on the serology results of smear and culture positive PTB^$^ (TB) or culture negative (no TB), culture negative patients with methods n15 – n44 (using secondary antibodies as described in Table 9) divided according to whether the person providing the serum had, or had not had, a previous BCG vaccination.** In each case the secondary antibody was peroxidase conjugated and the binding was measured by addition of o-phenylenediamine and H_2_O_2_ in citrate **buffer and the colour reaction was terminated by the addition of acid**. Each measurement was carried out in quadruplicate and an average was taken. Those with unknown BCG status were excluded from this analysis. No: no previous BCG; yes: previous vaccination. Blue dots indicate: BCG vaccination; Red dots indicate: no BCG vaccination. BCG vaccination; Red bars indicate: median of the absorbance values of the serological test done with samples from no BCG vaccination. Blue bars indicate: median of the absorbance values of the serological test done with samples from patients reporting BCG vaccination. Abbreviations: TDM: trehalose dimycolate. MTB: *Mycobacterium tuberculosis.* BCG: Bacillus Calmette–Guérin. $ 2 of 102 were smear negative.

**S3 Fig B: Impact of BCG vaccination on the serology results (ROC values) of smear and culture positive PTB or culture negative (no TB) patients with methods n15 – n44**

**^$^**

**S3 Fig B. Impact of BCG vaccination on the serology results (ROC values) of smear and culture positive PTB^$^ or culture negative patients with each method n15 – n44, divided according to whether the person providing the serum had, or had not had, a previous BCG vaccination, as presented in Figure 11s**. In each case the secondary antibody was peroxidase conjugated and the binding was measured by addition of o-phenylenediamine and H_2_O_2_ in citrate buffer and the colour reaction was terminated by the addition of acid. Each measurement was carried out in quadruplicate and an average was taken. Those with unknown BCG status were excluded from this analysis. $ 2 of 102 were smear negative.

**Impact of previous TB on the serological results with different antigens.**

We evaluated the effect of recorded previous TB on the accuracy of the serological results (S12 and S13 Tables; S12 and 13 Figs)**.** The results show that, for all the TDMs, previous TB does not have a significant impact on the accuracy of the results with the culture positive PTB set. However, there was a significant effect on the values for the culture negative samples with three methods (n15, n23, n28). This observation clearly shows that responses to some synthetic antigens are affected to a much smaller extent than are others.

**S3 Table B: Impact of previous TB on the median values of absorbances in ELISA from sera samples from patients with or without current PTB.**

| **Method*** | **Antigen** | **Secondary Ab** | **Smear and culture positive PTB set^$^** | **Culture negative set** |
| --- | --- | --- | --- | --- |
|  |  |  | **“Previous active TB” vs “no previous active TB”** | **“Previous active TB” vs “no previous active TB”** |
|  |  |  | **p value** | **p value** |
| n15 | Human TDM | IgG | 0.752 | **0.014** |
| n20 | Bovine TDM | IgG | 0.177 | 0.067 |
| n3 | 6 | IgG (Fc) | 0.724 | **0.013** |
| n28 | 12 | IgG (Fc) | 0.931 | **0.012** |
| n32 | 13 | IgG (Fc) | 0.795 | 0.335 |
| n1 | 14 | IgG (Fc) | 0.944 | 0.054 |
| n39 | 20 | IgG (Fc) | 0.678 | 0.059 |
| n44 | 26 | IgG | 0.312 | 0.090 |

**Footnotes:** Numbers in bold are those that show significant differences (p <.05). Abbreviations: BCG: bacillus Calmette–Guérin. Ab: antibody. $ 2 of 102 were smear negative.

**S3 Fig C: Impact of previous active TB on the serology results (absorbance values) of smear and culture positive PTB (TB) or culture negative (no TB) patients with methods n15 – n44.**

**S3 Fig C. Impact of previous active TB on the serology results (absorbance values) of smear and culture positive PTB (TB) or culture negative (no TB) patients with each method n15 – n44. Distribution of ELISA responses (absorbances) and medians with each method n15 – n44 (using secondary antibodies as presented in Table 9) divided according to whether the person providing the serum was recorded as having had, or not having had, active TB within the past 5 years. I**n each case the antibody was peroxidase conjugated and the binding was measured by addition of o-phenylenediamine and H_2_O_2_ in citrate buffer and the colour reaction was terminated by the addition of acid. Each measurement was carried out in quadruplicate. Those with unknown previous active TB status were excluded from this analysis. Blue dots indicate: previous active TB; Red dots indicate: no previous active TB. Red bars indicate: median of the absorbance values of the serological test done with samples from no previous active TB patients

.

**S3 Table C: Medians for smear and culture positive PTB or culture negative (no TB) sets for individuals with or without previous active TB in the past 5 years.**

| **Method** | **Culture positive PTB set^$^** | | **Culture negative set** | | |
| --- | --- | --- | --- | --- | --- |
|  | **Previous active TB** | **Median**  **(IQR)** | | **Previous active TB** | **Median**  **(IQR)** |
| n15 | No | 1.46 (0.92 – 2.4) | | No | 3.14 (2.65 – 3.43) |
| n15 | Yes | 1.85 (1.14 – 2.84) | | Yes | 3.18 (2.34 -3.42) |
| n20 | No | 1.36 (0.87 – 2.19) | | No | 3.2 (2.79 -3.45) |
| n20 | Yes | 1.71 (1.05 – 2.82) | | Yes | 3.02 (1.83 – 3.29) |
| n3 | No | 0.84 (0.5 – 1.33) | | No | 3.41 (2.76 – 3.96) |
| n3 | Yes | 1.13 (0.65 – 2.78) | | Yes | 3.39 (1.53 – 3.98) |
| n28 | No | 0.69 (0.41 -1.29) | | No | 3.04 (1.88 – 3.42) |
| n28 | Yes | 0.9 (0.55 – 2.03) | | Yes | 3.03 (1.01 – 3.57) |
| n32 | No | 0.67 (0.42 – 1.1) | | No | 2.86 (1.16 – 3.24) |
| n32 | Yes | 0.88 (0.42 – 1.65) | | Yes | 2.66 (0.79 – 3.42) |
| n1 | No | 0.63 (0.4 – 1.14) | | No | 3.23 (2.24 – 3.67) |
| n1 | Yes | 0.74 (0.46 – 1.66) | | Yes | 3.67 (1.1 – 3.89) |
| n39 | No | 0.51 (0.32 – 0.93) | | No | 3.36 (2.43 – 3.87) |
| n39 | Yes | 0.64 (0.41 – 1.27) | | Yes | 3.3 (1.35 – 4.04) |
| n44 | No | 1.08 (0.65 – 1.53) | | No | 1.19 (0.66 – 1.75) |
| n44 | Yes | 0.80 (0.44 – 1.43) | | Yes | 1.38 (0.91 – 2.37) |

**S3 Table C: Medians by previous active TB.** First column shows the method. Second and fifth columns show record of previous active TB (yes) or no previous active TB (no) for samples having culture positive PTB (column 2) and being culture negative (column 5). Footnotes: iqr: inter-quartile ratio; lq lower quartile limit, uq upper quartile limit. $ 2 of 102 were smear negative.

**S3 Fig D: Impact of previous active TB on the serology results (ROC values) of smear and culture positive PTB or culture negative patients with methods n15 – n44.**

**S3 Fig D. Impact of previous active TB on the serology results (ROC values) of serum and culture positive PTB^$^ or culture negative patients with each method n15 – n44.** ROC analysis of ELISA responses (absorbances) for 349 samples with each method n15 – n44, natural TDM from MTB (n15), natural bovine TDM (n20) and six synthetic antigens divided according to whether the person providing the serum had, or had not had, active TB in the previous 5 years as presented in Figure 13s. In each case the secondary antibody was peroxidase conjugated and the binding was measured by addition of o-phenylenediamine and H_2_O_2_ in citrate buffer and the colour reaction was terminated by the addition of acid. Each measurement was carried out in quadruplicate and an average was taken. Those with unknown previous active TB status were excluded from this analysis. Significance of pairwise differences. $ 2 of 102 were smear negative.

**Impact effect of a positive TST test on the serological results.**

TST was recorded only for 113 subjects. The results from this limited set are analysed in S15 Fig and S15 Table. The analysis assesses wherether there is an association between TST test results and antigen levels in culture positive and culture negative individuals. Largely there was not except for significant differences in n15 and n20 antigen levels for culture negative individuals.

**S3 Table D: Impact of a positive or negative TST on the median values of absorbances in ELISA from sera samples from smear and culture positive PTB and culture negative patients**

| **Method*** | **Antigen** | **Secondary Ab** | **Smear and culture positive**  **PTB set^$^** | **Culture negative**  **set** |
| --- | --- | --- | --- | --- |
|  |  |  | **Positive TST vs negative TST**  **p value** | **Positive TST vs negative TST**  **p value** |
| n15 | Human TDM | IgG | 0.953 | **0.038** |
| n20 | Bovine TDM | IgG | 0.634 | **0.019** |
| n3 | 6 | IgG (Fc) | 0.308 | 0.314 |
| n28 | 12 | IgG (Fc) | 0.296 | 0.794 |
| n32 | 13 | IgG (Fc) | 0.570 | 0.121 |
| n1 | 14 | IgG (Fc) | 0.906 | 0.183 |
| n39 | 20 | IgG (Fc) | 0.820 | 0.557 |
| n44 | 26 | IgG | 0.912 | 0.833 |

**Footnotes:** Numbers in bold are those that show significant differences (p <.05). Abbreviations: TB: tuberculosis; BCG: bacillus Calmette–Guérin. Ab: antibody. $ 2 of 102 were smear negative.

**S3 Fig E: Impact of a positive or negative TST on the serology results (ROC values) of smear and culture positive PTB and culture negative patients with each method n15 – n44.**

**S3 Fig E. Impact of a positive or negative TST on the serology results (ROC values) of smear and culture positive PTB^$^ and culture negative patients with each method n15 – n44.** ROC analysis ELISA responses (absorbances) for culture negative samples from set of 349 with positive TST test (3) compared to that for a negative TST test (2), in each case compared to culture positive PTB samples, for responses with each method n15 – n44 (using secondary antibody combinations as presented in Table 9). In each case the secondary antibody was peroxidase conjugated and the binding was measured by addition of o-phenylenediamine and H_2_O_2_ in citrate buffer and the colour reaction was terminated by the addition of acid. Each measurement was carried out in quadruplicate and an average was taken. Those with unknown TST status were excluded from this analysis. $ 2 of 102 were smear negative

**Impact of other diseases on the serological results.**

The sample set included significant numbers of patients who were recorded as co-infected with other diseases or having other medical conditions, though this information was not mandatory for the data collected with the serum set and must be treated with caution. Although there are some small differences in median response within both culture positive PTB and culture negative sets, it does not appear that co-infection with malaria or diseases such as diabetes or chronic obstructive pulmonary disease (COPD) affects the ELISA responses of the majority of the antigens (S 16 – 18 Tables ; S 16 and S 17 Figs). However, there were statistically significant differences between culture positive individuals with and without co-existing diseases for method n44 (free mycolic acid) and for culture negative individuals differences for methods n20 and n28.

**S3** **Fig F: Impact of concurrent diseases different from TB on the serology results (absorbance values) of smear and culture positive PTB (TB) and culture negative (no TB) patients with methods n15 – n44.**

**S3 Fig F. Impact of concurrent diseases different from active TB on the serology results (absorbance values) of smear and culture positive PTB (TB)^$^ and culture negative (no TB) patients with each method n15 – n44.** Effect of other diseases on median ELISA responses for culture positive PTB and culture negative sets with all 349 samples with each method (using secondary antibody combinations as presented in Table 9). In each case the secondary antibody was peroxidase conjugated and the binding was measured by addition of o-phenylenediamine and H_2_O_2_ in citrate buffer and the colour reaction was terminated by the addition of acid. Each measurement was carried out in quadruplicate and an average was taken. Abbreviations TB: tuberculosis. TDM: trehalose dimycolate. MTB: *Mycobacterium tuberculosis;* COPD: chronic obstructive pulmonary disease. $ 2 of 102 were smear negative.

**S3 Table E: Medians for smear and culture positive PTB and culture negative (no TB) and co-existing disease status**

|  |  | **No TB** | | | **TB** | | | |
| --- | --- | --- | --- | --- | --- | --- | --- | --- |
|  |  | **Culture negative set** | | | **Smear and culture positive set^$^** | | | |
| **Method** | **Co-existing**  **Illness** | **median** | **lq** | **uq** | **median** | **lq** | **uq** | |
| n15 | None | 1.51 | 0.95 | 2.46 | 3.14 | 2.58 | 3.45 | |
| n15 | Malaria | 1.96 | 1.19 | 2.82 | 3.26 | 2.80 | 3.38 | |
| n15 | Diabetes | 1.14 | 0.80 | 1.91 | 2.60 | 2.21 | 2.90 | |
| n15 | Copd | 0.99 | 0.62 | 1.65 |  |  |  | |
| n15 | Other | 1.42 | 0.96 | 2.22 | 3.25 | 2.40 | 3.39 | |
| n15 | NA |  |  |  | 2.99 | 2.99 | 2.99 | |
| n20 | None | 1.62 | 1.08 | 2.39 | 3.18 | 2.80 | 3.43 | |
| n20 | Malaria | 1.28 | 0.81 | 1.94 | 3.17 | 2.71 | 3.37 | |
| n20 | Diabetes | 1.18 | 0.74 | 1.94 | 2.50 | 1.76 | 3.19 | |
| n20 | Copd | 1.01 | 0.39 | 1.85 |  |  |  | |
| n20 | Other | 1.27 | 0.61 | 2.11 | 3.33 | 2.16 | 3.43 | |
| n20 | NA |  |  |  | 3.11 | 3.11 | 3.11 | |
| n3 | None | 0.86 | 0.49 | 1.61 | 3.36 | 2.39 | 3.89 | |
| n3 | Malaria | 1.15 | 0.72 | 2.19 | 3.78 | 3.02 | 4.01 | |
| n3 | Diabetes | 0.60 | 0.41 | 0.93 | 3.60 | 2.72 | 3.95 | |
| n3 | Copd | 0.58 | 0.36 | 0.97 |  |  |  | |
| n3 | Other | 0.72 | 0.47 | 1.17 | 3.19 | 1.67 | 4.08 | |
| n3 | NA |  |  |  | 3.04 | 3.04 | 3.04 | |
| n28 | None | 0.63 | 0.36 | 1.18 | 3.07 | 1.43 | 3.41 | |
| n28 | Malaria | 1.19 | 0.61 | 1.90 | 2.98 | 2.47 | 3.53 | |
| n28 | Diabetes | 0.40 | 0.36 | 0.88 | 2.59 | 1.60 | 3.24 | |
| n28 | Diabetes |  |  |  |  |  |  | |
| n28 | Copd | 0.67 | 0.43 | 0.96 |  |  |  | |
| n28 | Other | 0.68 | 0.47 | 1.37 | 3.14 | 1.82 | 3.57 | |
| n28 | NA |  |  |  | 3.03 | 3.03 | 3.03 | |
| n32 | None | 0.69 | 0.44 | 1.17 | 2.83 | 1.00 | 3.26 | |
| n32 | Malaria | 1.05 | 0.72 | 2.01 | 2.61 | 1.27 | 3.06 | |
| n32 | Diabetes | 0.42 | 0.31 | 0.53 | 2.44 | 1.30 | 3.42 | |
| n32 | Copd | 0.37 | 0.27 | 0.62 |  |  |  | |
| n32 | Other | 0.50 | 0.32 | 0.89 | 3.09 | 2.16 | 3.56 | |
| n32 | NA |  |  |  | 2.92 | 2.92 | 2.92 | |
| n1 | None | 0.69 | 0.40 | 1.18 | 3.20 | 2.04 | 3.70 | |
| n1 | Malaria | 0.97 | 0.55 | 1.39 | 3.13 | 2.54 | 3.47 | |
| n1 | Diabetes | 0.46 | 0.36 | 0.71 | 3.33 | 2.66 | 3.47 | |
| n1 | Copd | 0.41 | 0.29 | 0.71 |  |  |  | |
| n1 | Other | 0.54 | 0.35 | 0.90 | 3.68 | 2.77 | 4.01 | |
| n1 | NA |  |  |  | 3.60 | 3.60 | 3.60 | |
| n39 | None | 0.57 | 0.32 | 1.01 | 3.36 | 2.27 | 3.90 | |
| n39 | Malaria | 0.63 | 0.41 | 1.28 | 3.31 | 2.98 | 3.72 | |
| n39 | Diabetes | 0.41 | 0.29 | 0.64 | 3.70 | 2.65 | 4.09 | |
| n39 | Copd | 0.39 | 0.20 | 0.56 |  |  |  | |
| n39 | Other | 0.50 | 0.29 | 0.85 | 3.06 | 1.92 | 3.83 | |
| n39 | NA |  |  |  | 3.49 | 3.49 | 3.49 | |
| n44 | None | 1.02 | 0.72 | 1.42 | 1.06 | 0.63 | 1.68 | |
| n44 | Malaria | 1.49 | 0.83 | 2.36 | 1.66 | 1.22 | 2.33 | |
| n44 | Diabetes | 0.79 | 0.66 | 1.04 | 1.82 | 0.89 | 2.84 |  |
| n44 | Copd | 0.61 | 0.39 | 0.80 |  |  |  |  |
| n44 | Other | 0.82 | 0.53 | 1.21 | 1.20 | 0.90 | 1.86 |  |
| n44 | NA |  |  |  | 2.28 | 2.28 | 2.28 |  |

**S3 Table E: Medians by smear and culture positive PTB and culture negative and co-existing disease status.**

$ 2 of 102 were smear negative.

**S3 Table F: Impact of diseases different from TB on the median values of absorbances in ELISA from sera samples from smear and culture positive PTB and culture negative patients**

| **Method*** | **Antigen** | **Secondary Ab** | **Smear and culture positive PTB set^$^** | **Culture negative set** |
| --- | --- | --- | --- | --- |
|  |  |  | **Presence or absence of diseases different from TB****  **p value** | **Presence or absence of diseases different from TB****  **p value** |
| n15 | Human TDM | IgG | 0.929 | 0.492 |
| n20 | Bovine TDM | IgG | 0.741 | **0.002** |
| n3 | 6 | IgG (Fc) | 0.389 | 0.526 |
| n28 | 12 | IgG (Fc) | 0.599 | **0.003** |
| n32 | 13 | IgG (Fc) | 0.573 | 0.958 |
| n1 | 14 | IgG (Fc) | 0.661 | 0.753 |
| n39 | 20 | IgG (Fc) | 0.909 | 0.949 |
| n44 | 26 | IgG | **0.029** | 0.880 |

**Footnotes:** **Diseases different from TB: Malaria, chronic obstructive pulmonary diseases; diabetes and other current diseases**.** Numbers in bold are those that show significant differences (p <.05). Abbreviations**:** TB: tuberculosis; BCG: bacillus Calmette–Guérin. Ab: antibody. $ 2 of 102 were smear negative.

**S3 Table G: Impact of concurrent diseases on the median values of absorbances for ELISA in smear and culture positive PTB and culture negative patients for “method n39”.**

| **Clinical status** | **Median values of the ELISA absorbances**  **in smear and culture positive PTB set^$^** | **Median values of the ELISA absorbances**  **in culture negative set** |  |
| --- | --- | --- | --- |
| No identified concurrent disease | 3.36 | 0.57 |  |
| Malaria | 3.31 | 0.63 |  |
| Diabetes | 3.70 | 0.41 |  |
| COPD | **-** | 0.39 |  |

**Footnote:** Abbreviations: COPD: [Chronic obstructive pulmonary disease](https://www.google.co.uk/url?sa=t&rct=j&q=&esrc=s&source=web&cd=3&cad=rja&uact=8&sqi=2&ved=0ahUKEwiF2fm8hPfLAhVGqg4KHeOeBtwQFgg2MAI&url=http%3A%2F%2Fwww.nhs.uk%2FConditions%2FChronic-obstructive-pulmonary-disease%2FPages%2FIntroduction.aspx&usg=AFQjCNGU6ZSU686o_D-vCtmzadXH2LWs7Q&bvm=bv.118443451,d.d24). $ 2 of 102 were smear negative.

**S3 Fig G: Impact of concurrent diseases different from active TB on the serology results (ROC values) of smear and culture positive PTB and culture negative patients with methods n15 – n44.**

**S3 Fig G: Impact of concurrent diseases different from active TB on the serology results (ROC values) of smear and culture positive PTB^$^ and culture negative patients with each method n15 – n44.** Effect of other diseases on ROC analysis of ELISA responses for all 349 samples for culture positive PTB and culture negative sets with each method n15 – n44 (using secondary antibody combinations as presented in Table 9). In each case the secondary antibody was peroxidase conjugated and the binding was measured by addition of o-phenylenediamine and H_2_O_2_ in citrate buffer and the colour reaction was terminated by the addition of acid. Each measurement was carried out in quadruplicate and an average was taken. Significance of pairwise differences between AUC values for the set with no co-infection compared to the set with a co-infection was estimated using the Delong’s test [67]) implemented in the pROC R package. In this case, there was no significance difference between the ROC results for the two sets for any of the methods. Abbreviations: TDM: trehalose dimycolate. MTB: *Mycobacterium tuberculosis;* COPD: chronic obstructive pulmonary disease. $ 2 of 102 were smear negative.

**Impact of country of origin on the serological results.**

Among the culture negative samples, a significant difference in the medians was found depending on the country of origin with all the antigens tested, whereas no significant differences were found among those with “active TB” except for the free mycolic acid n44 (S18and S19 Figs; Table 19). There are significant country-to-country variations in these results; however, this must be treated with caution, as the sample sets for some countries are very small.

**S3 Table H: Impact of country of origin on the median values of absorbances in ELISA from sera samples from smear and culture positive PTB or culture negative patients**

|  |  |  | **TB** | **No TB** |
| --- | --- | --- | --- | --- |
| **Method*** | **Antigen** | **Secondary Ab** | **Smear and culture**  **positive set^$^** | **Culture negative set** |
|  |  |  | **p value** | **p value** |
| n15 | Human TDM | IgG | 0.993 | <.001 |
| n20 | Bovine TDM | IgG | 0.878 | <.001 |
| n3 | 6 | IgG (Fc) | 0.395 | <.001 |
| n28 | 12 | IgG (Fc) | 0.979 | <.001 |
| n32 | 13 | IgG (Fc) | 0.878 | <.001 |
| n1 | 14 | IgG (Fc) | 0.981 | <.001 |
| n39 | 20 | IgG (Fc) | 0.524 | 0.0045 |
| n44 | 26 | IgG | **0.023** | <.001 |

**Footnotes:** Numbers in bold are those that show significant differences (p <.05). $ 2 of 102 were smear negative.

**S3 Fig H: Impact of country of origin on the serology results (absorbance values) of smear and culture positive PTB (TB) and culture negative (no TB) patients with methods n15 – n44.**

**S3 Fig H: Impact of country of origin on the serology results (absorbance values) of smear and culture positive PTB (TB)^$^ and culture negative (no TB) patients with each method n15 – n44.** Variations in median ELISA responses (absorbances) for all 349 samples for culture positive PTB and culture negative sets by country with each method (using secondary antibody combinations as presented in Table 9). In each case the antibody was peroxidase conjugated and the binding was measured by addition of o-phenylenediamine and H_2_O_2_ in citrate buffer and the colour reaction was terminated by the addition of acid. Each measurement was carried out in quadruplicate and an average was taken. The values of the medians and the IQRs are given S19 Table. $ 2 of 102 were smear negative.

**S3 Fig I: Impact of country of origin on the serology results (ROC values) of smear and culture positive PTB and culture negative patients with methods n15 – n44.**

.

**S3 Fig I: Impact of country of origin on the serology results (ROC values) of smear and culture positive PTB^$^ and culture negative patients with each method n15 – n44.** ROC analysis of ELISA responses (absorbances) for all 349 samples for culture positive PTB and culture negative sets by country with each method (using secondary antibody combinations as presented in Table 9). In each case the antibody was peroxidase conjugated and the binding was measured by addition of o-phenylenediamine and H_2_O_2_ in citrate buffer and the colour reaction was terminated by the addition of acid. Each measurement was carried out in quadruplicate. The values of the medians and the IQRs are given in S19 Tables (note that for some cohorts, the numbers are rather small) with each antigen. $ 2 of 102 were smear negative.

**S3 Table I: Medians by country for each antigen/method**

|  |  | **TB** | | |  |  | | **No TB** | | |  |  |
| --- | --- | --- | --- | --- | --- | --- | --- | --- | --- | --- | --- | --- |
| **Method** | **Country of origin** | **Smear and culture positive PTB^$^** | | |  |  | | **Culture negative** | | |  |  |
|  |  | **median** | **lq** | **uq** | | | **median** | | **Lq** | **Uq** | | |
| n15 | Bangladesh | 3.14 | 2.58 | 3.43 | | | 2.15 | | 1.64 | 2.48 | | |
| n15 | Brazil |  |  |  | | | 2.47 | | 2.46 | 3.04 | | |
| n15 | Canada |  |  |  | | | 1.37 | | 0.87 | 2.34 | | |
| n15 | Colombia | 3.21 | 2.73 | 3.34 | | | 1.17 | | 0.81 | 2.18 | | |
| n15 | Gambia | 3.2 | 2.9 | 3.41 | | | 2.17 | | 1.24 | 2.82 | | |
| n15 | Kenya | 3.13 | 2.85 | 3.42 | | | 0.97 | | 0.81 | 1.12 | | |
| n15 | Peru | 2.98 | 2.56 | 3.31 | | | 1.83 | | 1.62 | 2.35 | | |
| n15 | South Africa | 3.18 | 2.22 | 3.53 | | | 1.42 | | 1.24 | 1.91 | | |
| n15 | Spain | 3.16 | 2.65 | 3.36 | | | 1.25 | | 0.97 | 1.78 | | |
| n15 | Vietnam | 3.22 | 2.08 | 3.57 | | | 2.77 | | 2.65 | 2.95 | | |

$ 2 of 102 were smear negative.

|  |  | **TB** | |  |  | **No TB** | | |  | |  |
| --- | --- | --- | --- | --- | --- | --- | --- | --- | --- | --- | --- |
| **Method** | **Country of origin** | **Smear and culture positive PTB^$^** | |  |  | **Culture negative** | | |  | |  |
|  |  | **Median** | **lq** | | **uq** | | **median** | **Lq** | | **Uq** | |
| n20 | Bangladesh | 3.03 | 2.83 | | 3.38 | | 2.02 | 1.37 | | 2.36 | |
| n20 | Brazil |  |  | |  | | 1.40 | 1.27 | | 1.89 | |
| n20 | Canada |  |  | |  | | 0.58 | 0.46 | | 1.03 | |
| n20 | Colombia | 3.18 | 3.1 | | 3.23 | | 1.41 | 0.95 | | 2.28 | |
| n20 | Gambia | 3.1 | 2.63 | | 3.34 | | 1.39 | 0.82 | | 2.01 | |
| n20 | Kenya | 3.33 | 2.92 | | 3.6 | | 0.78 | 0.67 | | 0.88 | |
| n20 | Peru | 3.18 | 2.59 | | 3.35 | | 1.96 | 1.75 | | 2.34 | |
| n20 | South Africa | 3.09 | 2.04 | | 3.45 | | 1.44 | 1.3 | | 1.92 | |
| n20 | Spain | 3.25 | 2.79 | | 3.39 | | 1.9 | 1.39 | | 2.46 | |
| n20 | Vietnam | 3.05 | 1.78 | | 3.33 | | 2.89 | 2.78 | | 3.23 | |

$ 2 of 102 were smear negative.

|  |  | **TB** | | |  |  | **No TB** | | |  | |  |
| --- | --- | --- | --- | --- | --- | --- | --- | --- | --- | --- | --- | --- |
| **Method** | **Country of origin** | **Smear and culture positive PTB^$^** | | |  |  | **Culture negative** | | |  | |  |
|  |  | **median** | **lq** | **uq** | | | | **median** | **Lq** | | **Uq** | |
| n44 | Bangladesh | 0.66 | 0.6 | 1.15 | | | | 0.34 | 0.3 | | 0.79 | |
| n44 | Brazil |  |  |  | | | | 0.82 | 0.65 | | 0.86 | |
| n44 | Canada |  |  |  | | | | 0.49 | 0.39 | | 0.65 | |
| n44 | Colombia | 0.91 | 0.83 | 2.72 | | | | 1.21 | 0.92 | | 1.63 | |
| n44 | Gambia | 1.7 | 1.4 | 2.33 | | | | 1.5 | 0.94 | | 2.36 | |
| n44 | Kenya | 1.56 | 0.45 | 2.14 | | | | 0.25 | 0.25 | | 0.25 | |
| n44 | Peru | 1.45 | 0.57 | 1.73 | | | | 0.65 | 0.58 | | 0.74 | |
| n44 | South Africa | 1.21 | 0.95 | 1.74 | | | | 1.03 | 0.8 | | 1.17 | |
| n44 | Spain | 1.87 | 1.05 | 2.73 | | | | 0.92 | 0.76 | | 1.18 | |
| n44 | Vietnam | 1.14 | 0.85 | 2.21 | | | | 0.77 | 0.69 | | 1.13 | |
|  |  |  |  |  | | | |  |  | |  | |

$ 2 of 102 were smear negative.

|  |  | **TB** |  |  | **No TB** |  |  |
| --- | --- | --- | --- | --- | --- | --- | --- |
| **Method** | **Country of origin** | **Smear and culture positive PTB^$^** |  |  | **Culture negative** |  |  |
|  |  | **median** | **lq** | **uq** | **median** | **Lq** | **Uq** |
| n39 | Bangladesh | 3.34 | 2.58 | 3.77 | 0.83 | 0.67 | 1.6 |
| n39 | Brazil |  |  |  | 0.9 | 0.63 | 2.87 |
| n39 | Canada |  |  |  | 0.38 | 0.24 | 0.7 |
| n39 | Colombia | 3.37 | 3.25 | 4.03 | 0.51 | 0.28 | 0.97 |
| n39 | Gambia | 3.55 | 3.15 | 3.9 | 0.62 | 0.41 | 1.28 |
| n39 | Kenya | 3.17 | 2.83 | 3.5 | 0.63 | 0.63 | 0.63 |
| n39 | Peru | 3.68 | 1.72 | 4.17 | 1.11 | 0.51 | 1.74 |
| n39 | South Africa | 2.2 | 1.42 | 3.71 | 0.51 | 0.35 | 0.64 |
| n39 | Spain | 3.37 | 2.39 | 3.77 | 0.46 | 0.33 | 0.62 |
| n39 | Vietnam | 3.61 | 1.92 | 4.08 | 1.52 | 1 | 2.64 |

$ 2 of 102 were smear negative.

|  |  | **TB** | |  | |  | | **No TB** | |  |  |
| --- | --- | --- | --- | --- | --- | --- | --- | --- | --- | --- | --- |
| **Method** | **Country of origin** | | **Smear and culture positive PTB^$^** | |  | |  | | **Culture negative** |  |  |
|  |  | **median** | | **lq** | | **uq** | | | **Median** | **Lq** | **Uq** |
| n1 | Bangladesh | 3.23 | | 2.04 | | 3.78 | | | 0.87 | 0.69 | 1.25 |
| n1 | Brazil |  | |  | |  | | | 1.03 | 0.71 | 2.48 |
| n1 | Canada |  | |  | |  | | | 0.4 | 0.28 | 0.74 |
| n1 | Colombia | 3.21 | | 3.05 | | 3.47 | | | 0.58 | 0.38 | 1.1 |
| n1 | Gambia | 3.27 | | 2.66 | | 3.64 | | | 1.02 | 0.59 | 1.41 |
| n1 | Kenya | 3.3 | | 2.96 | | 3.79 | | | 0.5 | 0.48 | 0.52 |
| n1 | Peru | 3.32 | | 1.58 | | 3.55 | | | 0.95 | 0.62 | 1.34 |
| n1 | South Africa | 3.39 | | 2.16 | | 3.94 | | | 0.69 | 0.57 | 1.05 |
| n1 | Spain | 2.63 | | 1.82 | | 3.33 | | | 0.57 | 0.36 | 0.73 |
| n1 | Vietnam | 3.14 | | 1.59 | | 3.75 | | | 1.5 | 1.15 | 2.77 |

$ 2 of 102 were smear negative.

|  |  |  | **TB** |  | **No TB** |  | |  | |
| --- | --- | --- | --- | --- | --- | --- | --- | --- | --- |
| **Method** | **Country of origin** | **Smear and culture positive PTB^$^** |  |  | **Culture negative** |  | |  | |
|  |  | **Median** | **lq** | **uq** | **median** | **Lq** | **Uq** | |  |
| n32 | Bangladesh | 2.83 | 0.74 | 3.14 | 0.63 | 0.56 | 0.99 | |  |
| n32 | Brazil |  |  |  | 0.62 | 0.54 | 1.25 | |  |
| n32 | Canada |  |  |  | 0.31 | 0.23 | 0.47 | |  |
| n32 | Colombia | 3.09 | 1.51 | 3.37 | 0.74 | 0.42 | 1.29 | |  |
| n32 | Gambia | 2.83 | 1.89 | 3.09 | 1.04 | 0.75 | 2.03 | |  |
| n32 | Kenya | 2.44 | 1.16 | 2.89 | 0.91 | 0.71 | 1.11 | |  |
| n32 | Peru | 2.93 | 1.34 | 3.23 | 0.45 | 0.41 | 0.47 | |  |
| n32 | South Africa | 2.12 | 0.97 | 3.55 | 0.91 | 0.56 | 1.33 | |  |
| n32 | Spain | 2.28 | 0.61 | 3.91 | 0.48 | 0.34 | 0.73 | |  |
| n32 | Vietnam | 3.16 | 1.31 | 3.53 | 0.94 | 0.88 | 2.93 | |  |

$ 2 of 102 were smear negative.

|  |  | **TB** |  | |  | | **No TB** | | |  | |  |
| --- | --- | --- | --- | --- | --- | --- | --- | --- | --- | --- | --- | --- |
| **Method** | **Country of origin** | **Smear and culture positive PTB^$^** | |  | |  | | **Culture negative** |  | | |  |
|  |  | **Median** | | **lq** | | **uq** | | **median** | **Lq** | | **Uq** | |
| n28 | Bangladesh | 2.79 | | 1.4 | | 3.48 | | 0.8 | 0.63 | | 1.28 | |
| n28 | Brazil |  | |  | |  | | 0.85 | 0.84 | | 1.18 | |
| n28 | Canada |  | |  | |  | | 0.69 | 0.43 | | 1.46 | |
| n28 | Colombia | 3.13 | | 2 | | 3.32 | | 0.45 | 0.3 | | 0.88 | |
| n28 | Gambia | 2.98 | | 2.58 | | 3.51 | | 1.18 | 0.62 | | 1.91 | |
| n28 | Kenya | 3.03 | | 2.45 | | 3.47 | | 0.84 | 0.56 | | 1.11 | |
| n28 | Peru | 3.09 | | 0.94 | | 3.41 | | 0.81 | 0.42 | | 1.39 | |
| n28 | South Africa | 2.03 | | 1.75 | | 3.55 | | 0.67 | 0.6 | | 0.95 | |
| n28 | Spain | 3.49 | | 2.64 | | 3.69 | | 0.67 | 0.47 | | 1.02 | |
| n28 | Vietnam | 3.14 | | 2.31 | | 3.25 | | 2.34 | 1.42 | | 2.91 | |

$ 2 of 102 were smear negative.

|  |  | **TB** |  | | |  | | | **No TB** | |  | | |  |
| --- | --- | --- | --- | --- | --- | --- | --- | --- | --- | --- | --- | --- | --- | --- |
| **Method** | **Country of origin** | **Smear and culture positive PTB^$^** | | |  | | |  | | **Culture negative** | |  | |  |
|  |  | **median** | | **lq** | | | **Uq** | | | **median** | **Lq** | | **Uq** | |
| n3 | Bangladesh | 3.14 | | 2.85 | | | 3.61 | | | 1.09 | 0.82 | | 1.87 | |
| n3 | Brazil |  | |  | | |  | | | 1.27 | 1.13 | | 3.29 | |
| n3 | Canada |  | |  | | |  | | | 0.52 | 0.35 | | 1 | |
| n3 | Colombia | 3.53 | | 3.28 | | | 4.01 | | | 0.77 | 0.4 | | 1.34 | |
| n3 | Gambia | 4 | | 3.32 | | | 4.16 | | | 1.16 | 0.73 | | 2.19 | |
| n3 | Kenya | 3.21 | | 2.94 | | | 3.8 | | | 0.62 | 0.45 | | 0.79 | |
| n3 | Peru | 3.5 | | 1.72 | | | 3.78 | | | 1.83 | 1.37 | | 2.21 | |
| n3 | South Africa | 2.87 | | 1.63 | | | 3.98 | | | 0.79 | 0.67 | | 1.02 | |
| n3 | Spain | 3.06 | | 2.41 | | | 3.21 | | | 0.78 | 0.52 | | 0.98 | |
| n3 | Vietnam | 3.84 | | 2.69 | | | 4.02 | | | 2.13 | 1.99 | | 3.73 | |

$ 2 of 102 were smear negative.
